# Design, Synthesis, Biological Evaluation, and Molecular Docking Studies of Novel 1,3,4-Thiadiazole Derivatives Targeting Both Aldose Reductase and α-Glucosidase for Diabetes Mellitus

**DOI:** 10.1021/acsomega.5c00566

**Published:** 2025-05-05

**Authors:** Betül Kaya, Ulviye Acar Çevik, Adem Necip, Hatice Esra Duran, Bilge Çiftçi, Mesut Işık, Pervin Soyer, Hayrani Eren Bostancı, Zafer Asım Kaplancıklı, Şükrü Beydemir

**Affiliations:** † Department of Pharmaceutical Chemistry, Faculty of Pharmacy, Zonguldak Bulent Ecevit University, 67600 Zonguldak, Turkey; ‡ Department of Pharmaceutical Chemistry, Faculty of Pharmacy, Anadolu University, 26470 Eskişehir, Turkey; § Department of Pharmacy Services, Vocational School of Health Services, Harran University, 63300 Şanlıurfa, Turkey; ∥ Department of Medical Biochemistry, Faculty of Medicine, Kafkas University, 36100 Kars, Turkey; ⊥ Vocational School of Health Services, Bilecik Şeyh Edebali University, 11230 Bilecik, Turkey; # Department of Bioengineering, Faculty of Engineering, Bilecik Şeyh Edebali University, 11230 Bilecik, Turkey; ∇ Department of Pharmaceutical Microbiology, Faculty of Pharmacy, Anadolu University, 26470 Eskişehir, Turkey; ○ Department of Biochemistry, Faculty of Pharmacy, Cumhuriyet University, 58140 Sivas, Turkey; ◆ Department of Biochemistry, Faculty of Pharmacy, Anadolu University, 26470 Eskişehir, Turkey; ¶ The Rectorate of Bilecik Şeyh Edebali University, 11230 Bilecik, Turkey

## Abstract

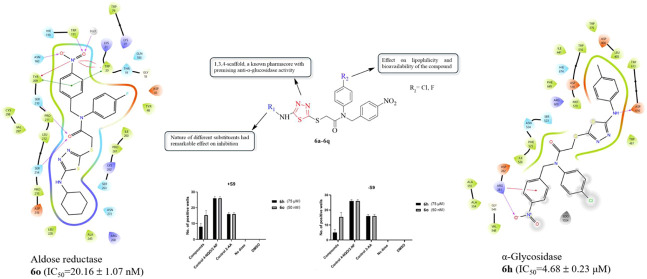

We have developed new 1,3,4-thiadiazole derivatives and examined their ability to inhibit aldose reductase and α-glucosidase. All of the members of the series showed a higher potential of aldose reductase inhibition (*K*_I_: 15.39 ± 1.61–176.50 ± 10.69 nM and IC_50_: 20.16 ± 1.07–175.40 ± 6.97 nM) compared to the reference inhibitor epalrestat (*K*_I_: 837.70 ± 53.87 nM, IC_50_: 265.00 ± 2.26 nM). Furthermore, compounds **6a**, **6g**, **6h**, **6j**, **6o**, **6p**, and **6q** showed significantly higher inhibitory activity (*K*_I_: 4.48 ± 0.25 μM–15.86 ± 0.92 μM and IC_50_: 4.68 ± 0.23 μM–34.65 ± 1.78 μM) toward α-glucosidase compared to the reference acarbose (*K*_I_: 21.52 ± 2.72 μM, IC_50_: 132.51 ± 9.86 μM). Molecular docking studies confirmed that the most potent inhibitor of α-GLY, compound **6h** (*K*_*I*_: 4.48 ± 0.25 μM), interacts with the target protein 5NN8 through hydrogen bonds as in acarbose. On the other hand, compounds **6o** (*K*_I_: 15.39 ± 1.61 nM) and **6p** (*K*_I_: 23.86 ± 2.41 nM), the most potent inhibitors for AR, establish hydrogen bonds with the target protein 4JIR like epalrestat. *In silico* ADME/T analysis was performed to predict their drug-like properties. A cytotoxicity study was carried out with the L929 fibroblast cell line *in vitro*, revealing that all of the synthesized compounds were noncytotoxic. Furthermore, AMES test has been added to show the low mutagenic potential of the compounds **6h** and **6o**.

## 1 Introduction

Diabetes Mellitus (DM) is a well-known growing metabolic disease characterized by the development of hyperglycemia due to insufficient insulin production. Hyperglycemia in DM is known to play a significant role with other systemic factors for the onset and development of diabetic retinopathy, nephropathy, and neuropathy.^1,2^ DM manifests in mainly two subtypes: Type 1 (T1DM) and type 2 (T2DM), which is responsible for approximately 90% of all patients with DM.^3,4^ The global diabetes prevalence is rising at an alarming rate; in 2045, it is estimated to be 783 million people with DM.^5,6^ Recently, prevalent diabetes has been ranked third among chronic diseases after cardiovascular and tumor diseases.^7^

Inhibition of enzymes involved in carbohydrate digestion (α-(α)-amylases and α-glucosidases) to slow postprandial hyperglycemia is one of the most common strategies to manage diabetes.^8,9^ α-Glucosidase (EC 3.2.1.20) is a carbohydrate hydrolase that catalyzes the hydrolysis of the 1,4-α-glycosidic bond, releasing monosaccharides from carbohydrates.^10^ Inhibitors of α-glucosidase (α-GLY), in this regard, can prolong the process of carbohydrate absorption in the gastrointestinal tract. Therefore, they are considered one of the safest strategies to suppress postprandial hyperglycemia in T2DM.^11,12^ Acarbose, miglitol, and voglibose have been widely used in clinic as α-glucosidase inhibitors since the early 1990s, either alone or in combination with other antidiabetic drugs.^13^ These classic α-glucosidase inhibitors have low efficacy with high IC_50_ values against the enzyme and suffer from gastrointestinal system side effects such as diarrhea, flatulence, abdominal bloating, and discomfort.^14−17^ Consequently, the need for safer inhibitors with a high degree of specificity is critical.^18^

**Scheme 1:**
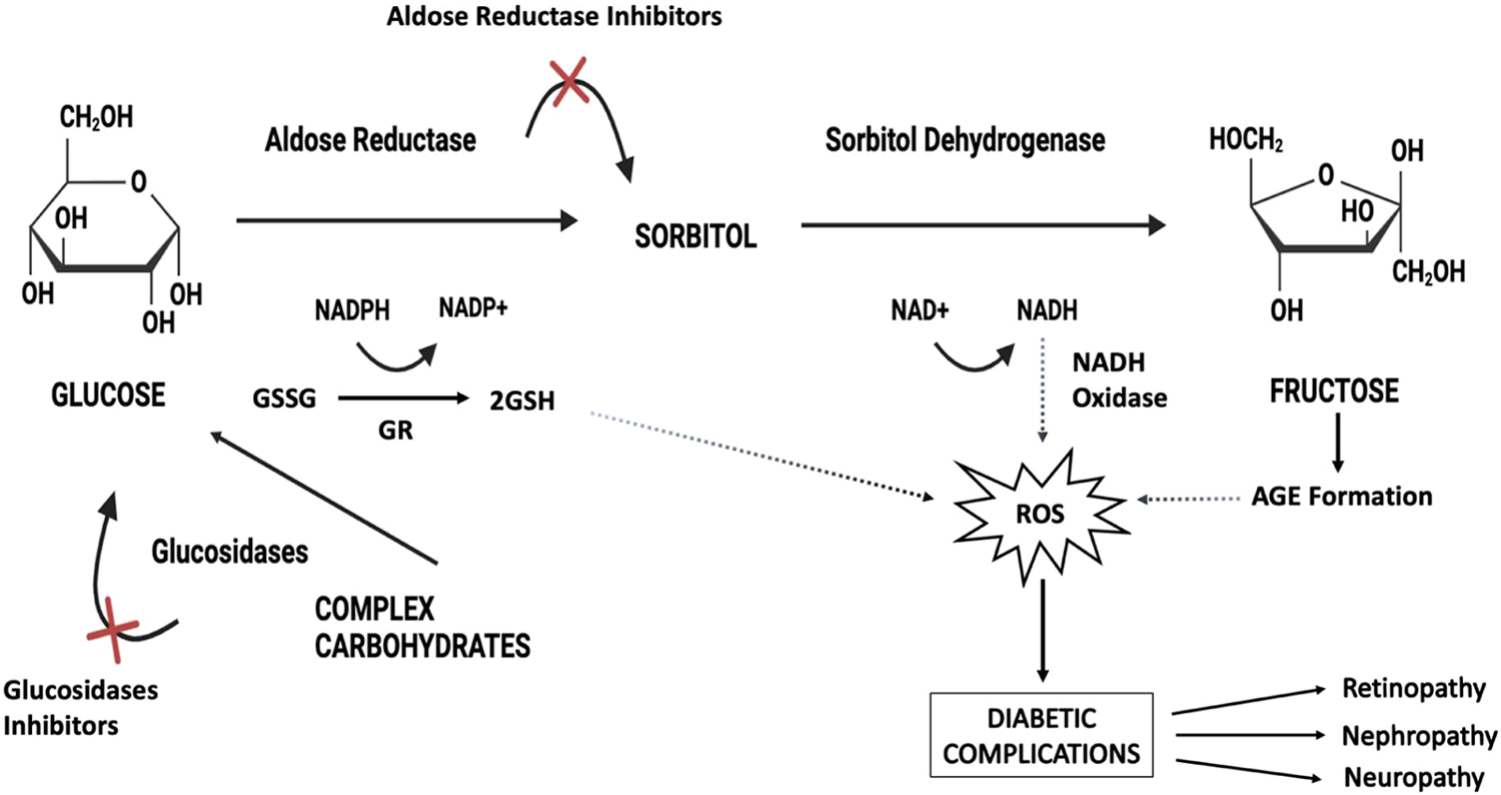
Figure Shows the Metabolism of Glucose via the Polyol Pathway and Its Contribution to Diabetic Complications Glucose is converted to sorbitol via the enzyme aldose reductase, consuming NADPH, which leads to a decrease in the antioxidant capacity of the cell. Sorbitol is oxidized to fructose via the enzyme sorbitol dehydrogenase, and NADH accumulates during this reaction. The accumulation of NADH leads to the formation of ROS via the enzyme NADH oxidase. The increase in ROS and the formation of AGEs by fructose contribute to diabetic complications such as retinopathy, nephropathy and neuropathy. Glucosidase inhibitors prevent the conversion of complex carbohydrates into glucose, while aldose reductase inhibitors help prevent complications by limiting the activity of polyol metabolism.^23,24^ (**NADPH**: Nicotinamide adenine dinucleotide phosphate, **NADH**: Nicotinamide adenine dinucleotide, **ROS**: Reactive oxygen species, **AGE**: Advanced glycation end products).

AR (EC: 1.1.1.21) is the first rate-limiting enzyme involved in the polyol pathway and plays a prominent role in explaining the pathogenesis of complications in patients with DM.^19^ AR catalyzes the reduction of excess glucose to sorbitol, converting nicotinamide adenine dinucleotide phosphate (NADPH) to NADP^+^.^20^ Consequently, the intracellular accumulation of highly polar sorbitol results in the formation of osmotic stress-inducing cellular damage in insulin-independent tissues such as lens, retina, kidney, and peripheral nerves. The inhibition of AR is a possible prevention or retardation of the onset and progression of these DM associated microvascular conditions.^21^ Aldose reductase, a key enzyme in the polyol pathway, reduces glucose to sorbitol in a nicotinamide adenine dinucleotide phosphate (NADPH)-dependent manner. This process leads to excessive accumulation of intracellular reactive oxygen species (ROS) in various tissues of diabetes mellitus (DM) including the neurons, eyes, heart, vasculature, and kidneys. This critical role of AR is not only limited to the pathogenesis of DM, but has also become an important target for understanding the mechanisms of many diseases and developing therapeutic strategies.^22^ Accordingly, the search for inhibitors of α-GLY and AR has the potential to provide effective therapeutic approaches for diabetes and related complications (Scheme 1).

Medicinal chemists are particularly interested in heterocyclic analogs due to their remarkable and exceptional chemical characteristics. 1,3,4-Thiadiazoles, a subclass of heterocyclic compounds, occupy a prime place in medicinal chemistry in recent years due to their wide range of pharmacological properties^25−31^ and their susceptibility to developing of new and easily functionalizable drug-like moieties. Additionally, there are locations in its structural unit, –N=C–S–, that can create powerful hydrogen bonds with the active hydrogen molecules in receptors. Therefore, 1,3,4-thiadiazole may be able to form a connection with a target protein to increase the parent molecule’s affinity.^32^ Besides, the synthetic compounds containing 1,3,4-thiadiazole moiety have been reported as α-glucosidase inhibitors.^33−39^

**Figure 1:**
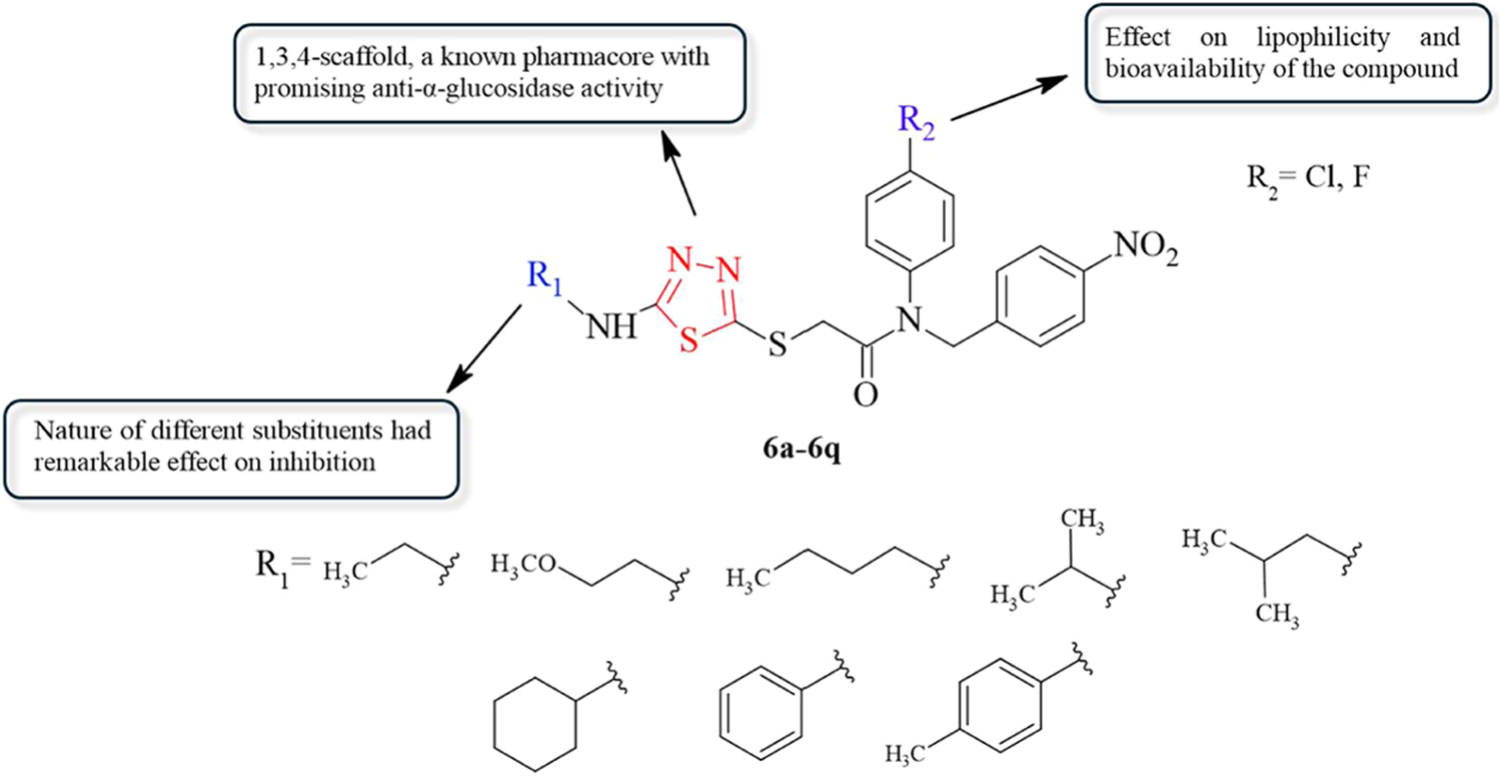
Structures of the designed compounds (**6a**–**6q**).

Thus, in this paper, we are reporting the design and synthesis and *in silico* studies of novel 1,3,4-thiadiazole derivatives in the search for new inhibitors of dual α-GLY and AR with potential antidiabetic activity (Figure 1). Molecular docking calculations were used to evaluate the activity of the synthesized molecules against α-GLY and AR proteins. ADME/T calculations were then performed to evaluate the effects and reactions of these molecules in the context of human metabolism. Additionally, we aimed to determine the *in vitro* cytotoxic effect of compounds.

## 2 Results and Discussion

### 2.1 Chemistry

**Scheme 2:**
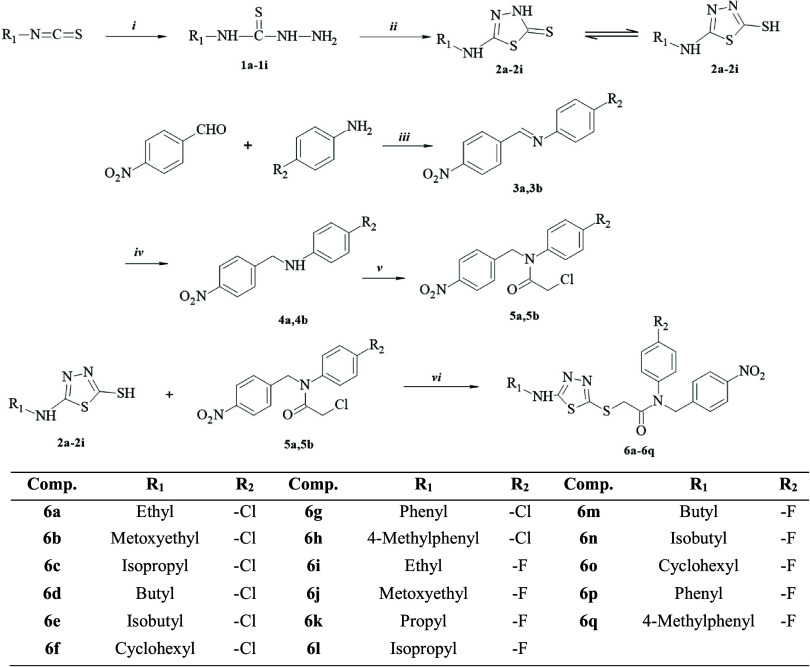
Synthetic Routes for Preparing Title Compounds (**6a**–**6q**) Reagents and conditions; ***i***: hydrazine hydrate, ethanol, rt; 4 h ***ii***: (1) carbon disulfide, potassium hydroxide, ethanol, reflux, 10 h (2) hydrochloric acid, pH 4–5; ***iii***: acetic acid, ethanol, reflux, 8 h; ***iv***: sodium borohydride, methanol, rt; 10 h; ***v***: chloroacetyl chloride, triethylamine, tetrahydrofuran, ice-bath, 5 h; ***vi:*** potassium hydroxide, acetone, rt, 8 h.

Scheme 2 shows the reaction steps involved in the synthesis of new thiadiazole derivatives. Initially, the compounds **1a**–**1i** were obtained by reacting substituted isothiocyanate with hydrazine hydrate in EtOH. Intermediates **1a**–**1i** were then thiadiazole ring-closed with carbon disulfide to obtain intermediates **2a**–**2i**. Then, the 4-nitrobenzaldehyde was condensed with the 4-substituted aniline derivative in refluxing ethanol and using a catalytic amount of glacial acetic acid to obtain Schiff’s bases derivatives **3a** and **3b**. In the next step, the resulting imine bond was reduced with sodium borohydride in methanol to obtain compounds **4a** and **4b**. Next, acetylated compounds **5a** and **5b** were afforded with the reaction of compounds **3a** and **3b**, and chloroacetyl chloride in the presence of triethylamine in the ice bath. The synthetic strategy has been developed by clubbing of the compounds **5a** and **5b** with 5-substitutedamino-1,3,4-thiadiazole-2(3H)-thiones (**2a**–**2i**) via sulfur linkage to furnish *N*-(4-substitutedphenyl)-2-[(5-substitutedamino-1,3,4-thiadiazol-2-yl)thio]-*N*-(4-nitrobenzyl)acetamide derivatives (**6a**–**6q**).

The structure of these compounds **6a**–**6q** was confirmed using spectroscopic methods. In the ^1^H NMR, the singlet signals of two CH_2_ groups (CO–CH_2_ and N–CH_2_) were observed at δ 3.91–4.05 and 4.99–5.02 ppm, respectively. In compounds with alkylamino groups attached to the thiadiazole ring, NH protons were observed in the range of δ 7.71–7.91 ppm, while in compounds with arylamino groups attached to the thiadiazole ring (**6g**, **6h**, **6p**, **6q**), NH protons were observed in the range of δ 10.26–10.30 ppm. Aromatic protons belonging to the phenyl ring were detected in the range of 6.99–8.16 ppm. In the proton spectra of the ethyl group in compounds **6a** and **6i**, CH_3_ protons were observed as triplets at 1.15 ppm, while −CH_2_ protons were observed as multiplets in the range of 3.21–3.30 ppm. While the OCH_3_ protons of the 2-methoxyethyl substituent, which is common in compounds **6b** and **6j**, were observed as singlet at 3.26 ppm, ethyl protons were detected as multiplet in the range of 3.41–3.48 ppm. Protons belonging to propyl and butyl groups in compounds **6c**, **6d**, **6e**, **6k**, **6l**, **6m**, and **6n** were detected in the range of 0.88–3.84 ppm. In compounds **6f** and **6o**, which have a cyclohexyl structure, protons belonging to the cyclohexyl structure were observed in the range of 1.14–1.96 ppm. The ^13^CNMR spectra of all of the derivatives showed carbon values in the predictable regions, while the HRMS analysis confirmed the mass with the calculated values of the target compounds.

### 2.2 Biological Activity

The present study investigates the inhibitory properties of the 1,3,4-thiadiazole derivatives (**6a**–**6q**) against AR and α-GLY enzymes. The study’s primary objective was to ascertain the potential efficacy of these derivatives and to provide recommendations for developing novel therapeutic agents for inhibiting diabetes-related enzymes. The findings and inhibition data of compounds **6a**–**6q** and references are provided in Table 1.

**Table 1:**
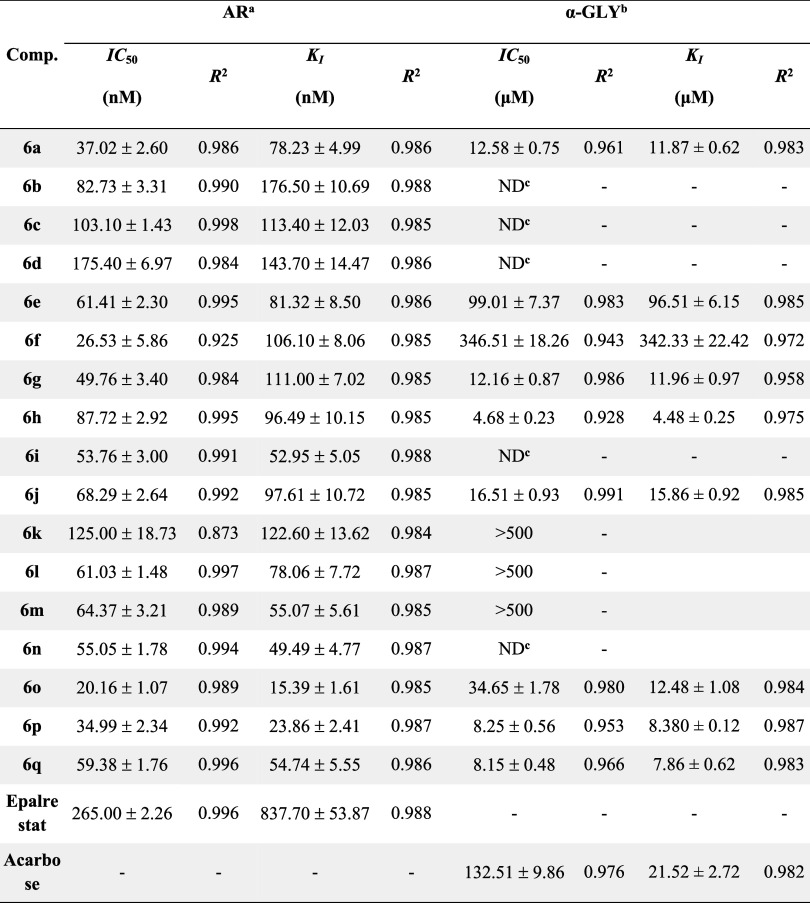
IC_50_ and *K*_I_ Values as Inhibitory Potential of Novel 1,3,4-thiadiazole Derivatives (**6a**–**6q**) Against AR and α-GLY

a Aldose reductase.

b α-Glycosidase.

c Not determined. Quantitative values of compounds with IC_50_ values of 500 and below are given in the Table.

The 1,3,4-thiadiazole derivatives (**6a**–**6q**) exhibited potent inhibitory activity on the diabetes-related enzymes AR and α-GLY at nanomolar and micromolar concentrations. The inhibitory capacity of these compounds against AR was evaluated by *K*_I_ values ranging from 15.39 ± 1.61 to 176.50 ± 10.69 nM and IC_50_ values ranging from 20.16 ± 1.07 to 175.40 ± 6.97 nM. All synthesized compounds showed a higher potential in terms of inhibitory activity on AR compared to the reference inhibitor epalrestat (IC_50_ for epalrestat: 265.00 ± 2.26 nM; *K*_I_: 837.70 ± 53.87 nM). Among the compounds examined, compounds **6o** and **6p** were found to be the most potent inhibitors for AR, with *K*_I_ values of 15.39 ± 1.61 and 23.86 ± 2.41 nM, respectively. In contrast, compound **6b**, although having a lower *K*_I_ value than the reference compound (837.70 ± 53.87 nM), showed a weaker inhibitory effect compared to the other derivatives. In the context of enzyme kinetics, *K*_I_ values provide important information about the affinity and selectivity of inhibitors toward the target enzyme. In this context, the results summarized in Table 1 revealed that compound **6o** had the highest selectivity on AR, whereas compound **6b** showed the lowest selectivity. The 1,3,4-thiadiazole derivatives synthesized within the scope of the study are attracting attention as potential therapeutic agents, especially by exhibiting superior inhibitory effects on AR enzyme compared to the reference inhibitor epalrestat.

The antidiabetic potential of the compounds was evaluated through kinetic studies, revealing inhibitory effects with IC_50_ values ranging from 4.68 ± 0.23 to >500 μM and *K*_I_ values ranging from 4.48 ± 0.25 to >500 μM for α-GLY. The compounds **6a**, **6g**, **6h**, **6j**, **6o**, **6p**, and **6q** showed significantly higher inhibitory activity toward α-GLY compared to the reference acarbose (IC_50_: 132.51 ± 9.86 μM; *K*_I_: 21.52 ± 2.72 μM). Among the tested compounds, compound **6h** emerged as the most potent inhibitor of α-GLY, with a *K*_I_ value of 4.48 ± 0.25 μM. In terms of enzyme kinetics, compound **6p** exhibited the highest selectivity for AR and α-GLY, while compounds **6b** and **6d** displayed the lowest selectivity for α-GLY and α-AMY as *K*_I_ values, as summarized in Table 1.

**Figure 2:**
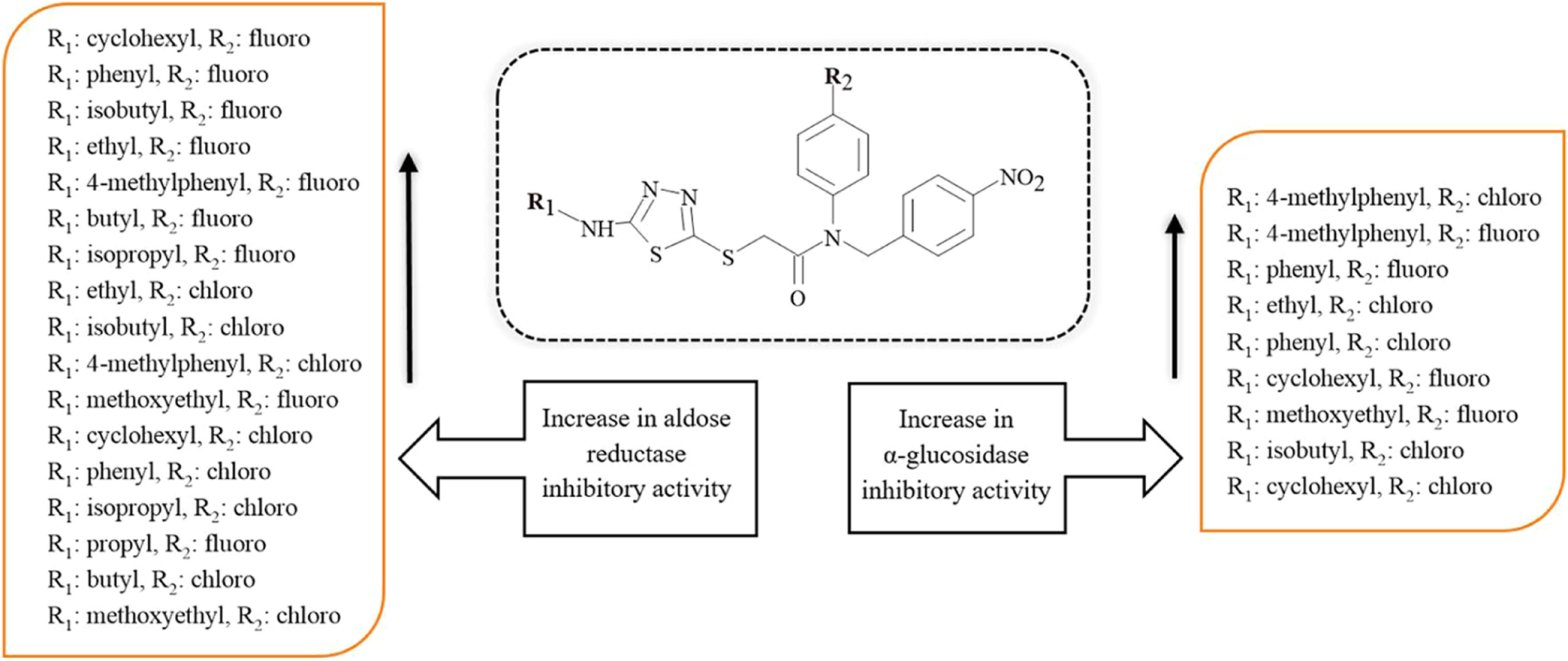
SAR study of compounds **6a**–**6q**.

The structure–activity relationship (SAR) study of compounds **6a**–**6q** is shown in Figure 2. Based on the SAR study, compound **6o** (R_1_: cyclohexyl, R_2_: fluoro) showed the best inhibitory activity in the case of AR inhibitory activity. The second potent compound was compound **6p** (R_1_: phenyl, R_2_: fluoro). It may be suggested that compounds bearing fluoro as R_2_ substituent generally showed higher inhibitory activity compared to compounds with chloro at the same position. On the other hand, the introduction of the isobutyl group as an R_1_ substituent, as in the case of compounds **6n** and **6e**, improved the inhibition effect in comparison to the presence of the *n*-butyl group, as in the case of compounds **6m** and **6d**. The substitution of 2-methoxyethyl and butyl groups as R_1_ substituents (compounds **6b** and **6d**) dramatically decreased the inhibition effect. On the other hand, compounds **6h** and **6q** with both 4-methylphenyl as R_1_ substituent, along with chloro and fluoro, respectively, as R_2_ substituent, were determined as the most potent α-GLY inhibitors. It was also shown that the presence of an aromatic group (*p*-methylphenyl and/or phenyl) predominantly increases α-GLY inhibitory activity more than an aliphatic group. Besides, 2-methoxyethyl, isopropyl, butyl, and propyl groups as R_1_ substituents, as in the case of compounds **6b**, **6c**, **6d**, **6i**, **6k**, **6l**, **6m**, and **6n**, dramatically decreased α-GLY activity.

In a study, indole-based thiazole derivatives were synthesized, and their inhibition effect on α-GLY was investigated for potential antidiabetic effect. They reported that these derivatives showed α-GLY inhibition activity with IC_50_ in the range of 111.8 ± 0.9001 and 666.5 ± 1.0111 μM.^40^ In another study, benzimidazole-based thiazole derivatives were synthesized, and their inhibitory potential against α-GLY enzyme was determined with IC_50_ ranging from 2.71 ± 0.10 to 42.31 ± 0.70 μM.^41^ Moreover, a group of researchers synthesized thiazolidinone-based benzothiazole derivatives and investigated their inhibition potential against the α-GLY enzyme. These derivatives showed inhibition potential with IC_50_ values in the range of 3.20 ± 0.05 to 39.40 ± 0.80 μM.^42^ In a study, thiazoline-based compounds were synthesized, and it was determined that the most effective compound among these derivatives showed inhibition effect against AR enzyme with an IC_50_ value of 3.14 ± 0.02 μM.^43^ In another study, a series of 3-substituted 4-oxo-2-thioxo-1,3-thiazolidines were designed, and it was reported that the most effective compounds showed an inhibition effect against the AR with IC_50_ values of 1.22 ± 0.67 and 2.34 ± 0.78 μM.^44^ Moreover, a group of researchers synthesized quinazolinone-based 2,4-thiazolidinedione-3-acetic acid derivatives and investigated their inhibitory potential against AR. They found that the most effective compound showed an inhibitory effect with an IC_50_ value of 2.56 nM.^45^ In this context, the inhibitory potential of many of the compounds synthesized in this study against α-GLY and AR enzymes is significantly stronger than the best available results in the literature.

### 2.3 Molecular Docking Study

In general, molecular docking calculations are performed to support experimental activities and identify molecules’ active sites. Molecular modeling is an important method for studying the interactions of molecules with proteins through molecular docking calculations.^46^ This method determines the activity of molecules against proteins and the interaction between them, and as this interaction increases, the activity of the molecules increases. Many parameters were calculated as a result of the calculations, and each parameter gave information about the different properties of the molecules.^47^ When these parameters are analyzed, the first parameter that determines the activity of the molecules is the docking score parameter.

**Figure 3:**
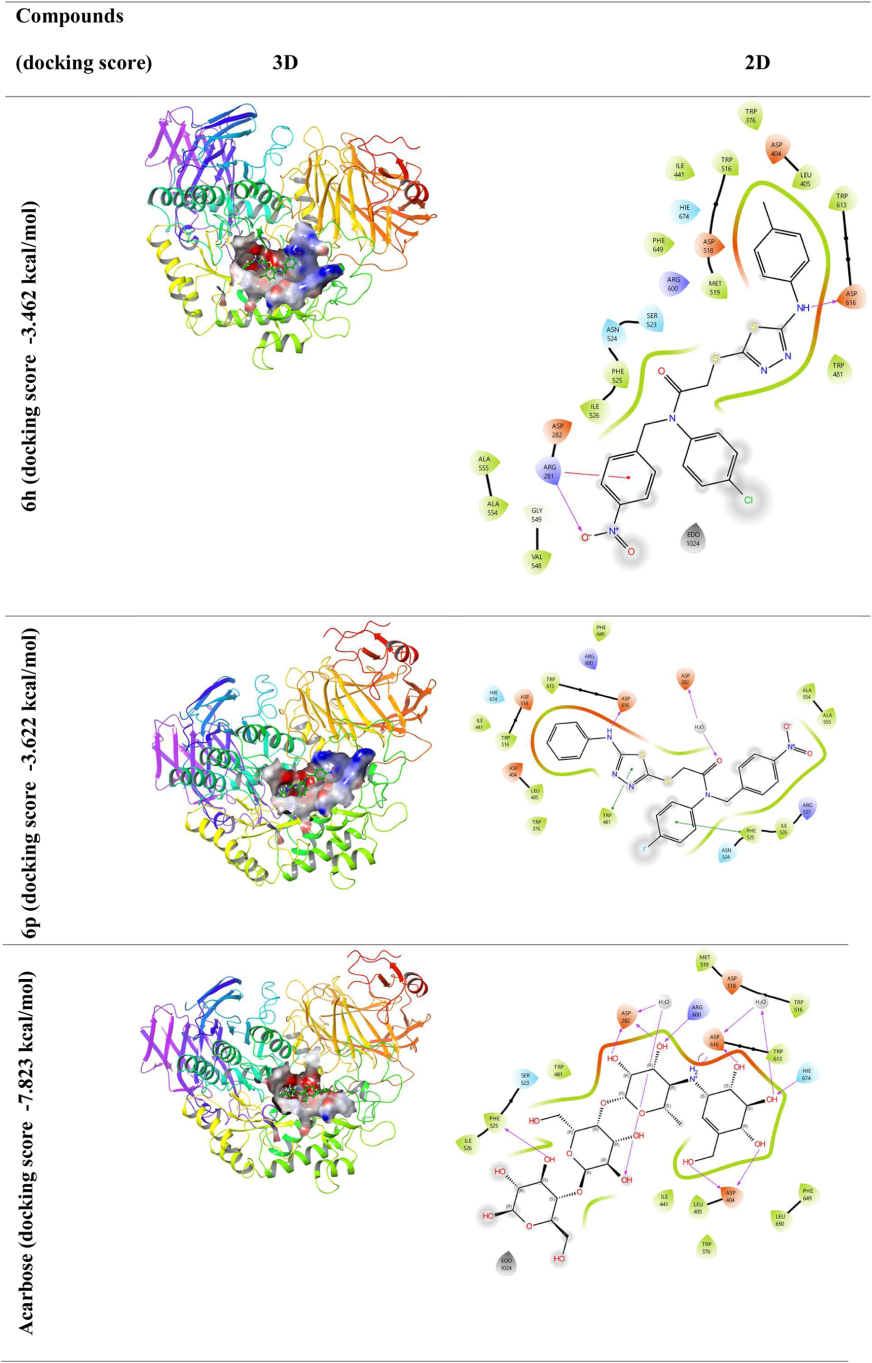
Protein–ligand interaction (3D and 2D). α-GLY, represented by 5NN8, was subjected to molecular docking studies with compound **6h**, **6p**, and acarbose.

**Figure 4:**
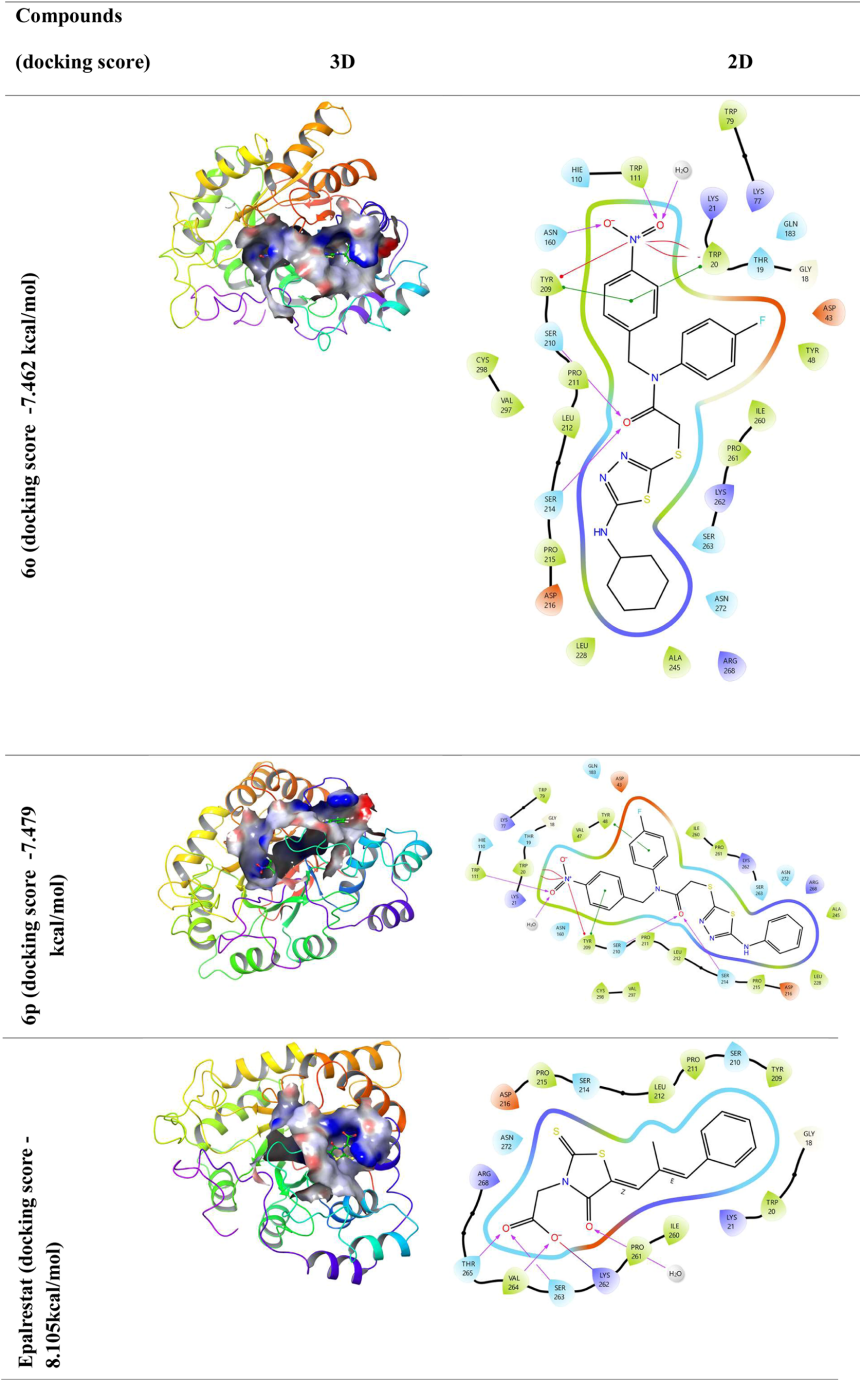
Protein–ligand interaction (3D and 2D). AR, represented by 4JIR, was subjected to molecular docking studies with compound **6o**, **6p** and epalrestat.

According to the docking studies, compounds **6o** and **6p** have a higher binding affinity (−7.462 and −7. 479 kcal/mol, respectively) in their interaction with the 4JIR receptor, and the binding affinity of epalrestat used as a positive control (−8.105 kcal/mol). The binding states of compounds **6h** and **6p**, although low compared to acarbose, exhibited strong interactions with the active site. When the *in vitro* and *in silico* findings of the compounds in Table 1 and Figures 3 and 4 are evaluated, it is understood that the compounds have a strong tendency to bind to active sites. The lower the negative docking score value, the more effective the binding is. Compounds **6o** and **6p** seem to have a very close affinity with epalrestat. As the interaction between molecules and proteins increases, the activity of the molecules also increases.^48^

Compound **6h** interacts with the target protein 5NN8 by forming two hydrogen bonds with the backbone residues Asp616 and Arg281, while compound **6p** establishes two hydrogen bonds with Asp282 and Asp516, and acarbose forms six hydrogen bonds with Phe525, Asp282, Arg600, Asp616, His671, and Asp404.

Compound **6o** forms hydrogen bonds with the backbone residues Ser214, Ser210, Asn160, and Trp111 of the target protein 4JIR, while compound **6p** interacts with the same protein via hydrogen bonds involving Trp111, Tyr281, Ser210, and Ser214, and epalrestat establishes hydrogen bonds with Thr265, Val264, Ser263, and Lys264 residues.

*In vitro* and *in silico* findings also suggest that the presence of different groups in the synthesized compounds may enhance their activity by modifying their physicochemical properties and pharmacokinetic parameters to increase their bioavailability and metabolic stability as well as their binding affinity to receptors.

### 2.4 ADME/T Analysis

ADME/T analysis (absorption, distribution, metabolism, excretion, and toxicity) was performed to examine the effects and responses of these studied molecules in human metabolism. With this analysis, the absorption of the molecules by human metabolism, their distribution in human metabolism, their excretion from metabolism, and finally, their toxicity values in metabolism were calculated. Many parameters that analyze the chemical properties of molecules are calculated, such as mol_MW (molar mass of molecules), Molecular Weight (MW), Volume (molecular volume), Log P (The degree of lipophilicity of the molecule), TPSA (Total Polar Surface Area, Refers to the polar surface area of the molecule, affects bioavailability), nRot (Number of rotationally free bonds), LogS (Degree of water solubility), nHA and nHD (Refers to the number of atoms that accept and give hydrogen bonds). The physicochemical and ADME properties of the synthesized compounds (**6h**, **6o**, and **6p**) and acarbose and epalrestat are given in Table 2.

**Table 2:**
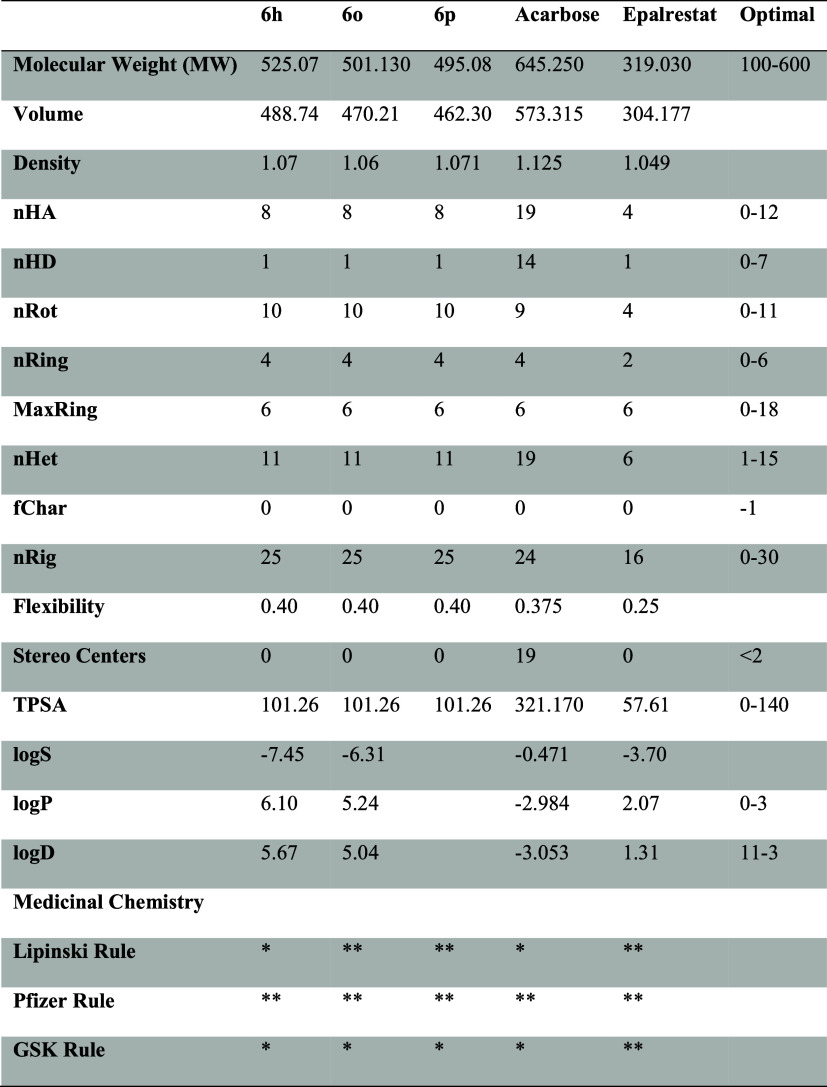
Physicochemical and ADME Properties of Synthesized Compounds (**6h**, **6o**, and **6p**), Acarbose and Epalrestat^a^

a * Rejected; ** Accepted.

The analyzed cytotoxicity data for compounds **6h**, **6o**, and **6p** show molecular weights of 525.07, 501.130, and 495.08 g/mol, respectively, with total polar surface area (TPSA) of 101.26 Å, nHD 1, nRing 4.00, nRot 10 for all compounds. Orally active drugs transported transcellularly should not exceed a PSA of approximately 120 Å. A total polar surface ranging below 120 Å indicates good oral absorption and brain penetration.^49−51^ Compounds **6h** (Log P: 6.10) and **6o** (Log P: 5.24) exhibit high lipophilic properties. High Log P is usually associated with low aqueous solubility, which may negatively affect bioavailability.^52^ This is supported by the fact that the logS value of **6h** (−7.45) is particularly low, indicating poor aqueous solubility of the compound. Compared to the reference compounds, acarbose’s Log P value (−2.98) and TPSA value (321.17 Å^2^) show better water solubility, while epalrestat (Log P: 2.07) has a more balanced lipophilicity profile. High Log P values of the studied compounds mean lower aqueous solubility and potentially lower bioavailability.^53^ It is known that the lipophilicity of compounds with Log P values above 5 increases, which may pose some difficulties in terms of bioavailability. However, such deviations do not always negatively affect the drug development process.^54^ Especially in studies on enzyme inhibition, compounds with high potential for interaction with the active site of the targeted protein can be preferred. In addition, ADME/T analyses show that the compounds exhibit acceptable pharmacokinetic properties.^55,56^ Therefore, we can say that high Log P values do not completely exclude the biopharmaceutical suitability of the compounds.

In the results, an appropriate number of rotatable bonds, H-bond donors, H-bond acceptors, and values indicating that most of the derivatives follow Lipinski’s rule of 5 were found. Lipinski’s rule of 5 is a quantitative approach to the qualitative prediction of oral absorption.

The topological polar surface area (TPSA) is associated with the hydrogen bonding of a molecule and is a reliable predictor of bioavailability. Considering the drug-like parameters predicted by ADME analysis that compounds (**6h**, **6o**, and **6p**) have TPSA in the optimum range of 101.26 Å, compounds **6h**, **6o**, and **6p** can be said to exhibit drug-like behavior.

**Figure 5:**
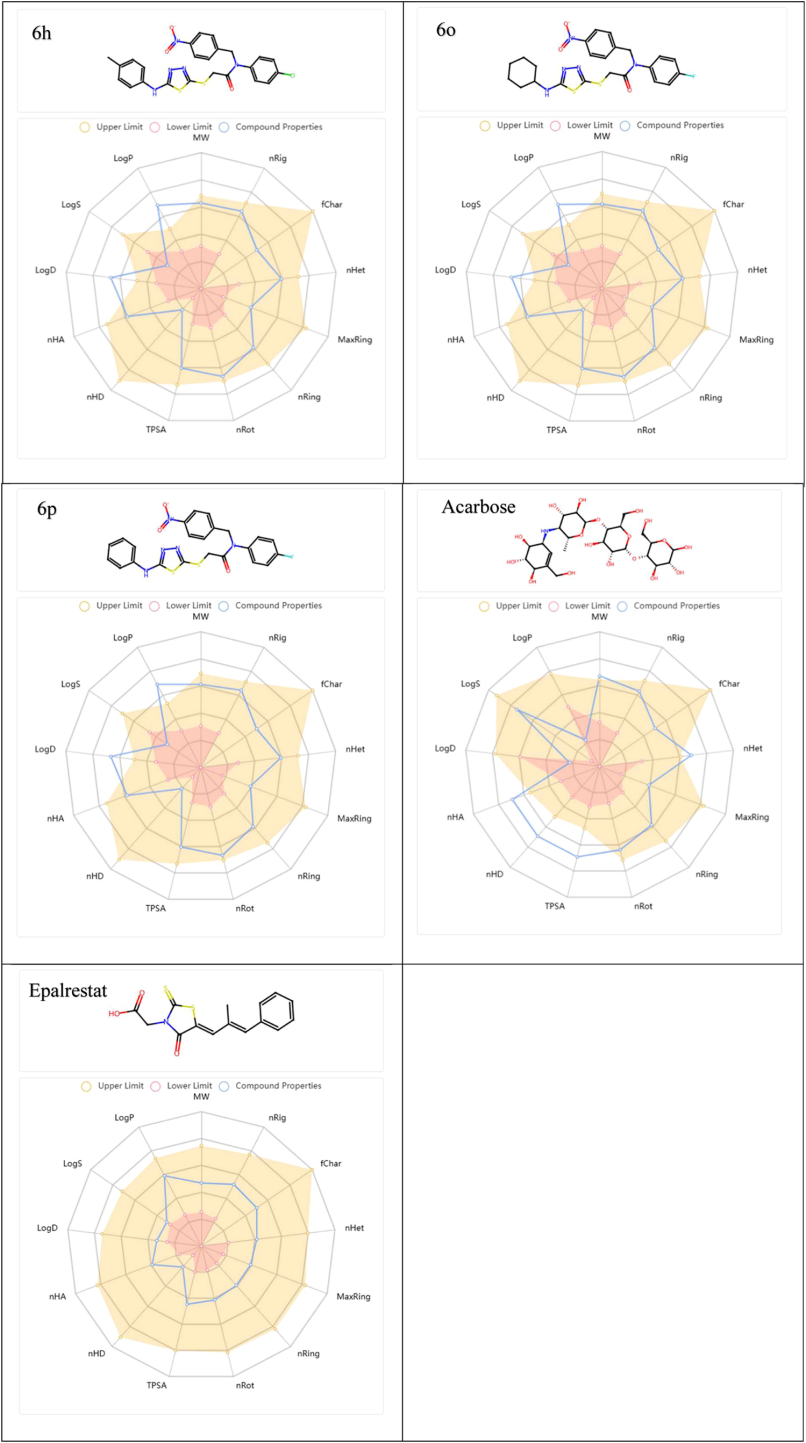
Radar graph showing the chemical structure and physicochemical properties of compounds (**6h**, **6o**, and **6p**) and acarbose, epalrestat.

The chemical structure of **6h**, **6o**, and **6p**, acarbose and epalrestat, and the physicochemical properties of this molecule are illustrated by a radar plot (spider plot). Molecular properties (e.g., Log P, LogS, nHD, TPSA) are expressed as a circle around it. The red line indicates the lower limit of the molecule’s properties, the yellow area indicates the range of upper and lower limits, and the blue line indicates the compliance of the molecule under investigation with these properties (Figure 5).

### 2.5 Cytotoxicity Test

**Figure 6:**
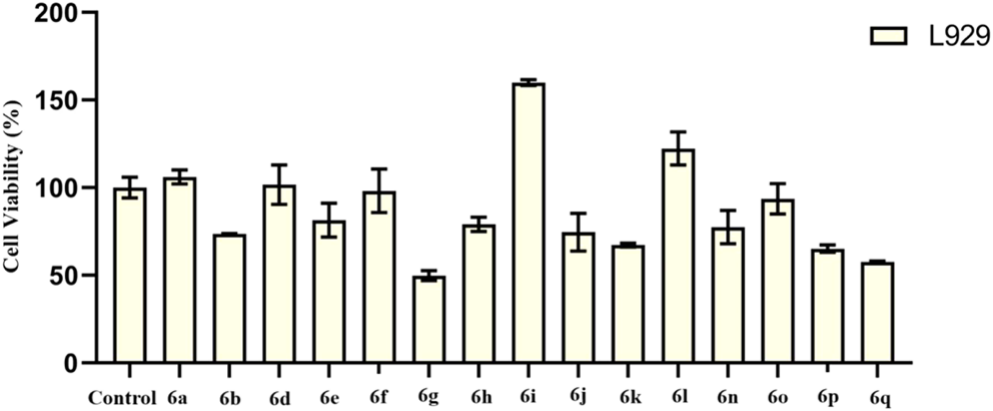
Cell viability of the synthesized compounds (**6a**–**6q**) at maximum dose (100 μM) for 24 h.

Cell viability was revealed by measuring the 96-well plate with a spectrophotometer after 24 h of incubation after the synthesized compounds were given. The IC_50_ values of all of the compounds except compound **6k** were found to be higher than 100 μM. The IC_50_ value of compound **6g** was determined as 99.53 ± 5.66 μM. Figure 6 shows the cell viability rates when the maximum doses of the compounds **6a**–**6q** were given (100 μM).

### 2.6 Ames II Test

*In vitro* genotoxicity tests, which allow the analysis of very small amounts of compounds with high efficiency in the drug development process, provide important contributions to plan the process by determining the toxicity of compounds at early stages.^70^ Due to a mutation in the histidine (His) operon of *Salmonella typhimurium*, the bacteria cannot produce histidine. This causes bacteria to be unable to multiply without histidine support. When a mutagenic event occurs, a base pair/frameshift mutation in the histidine gene can cause a reversal. As a result, bacteria can multiply without histidine. The mutagenic potential of a chemical can be assessed by determining whether it makes this reversal. The use of medium without histidine allows only the mutated bacteria to survive and multiply.^71^*S. typhimurium* TA98 strain is used to detect mutagens causing frameshift mutations, while TA mix strains are used to detect mutagens causing base pair mutations. S9 rat liver microsome enzyme fractions are used to mimic mammalian metabolism. This step is important for the evaluation of the mutagenicity of metabolites formed by biotransformation of the chemical.^72^

**Figure 7:**
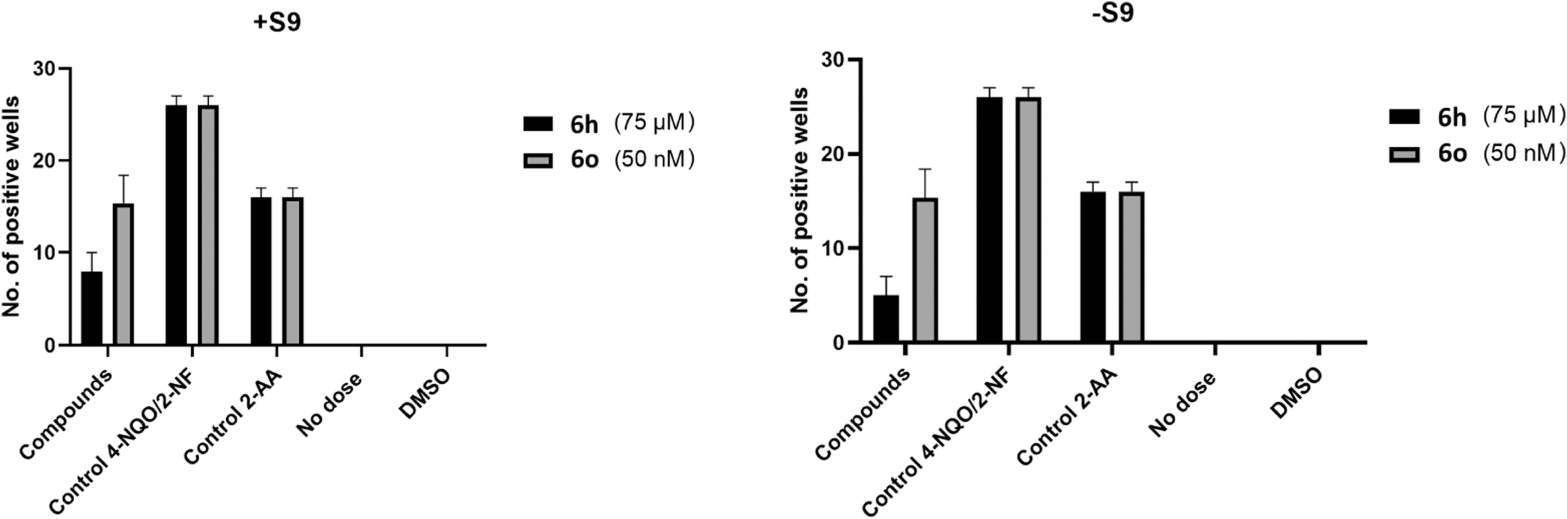
Average number of positive wells at effective concentrations.

To evaluate the mutagenicity of the test substances, *S. typhimurium* TA 98 and TA mix bacterial strains were studied in the presence and absence of the S9 enzyme fraction. The results were evaluated according to the kit procedure. At the end of the assay, averages of the number of positive (yellow) wells by dose were calculated from triplicate replicates (Table 3, Figure 7). According to the average of the results, mutagenicity was detected both in the presence and absence of S9 in the TA mix strain at a concentration of 75 μM of compound **6h** and 50 nM of compound **6o**. The fact that mutagenicity was observed only in the TA mix culture indicates that the substances cause base pair mutation at the concentrations indicated.

**Table 3:** Average Number of the Positive Wells

		**mutagenicity**			
**compounds**		**TA 98**		**TA Mix**	
		**S9+**	**S9**–	**S9+**	**S9**–
**comp. 6h**	DMSO	0	0	0	0
	75 μM	0	0	8	5
	37.5 μM	0	0	0	0
	18.75 μM	0	0	0	0
	9.4 μM	0	0	0	0
	4.7 μM	0	0	0	0
	control 4-NQO/2-NF	26	26	26	26
	control 2-AA	16	16	16	16
	no dose	0	0	0	0
**comp. 6o**	DMSO	0	0	0	0
	50 nM	0	0	15.3	15.3
	25 nM	0	0	0	0
	12.5 nM	0	0	0	0
	6.25 nM	0	0	0	0
	3.125 nM	0	0	0	0
	control 4-NQO/2-NF	26	26	26	26
	control 2-AA	16	16	16	16
	no dose	0	0	0	0

SD values of the results were also calculated (Table 4). According to the kit procedure, the standard deviation of positive (yellow) wells per concentration is equal to the standard deviation values for the average number of positive wells. Furthermore, the efficiency according to fold induction means the increase in the average number of positive wells above the average solvent (DMSO) control. If the SD value for the mean number of positive wells for solvent control is less than 1.0, it is taken as 1.0 for correct calculation. Accordingly, interpreting the SD values, it was determined that compound **6h** (75 μM) and compound **6o** (50 nM) can cause base pair mutation in TA mix strain both in the presence and absence of S9.

**Table 4:** SD Values of the Positive Wells

		**mutagenicity**			
**compounds**		**TA 98**		**TA Mix**	
		**S9+**	**S9**–	**S9+**	**S9**–
**comp. 6h**	DMSO	1	1	1	1
	75 μM	1	1	2	2
	37.5 μM	1	1	1	1
	18.75 μM	1	1	1	1
	9.4 μM	1	1	1	1
	4.7 μM	1	1	1	1
	control 4-NQO/2-NF	1	1	1	1
	control 2-AA	1	1	1	1
	no dose	1	1	1	1
**comp. 6o**	DMSO	1	1	1	1
	50 nM	1	1	3.055	3.055
	25 nM	1	1	1	1
	12.5 nM	1	1	1	1
	6.25 nM	1	1	1	1
	3.125 nM	1	1	1	1
	control 4-NQO/2-NF	1	1	1	1
	control 2-AA	1	1	1	1
	no dose	1	1	1	1

According to the results of the study, the fact that these test substances have mutagenic potential only at high concentrations indicates that they can be used effectively and safely as a result of more comprehensive studies.

## 3 Materials and Methods

### 3.1 Chemistry

All compounds were purchased from Merck Chemicals (Merck KGaA, Darmstadt, Germany) and Sigma-Aldrich (Sigma-Aldrich, St. Louis, MO). Electrothermal 9100 digital melting point apparatus (Electrothermal, Essex, UK) was used to record all melting points (m.p.). Thin-layer chromatography (TLC) with Silica Gel 60 F254 TLC plates (Merck) was employed to monitor each reaction. Spectroscopic data of the synthesized compounds were registered with the following instruments: ^1^H NMR, Bruker DPX 300 NMR spectrometer (Billerica, MA), ^13^C NMR, Bruker DPX 75 NMR spectrometer (Bruker) in DMSO-*d*_6_, using tetramethylsilane (TMS) as the internal standard; HRMS, Shimadzu LC/MS ITTOF system (Shimadzu).

#### 3.1.1 Synthesis of 4-Substitutedthiosemicarbazides (**1a**–**1i**)

To a solution of substituted isothiocyanate (20 mmol) in ethanol (50 mL) was added hydrazine hydrate (40 mmol), and the mixture was stirred at room temperature for 4 h. The final products were obtained through recrystallization using ethanol.

#### 3.1.2 Synthesis of 5-Substitutedamino-1,3,4-thiadiazole-2(3H)-thione (**2a**–**2i**)

A mixture containing compounds **1a**–**1i** (23 mmol) and carbon disulfide (27 mmol, 1.6 mL) was refluxed in ethanol in the presence of K_2_CO_3_ (27 mmol, 3.7 g) for 10 h. The progress of the reaction was monitored by TLC. The solution was poured into ice–water and acidified with a hydrochloric acid to a pH of 4–5. The obtained product was recrystallized from ethanol.

#### 3.1.3 Synthesis of 4-Substituted-*N*-(4-nitrobenzylidene)aniline (**3a,3b**)

A catalytic quantity of glacial acetic acid (0.5 mL) was added to the solution of 4-nitrobenzaldehyde (40 mmol, 6 g) and 4-substituted aniline (40 mmol) in ethanol (100 mL). The mixture was refluxed for 8 h. After TLC monitoring, the mixture was poured onto crushed ice, and the separated solid was filtered and recrystallized from ethanol.

#### 3.1.4 Synthesis of 4-Substituted-*N*-(4-nitrobenzyl)aniline (**4a,4b**)

Sodium borohydride was added to the methanolic (100 mL) solution of compound 3*a*/3b (20 mmol) in 4 sections (4 × 0.5 g) at 15 min intervals. The reaction mixture was stirred at room temperature for 1 h. After this, the solvent was removed by evaporation, the resulting crude product was then dried, cleaned washed several times with water, and recrystallized from ethanol.

#### 3.1.5 Synthesis of 2-Chloro-*N*-(4-substitutedphenyl)-*N*-(4-nitrobenzyl)acetamide (**5a,5b**)

A solution of compound 5*a*/5b (0.02 mol) in tetrahydrofuran (80 mL) was cooled to 0–5 °C. After addition of triethylamine (0.024 mol, 3.35 mL) to this, chloroacetyl chloride (0.024 mol, 1.91 mL) was added to the mixture in a dropwise manner. Following the end of dripping, the mixture was stirred at room temperature for 1 h postaddition. After evaporation of tetrahydrofuran, the remaining solid was rinsed with water, dried and recrystallized from ethanol.

#### 3.1.6 Synthesis of *N*-(4-substitutedphenyl)-2-[(5-substitutedamino-1,3,4-thiadiazol-2-yl)thio]-*N*-(4-nitrobenzyl)acetamide derivatives (**6a**–**6q**)

The intermediates 2-chloroacetamides (5a, 5b) (4 mmol), 2-mercaptothiadiazoles (2a-2i) (4 mmol) and also K_2_CO_3_ (5 mmol, 0.66 g) were dissolved in acetone (40 mL) and stirred at room temperature for 8 h. Target pure compounds (6a-6q) were obtained by removing the solvent under reduced pressure and further crystallization from ethanol. The target pure compounds (6a-6q) were obtained by removal of the solvent under reduced pressure followed by crystallization from ethanol.

##### 3.1.6.1 *N*-(4-Chlororophenyl)-2-[(5-(ethylamino)-1,3,4-thiadiazol-2-yl)thio]-*N*-(4-nitrobenzyl) Acetamide (**6a**)

Yield 76%. M.p.: 110.7 °C.^1^H NMR (300 MHz, DMSO-*d*_6_, ppm) δ 1.15 (3H, t, *J* = 7.2 Hz, CH_2_–CH_3_), 3.21–3.30 (2H, m, CH_2_-CH_3_), 3.93 (2H, s, CO–CH_2_), 5.00 (2H, s, N–CH_2_), 7.35 (2H, t, *J* = 8.6 Hz, Ar–H), 7.46–7.54 (4H, m, Ar–H), 7.79 (1H, t, *J* = 5.2 Hz, NH), 8.15 (2H, d, *J* = 8.8 Hz, Ar–H). ^13^C NMR (75 MHz, DMSO-*d*_6_, ppm) δ 14.66, 38.18, 39.86, 52.52, 123.97, 129.43, 130.21, 130.39, 133.35, 140.48, 145.34, 147.13, 149.15, 167.41, 169.85. HRMS (*m*/*z*): [M + H]^+^ calcd for C_19_H_18_ClN_5_O_3_S_2_: 464.0612; found 464.0591.

##### 3.1.6.2 *N*-(4-Chlororophenyl)-2-[(5-((2-methoxyethyl)amino)-1,3,4-thiadiazol-2-yl)thio]-*N*-(4-nitrobenzyl)acetamide (**6b**)

Yield 69%. Mp 80.5 °C.^1^H NMR (300 MHz, DMSO-*d*_6_, ppm) δ 3.26 (3H, s, OCH_3_), 3.41–3.48 (4H, m, CH_2_), 3.93 (2H, s, CO–CH_2_), 5.01 (2H, s, N–CH_2_), 7.34–7.38 (2H, m, Ar–H), 7.46–7.62 (4H, m, Ar–H), 7.88–7.91 (1H, m, NH), 8.07–8.13 (2H, m, Ar–H). ^13^C NMR (75 MHz, DMSO-*d*_6_, ppm) δ 38.11, 44.43, 52.53, 58.41, 70.40, 121.12, 123.97, 129.20, 129.43, 130.21, 130.39, 133.36, 138.21, 140.48, 145.34, 147.14, 149.56, 167.41, 169.81. HRMS (*m*/*z*): [M + H]^+^ calcd for C_20_H_20_ClN_5_O_4_S_2_: 494.0718; found 494.0700.

##### 3.1.6.3 *N*-(4-Chlororophenyl)-2-[(5-(isopropylamino)-1,3,4-thiadiazol-2-yl)thio]-*N*-(4-nitrobenzyl) acetamide (**6c**)

Yield 71%. M. p.: 136.1 °C. ^1^H NMR (300 MHz, DMSO-*d*_6_, ppm) δ 1.17 (6H, d, *J* = 6.7 Hz, 2CH_3_), 3.71–3.82 (1H, m, CH), 3.92 (2H, s, CO–CH_2_), 5.01 (2H, s, N–CH_2_), 7.35 (2H, d, *J* = 8.6 Hz, Ar–H), 7.46–7.54 (4H, m, Ar–H), 7.72 (1H, d, *J* = 7.1 Hz, NH), 8.15 (2H, d, *J* = 8.9 Hz, Ar–H). ^13^C NMR (75 MHz, DMSO-*d*_6_, ppm) δ 22.56, 38.13, 46.99, 52.53, 121.12, 123.96, 129.20, 129.43, 130.20, 130.39, 133.35, 138.20, 140.49, 145.35, 147.13, 149.00, 167.42, 169.04. HRMS (*m*/*z*): [M + H]^+^ calcd for C_20_H_20_ClN_5_O_3_S_2_: 478.0769; found 478.0752.

##### 3.1.6.4 *N*-(4-Chlororophenyl)-2-[(5-(butylamino)-1,3,4-thiadiazol-2-yl)thio]-*N*-(4-nitrobenzyl) acetamide (**6d**)

Yield 67%. M. p.: 122.3 °C. ^1^H NMR (300 MHz, DMSO-*d*_6_, ppm) δ 0.88 (3H, t, *J* = 7.4 Hz, CH_3_), 1.27–1.39 (2H, m, CH_2_), 1.48–1.57 (2H, m, CH_2_), 3.23 (2H, q, *J* = 6.2 Hz, CH_2_), 3.92 (2H, s, CO–CH_2_), 5.00 (2H, s, N–CH_2_), 7.35 (2H, d, *J* = 8.6 Hz, Ar–H), 7.46–7.54 (4H, m, Ar–H), 7.79 (1H, t, *J* = 5.4 Hz, NH), 8.15 (2H, d, *J* = 8.8 Hz, Ar–H). ^13^C NMR (75 MHz, DMSO-*d*_6_, ppm) δ 14.08, 20.00, 31.00, 38.13, 44.73, 52.53, 121.12, 123.96, 129.20, 129.43, 130.20, 130.38, 133.34, 138.20, 140.50, 145.35, 147.14, 149.01, 167.42, 170.03. HRMS (*m*/*z*): [M + H]^+^ calcd for C_21_H_22_ClN_5_O_3_S_2_: 492.0925; found 492.0912.

##### 3.1.6.5 *N*-(4-Chlororophenyl)-2-[(5-(isobutylamino)-1,3,4-thiadiazol-2-yl)thio]-*N*-(4-nitrobenzyl) Acetamide (**6e**)

Yield 73%. M.p.: 127.6 °C. ^1^H NMR (300 MHz, DMSO-*d*_6_, ppm) δ 0.89 (6H, d, *J* = 6.7 Hz, 2CH_3_), 1.80–1.93 (1H, m, CH), 3.06 (2H, d, *J* = 6.5 Hz, Ar–H), 3.93 (2H, s, CO–CH_2_), 5.00 (2H, s, N–CH_2_), 7.35 (2H, d, *J* = 8.6 Hz, Ar–H), 7.46 (2H, d, *J* = 8.6 Hz, Ar–H), 7.53 (2H, d, *J* = 8.6 Hz, Ar–H), 7.85 (1H, t, *J* = 5.6 Hz, NH), 8.15 (2H, d, *J* = 8.7 Hz, Ar–H). ^13^C NMR (75 MHz, DMSO-*d*_6_, ppm) δ 20.50, 27.95, 38.08, 52.55, 52.67, 123.95, 129.41, 130.19, 130.37, 133.35, 140.51, 145.35, 147.12, 148.98, 167.43, 170.19. HRMS (*m*/*z*): [M + H]^+^ calcd for C_21_H_22_ClN_5_O_3_S_2_: 492.0925; found 492.0901.

##### 3.1.6.6 *N*-(4-Chlororophenyl)-2-[(5-(cyclohexylamino)-1,3,4-thiadiazol-2-yl)thio]-*N*-(4-nitrobenzyl) Acetamide (**6f**)

Yield 70%. M. p.: 157.4 °C.^1^H NMR (300 MHz, DMSO-*d*_6_, ppm) δ 1.15–1.32 (5H, m, CH_2_), 1.53–1.70 (3H, m, CH_2_), 1.92–1.96 (2H, m, CH_2_), 1.43–1.50 (1H, m, CH), 3.92 (2H, s, CO–CH_2_), 5.00 (2H, s, N–CH_2_), 7.55 (2H, d, *J* = 8.7 Hz, Ar–H), 7.47 (2H, d, *J* = 8.5 Hz, Ar–H), 7.53 (2H, d, *J* = 8.5 Hz, Ar–H), 7.75 (1H, d, *J* = 7.1 Hz, NH), 8.15 (2H, d, *J* = 8.8 Hz, Ar–H). ^13^C NMR (75 MHz, DMSO-*d*_6_, ppm) δ 24.69, 25.68, 32.47, 38.05, 52.53, 53.91, 123.95, 129.41, 130.20, 130.37, 133.34, 140.53, 145.37, 147.13, 148.88, 167.42, 169.02. HRMS (*m*/*z*): [M + H]^+^ calcd for C_23_H_24_ClN_5_O_3_S_2_: 518.1082; found 518.1066.

##### 3.1.6.7 *N*-(4-Chlororophenyl)-2-[(5-(phenylamino)-1,3,4-thiadiazol-2-yl)thio]-*N*-(4-nitrobenzyl) Acetamide (**6g**)

Yield 62%. M. p.: 151.7 °C.^1^H NMR (300 MHz, DMSO-*d*_6_, ppm) δ 4.05 (2H, s, CO–CH_2_), 5.02 (2H, s, N–CH_2_), 7.00 (1H, t, *J* = 7.3 Hz, Ar–H), 7.32–7.60 (9H, m, Ar–H), 8.16 (2H, d, *J* = 8.6 Hz, Ar–H), 10.39 (1H, s, NH). ^13^C NMR (75 MHz, DMSO-*d*_6_, ppm) δ 37.93, 52.54, 117.81, 121.14, 122.44, 123.97, 129.47, 129.57, 130.26, 130.44, 133.42, 140.47, 140.83, 145.35, 147.16, 152.56, 165.20, 167.27. HRMS (*m*/*z*): [M + H]^+^ calcd for C_23_H_18_ClN_5_O_3_S_2_: 512.0612; found 512.0596.

##### 3.1.6.8 *N*-(4-Chlororophenyl)-2-[(5-(4-methylphenylamino)-1,3,4-thiadiazol-2-yl)thio]-*N*-(4-nitrobenzyl) Acetamide (**6h**)

Yield 62%. M.p.: 140.2 °C.^1^H NMR (300 MHz, DMSO-*d*_6_, ppm) δ 2.25 (3H, s, CH_3_), 4.04 (2H, s, CO–CH_2_), 5.02 (2H, s, N–CH_2_), 7.14 (2H, d, *J* = 8.3 Hz, Ar–H), 7.38–7.57 (9H, m, Ar–H), 8.16 (1H, d, *J* = 8.8 Hz, Ar–H), 10.30 (1H, s, NH). ^13^C NMR (75 MHz, DMSO-*d*_6_, ppm) δ 20.82, 37.98, 52.53, 117.93, 121.14, 123.97, 129.47, 129.95, 130.26, 130.44, 131.41, 133.41, 138.46, 140.47, 145.35, 147.16, 152.04, 165.42, 167.28. HRMS (*m*/*z*): [M + H]^+^ calcd for C_24_H_20_ClN_5_O_3_S_2_: 526.0769; found 526.0755.

##### 3.1.6.9 *N*-(4-Fluorophenyl)-2-[(5-(ethylamino)-1,3,4-thiadiazol-2-yl)thio]-*N*-(4-nitrobenzyl)acetamide (**6i**)

Yield 71%. M.p.: 132.3 °C.^1^H NMR (300 MHz, DMSO-*d*_6_, ppm) δ 1.15 (3H, t, *J* = 7.1 Hz, CH_2_–CH_3_), 3.21–3.30 (2H, m, CH_2_-CH_3_), 3.91 (2H, s, CO–CH_2_), 4.99 (2H, s, N–CH_2_), 7.25 (2H, t, *J* = 8.7 Hz, Ar–H), 7.35–7.40 (2H, m, Ar–H), 7.52 (1H, d, *J* = 8.7 Hz, Ar–H), 7.78 (1H, t, *J* = 5.3 Hz, NH), 8.15 (2H, d, *J* = 8.9 Hz, Ar–H). ^13^C NMR (75 MHz, DMSO-*d*_6_, ppm) δ 14.65, 38.20, 39.86, 52.66, 117.04 (d, *J* = 22.5 Hz), 123.95, 129.47, 130.77 (d, *J* = 8.8 Hz), 137.91 (d, *J* = 2.8 Hz), 145.40, 147.13, 149.20, 161.82 (d, *J* = 244.3 Hz), 167.54, 169.83. HRMS (*m*/*z*): [M + H]^+^ calcd for C_19_H_18_FN_5_O_3_S_2_: 448.0908; found 448.0898.

##### 3.1.6.10 *N*-(4-Fluorophenyl)-2-[(5-((2-methoxyethyl)amino)-1,3,4-thiadiazol-2-yl)thio]-*N*-(4-nitrobenzyl) Acetamide (**6j**)

Yield 68%. M.p.: 134.1 °C.^1^H NMR (300 MHz, DMSO-*d*_6_, ppm) δ 3.26 (3H, s, OCH_3_), 3.41–3.49 (4H, m, CH_2_), 3.91 (2H, s, CO–CH_2_), 4.99 (2H, s, N–CH_2_), 7.24 (2H, t, *J* = 8.8 Hz, Ar–H), 7.35–7.40 (2H, m, Ar–H), 7.53 (2H, d, *J* = 8.6 Hz, Ar–H), 7.88 (1H, t, *J* = 5.1 Hz, NH), 8.15 (2H, d, *J* = 8.8 Hz, Ar–H). ^13^C NMR (75 MHz, DMSO-*d*_6_, ppm) δ 38.13, 44.42, 52.67, 58.40, 70.40, 117.04 (d, *J* = 22.5 Hz), 123.95, 129.47, 130.77 (d, *J* = 9.0 Hz), 137.91 (d, *J* = 2.9 Hz), 145.41, 147.13, 149.61, 161.82 (d, *J* = 244.3 Hz), 167.54, 169.79. HRMS (*m*/*z*): [M + H]^+^ calcd for C_20_H_20_FN_5_O_4_S_2_: 478.1014; found 478.0993.

##### 3.1.6.11 *N*-(4-Fluorophenyl)-2-[(5-(propylamino)-1,3,4-thiadiazol-2-yl)thio]-*N*-(4-nitrobenzyl) Acetamide (**6k**)

Yield 80%. M.p.: 130.7 °C.^1^H NMR (300 MHz, DMSO-*d*_6_, ppm) δ 0.89 (3H, t, *J* = 7.4 Hz, CH_3_), 1.55 (2H, q, *J* = 7.1 Hz, CH_2_), 3.19 (2H, q, *J* = 5.4 Hz, CH_2_), 3.91 (2H, s, CO–CH_2_), 4.99 (2H, s, N–CH_2_), 7.24 (2H, t, *J* = 8.6 Hz, Ar–H), 7.35–7.40 (2H, m, Ar–H), 7.53 (2H, d, *J* = 8.6 Hz, Ar–H), 7.81 (1H, t, *J* = 5.4 Hz, NH), 8.15 (2H, d, *J* = 8.8 Hz, Ar–H). ^13^C NMR (75 MHz, DMSO-*d*_6_, ppm) δ 11.80, 22.22, 38.16, 46.83, 52.67, 117.03 (d, *J* = 22.9 Hz), 123.94, 129.46, 130.76 (d, *J* = 8.9 Hz), 137.92 (d, *J* = 2.9 Hz), 145.41, 147.12, 149.09, 161.82 (d, *J* = 244.3 Hz), 167.54, 170.02. HRMS (*m*/*z*): [M + H]^+^ calcd for C_20_H_20_FN_5_O_3_S_2_: 462.1064; found 462.1047.

##### 3.1.6.12 *N*-(4-Fluorophenyl)-2-[(5-(isopropylamino)-1,3,4-thiadiazol-2-yl)thio]-*N*-(4-nitrobenzyl)acetamide (**6l**)

Yield 75%. M.p.: 117.9 °C.^1^H NMR (300 MHz, DMSO-*d*_6_, ppm) δ 1.17 (6H, d, *J* = 6.4 Hz, 2CH_3_), 3.68–3.84 (1H, m, CH), 3.91 (2H, s, CO–CH_2_), 4.99 (2H, s, N–CH_2_), 7.25 (2H, t, *J* = 8.8 Hz, Ar–H), 7.35–7.40 (2H, m, Ar–H), 7.53 (1H, d, *J* = 8.7 Hz, Ar–H), 7.71 (1H, d, *J* = 7.1 Hz, NH), 8.16 (2H, d, *J* = 8.8 Hz, Ar–H). ^13^C NMR (75 MHz, DMSO-*d*_6_, ppm) δ 22.55, 38.15, 46.99, 52.67, 117.04 (d, *J* = 22.8 Hz), 123.95, 129.47, 130.77 (d, *J* = 8.7 Hz), 137.92 (d, *J* = 2.8 Hz), 145.41, 147.13, 149.06, 161.82 (d, *J* = 244.2 Hz), 167.55, 169.00. HRMS (*m*/*z*): [M + H]^+^ calcd for C_20_H_20_FN_5_O_3_S_2_: 462.1064; found 462.1055.

##### 3.1.6.13 *N*-(4-Fluorophenyl)-2-[(5-(butylamino)-1,3,4-thiadiazol-2-yl)thio]-*N*-(4-nitrobenzyl) Acetamide (**6m**)

Yield 66%. M. p.: 125.3 °C.^1^H NMR (300 MHz, DMSO-*d*_6_, ppm) δ 0.88 (3H, t, *J* = 7.4 Hz, CH_3_), 1.26–1.39 (2H, m, CH_2_), 1.47–1.57 (2H, m, CH_2_), 3.19–3.26 (2H, m, CH_2_), 3.90 (2H, s, CO–CH_2_), 4.99 (2H, s, N–CH_2_), 7.24 (2H, t, *J* = 8.8 Hz, Ar–H), 7.35–7.39 (2H, m, Ar–H), 7.53 (1H, d, *J* = 8.8 Hz, Ar–H), 7.78 (1H, t, *J* = 5.4 Hz, NH), 8.15 (2H, d, *J* = 8.8 Hz, Ar–H). ^13^C NMR (75 MHz, DMSO-*d*_6_, ppm) δ 14.07, 20.00, 31.00, 38.15, 44.72, 52.67, 117.03 (d, *J* = 22.6 Hz), 123.94, 129.47, 130.76 (d, *J* = 8.8 Hz), 137.93 (d, *J* = 2.8 Hz), 145.42, 147.13, 149.07, 161.82 (d, *J* = 244.3 Hz), 167.54, 170.00. HRMS (*m*/*z*): [M + H]^+^ calcd for C_21_H_22_FN_5_O_3_S_2_: 476.1221; found 476.1215.

##### 3.1.6.14 *N*-(4-Fluorophenyl)-2-[(5-(isobutylamino)-1,3,4-thiadiazol-2-yl)thio]-*N*-(4-nitrobenzyl) Acetamide (**6n**)

Yield 69%. M.p.: 145.5 °C.^1^H NMR (300 MHz, DMSO-*d*_6_, ppm) δ 0.89 (6H, d, *J* = 6.7 Hz, 2CH_3_), 1.78–1.95 (1H, m, CH), 3.04–3.09 (2H, m, CH_2_), 3.91 (2H, s, CO–CH_2_), 4.99 (2H, s, N–CH_2_), 7.24 (2H, t, *J* = 8.9 Hz, Ar–H), 7.35–7.39 (2H, m, Ar–H), 7.53 (2H, d, *J* = 8.7 Hz, Ar–H), 7.83 (1H, t, *J* = 5.6 Hz, NH), 8.15 (2H, d, *J* = 8.8 Hz, Ar–H). ^13^C NMR (75 MHz, DMSO-*d*_6_, ppm) δ 20.49, 27.95, 38.10, 52.66, 117.03 (d, *J* = 22.8 Hz), 123.93, 129.46, 130.75 (d, *J* = 8.9 Hz), 137.94 (d, *J* = 2.8 Hz), 145.42, 147.12, 149.04, 161.82 (d, *J* = 244.3 Hz), 167.55, 170.17. HRMS (*m*/*z*): [M + H]^+^ calcd for C_21_H_22_FN_5_O_3_S_2_: 476.1221; found 476.1198.

##### 3.1.6.15 *N*-(4-Fluorophenyl)-2-[(5-(cyclohexylamino)-1,3,4-thiadiazol-2-yl)thio]-*N*-(4-nitrobenzyl)acetamide (**6o**)

Yield 65%. M.p.: 113.9 °C.^1^H NMR (300 MHz, DMSO-*d*_6_, ppm) δ 1.14–1.36 (5H, m, CH_2_), 1.53–1.70 (3H, m, CH_2_), 1.92–1.96 (2H, m, CH_2_), 3.42–3.52 (1H, m, CH), 3.90 (2H, s, CO–CH_2_), 4.99 (2H, s, N–CH_2_), 7.24 (2H, t, *J* = 8.7 Hz, Ar–H), 7.35–7.39 (2H, m, Ar–H), 7.53 (2H, d, *J* = 8.7 Hz, Ar–H), 7.74 (1H, d, *J* = 7.3 Hz, NH), 8.15 (2H, d, *J* = 8.8 Hz, Ar–H). ^13^C NMR (75 MHz, DMSO-*d*_6_, ppm) δ 24.69, 25.68, 32.47, 38.06, 52.67, 53.90, 117.03 (d, *J* = 22.5 Hz), 123.93, 129.46, 130.75 (d, *J* = 8.8 Hz), 137.96 (d, *J* = 2.8 Hz), 145.43, 147.12, 148.94, 161.82 (d, *J* = 244.3 Hz), 167.54, 169.00. HRMS (*m*/*z*): [M + H]^+^ calcd for C_23_H_24_FN_5_O_3_S_2_: 502.1377; found 502.1360.

##### 3.1.6.16 *N*-(4-Fluorophenyl)-2-[(5-(phenylamino)-1,3,4-thiadiazol-2-yl)thio]-*N*-(4-nitrobenzyl)acetamide (**6p**)

Yield 70%. M.p.: 120.3 °C.^1^H NMR (300 MHz, DMSO-*d*_6_, ppm) δ 4.02 (2H, s, CO–CH_2_), 5.02 (2H, s, N–CH_2_), 6.99 (1H, t, *J* = 7.4 Hz, Ar–H), 7.23–7.42 (6H, m, Ar–H), 7.53–7.62 (3H, m, Ar–H), 7.71 (1H, d, *J* = 7.6 Hz, Ar–H), 8.09–8.12 (2H, m, Ar–H), 10.34 (1H, s, NH). ^13^C NMR (75 MHz, DMSO-*d*_6_, ppm) δ 38.18, 52.36, 117.07 (d, *J* = 22.8 Hz), 117.84, 122.42, 122.83, 123.28, 129.56, 130.37, 130.91 (d, *J* = 8.9 Hz), 135.25, 137.70 (d, *J* = 2.8 Hz), 139.74, 140.84, 148.22, 152.58, 161.88 (d, *J* = 245.8 Hz), 165.14, 167.47. HRMS (*m*/*z*): [M + H]^+^ calcd for C_23_H_18_FN_5_O_3_S_2_: 496.0908; found 496.0894.

##### 3.1.6.17 *N*-(4-Fluorophenyl)-2-[(5-(4-methylphenylamino)-1,3,4-thiadiazol-2-yl)thio]-*N*-(4-nitrobenzyl) Acetamide (**6q**)

M. p.: 153.0 °C. Yield 64%. ^1^H NMR (300 MHz, DMSO-*d*_6_, ppm) δ 2.26 (3H, s, CH_3_), 4.02 (2H, s, CO–CH_2_), 5.01 (2H, s, N–CH_2_), 7.15 (2H, d, *J* = 8.3 Hz, Ar–H), 7.27 (2H, t, *J* = 8.8 Hz, Ar–H), 7.39–7.47 (4H, m, Ar–H), 7.55 (2H, d, *J* = 8.8 Hz, Ar–H), 8.16 (2H, d, *J* = 8.8 Hz, Ar–H), 10.26 (1H, s, NH). ^13^C NMR (75 MHz, DMSO-*d*_6_, ppm) δ 20.84, 38.00, 52.67, 117.09 (d, *J* = 22.7 Hz), 117.93, 123.95, 129.51, 129.96, 130.82 (d, *J* = 8.8 Hz), 131.43, 137.90 (d, *J* = 2.7 Hz), 138.45, 145.41, 147.16, 152.10, 161.86 (d, *J* = 244.2 Hz), 165.41, 167.40. HRMS (*m*/*z*): [M + H]^+^ calcd for C_24_H_20_FN_5_O_3_S_2_: 510.1064; found 510.1049.

### 3.2 Biological Activity

#### 3.2.1 Aldose Reductase Assay

According to previous studies, the AR enzyme was purified from sheep liver.^57^ The assay was conducted using modified methods from previous studies.^58,59^ Aldose reductase (AR) activity was evaluated by monitoring the reduction in absorbance at 340 nm, which corresponds to the consumption of NADPH. The reaction mixture for AR activity included 0.8 M sodium phosphate buffer (pH 5.5), 0.11 mM NADPH, 4.7 mM dl-glyceraldehyde, and the enzyme solution.

#### 3.2.2 α-Glycosidase Assay

α-Glucosidase activity (α-Glucosidase from *Saccharomyces cerevisiae*) was assessed using p-nitrophenyl-d-glycopyranoside (pNPG) as the substrate, following the procedure outlined by Tao et al.^60,61^ Initially, 100 μL of phosphate buffer was mixed with 20 μL of the enzyme solution (0.15 U/mL, pH 6.8) and 10–100 μL (0.01–1 mg/mL^–1^) of the sample. To ensure complete enzyme inhibition, multiple solutions were prepared in phosphate buffer. The mixture was preincubated at 35 °C for 12 min before the reaction was initiated by adding pNPG. Subsequently, 50 μL of pNPG solution (5.0 mM, pH 7.4) was added and incubated at 37 °C. Absorbance was measured spectrophotometrically at 405 nm.

#### 3.2.3 *In Vitro* Inhibition Studies

The inhibitory effects of novel 1,3,4-thiadiazole derivatives (**6a**–**6q**) were assessed at multiple concentrations against AR and α-GLY enzymes, with at least five distinct inhibitor concentrations used. The IC_50_ values for the derivatives against the enzymes were determined by plotting the percentage of activity versus inhibitor concentration (1–200 nM for AR and 1–500 μM for α-GLY) in Excel. Furthermore, the types of inhibition and inhibition constants (KI) were calculated from Lineweaver–Burk plots, providing detailed insights into the kinetic properties of these inhibitors.^62,63^

### 3.3 Molecular Docking Study

An important method used to identify molecules with high activity against biological materials is docking. The crystal structure of α-GLY (PDB ID:5NN8, Crystal structure of human lysosomal acid- α-GLY, GAA, in complex with acarbose, Method: X-ray Diffraction, Resolution: 2.45 Å) and AR (PDB ID: 4JIR, Crystal Structure of AR (AKR1B1) Complexed with NADP^+^ and epalrestat, Method: X-ray Diffraction, Resolution: 2.00 Å) were retrieved from the PDB database (http://www.rcsb.org/pdb). The structure of α-Glucosidase enzyme, PDB ID: 5NN8, was chosen because of its high resolution (2.45 Å) and the fact that it was solved in complex with acarbose. Molecular docking calculations were performed with Schrödinger’s Maestro Molecular modeling platform. First, the protein preparation module is used to prepare the protein and then the LigPrep module is used to prepare the molecule. The prepared proteins and molecules are also interacted with each other by Glide ligand docking.^64^

### 3.4 ADME Analysis

The Swiss ADME online web tool (http://www.swissadme.ch/) and Admetlab (https://admetmesh.scbdd.com/) were used to perform ADME analysis of the synthesized compounds (**6h**, **6o**, and **6p**). The canonical SMILES of these compounds were generated from ChemDraw and prediction of the physicochemical properties of these compounds including lipophilicity, drug similarity, pharmacokinetics, TPSA, number of rotatable bonds, and violations of Lipinski’s five rules were performed.^49,65^ ADME/T analysis was performed in order to examine the effects and effects of the studied molecules on human metabolism.

### 3.5 Statistical Studies

Data analysis and graphical presentations were performed using GraphPad Prism version 8 for Windows (GraphPad Software, La Jolla, California), a software renowned for its powerful statistical features and intuitive interface. In biological experiments (enzyme inhibition, cytotoxicity), measurements were performed in 3 independent replicates. The analysis involved descriptive statistics, with results reported as means ± standard error of the mean (SEM), offering an indication of the variability around the mean values.

### 3.6 Cell Culture

Healthy mouse fibroblast cell line (L929) was obtained from ATCC (American Type Culture Collection) and studied. Cells were mixed with 89% DMEM (Dulbecco’s modified Eagle’s medium; Gibco, Thermo Fisher Scientific), 10% FBS (Fetal Bovine Serum; Sigma-Aldrich) and 1% penicillin (Sigma-Aldrich) solutions. The cells in which the medium was added were allowed to grow by incubating at 37 °C in an environment containing 95% humidity and 5% CO_2_.^66,67^

#### 3.6.1 Cell Viability Assay

The cytotoxic effects of all syntheses by MTT analysis on L929 cell line was investigated. 96-well plates were used for seeding cells. Approximately 1 × 10^4^ cells were seeded in each well. Cells were allowed to adhere for 24 h and then the syntheses were applied at different concentrations (5–100 μM). After adding syntheses at different concentrations, the wells were incubated for 24 h. All wells without syntheses were used as controls. After the incubation, the wells were treated with MTT solution to determine metabolically active cells and incubated at 37 °C for 3 h. After the MTT interaction, the wells were emptied and DMSO solution was placed in them. The formazan crystals formed were dissolved with this solution and the number of viable cells in each well was determined by color change. The absorbance values were read at 540 nm with the help of a microplate and the values found were represented as mean ± standard deviation (±SD).^66,67^

### 3.7 Ames II Test

Ames II Mutagenicity Assay Kit BioReliance and Moltox (Moltox, Boone, NC) kit were used in the experiment and the experiment was performed completely according to the kit procedure.^68^*S. typhimurium* TA 98 (hisD3052) and TA mix (hisG1775, hisC9138, hisG9074, hisG9133, hisG9130, hisC9070) strains were incubated in 50 mL tubes containing 10 mL growth medium at 37 °C for 24 h. At the end of the time, the OD600 value of the suspension was measured. Since the OD600 value ≥ 2.0, the experimental procedure was continued. The dilutions of 10 μL of compound **6h** at 75, 37.5, 18.75, 9.4, 4.7 μM and compound **6o** at 50, 25, 12.5, 6.25, 3.125 nM were prepared at 5 different concentrations of the test substances dissolved in DMSO. The exposure plates (24 wells) with and without the S9 enzyme fraction were prepared for both strains. The exposure medium, bacteria, and test substance were added to the plates without S9 enzyme fraction in the amounts specified in the procedure; S9 enzyme fraction was added to the plates containing S9 enzyme fraction and incubated at 37 °C, 250 rpm for 90 min with shaking. At the end of the incubation period, 2.8 mL of purple colored indicator medium specific for bacterial strains was added to each well of the plate. 4-Nitroquinoline-*N*-Oxide (4-NQO) - 2-Nitrofluorene (2-NF), and 2-Aminoanthracene (2-AA) were used as positive control and only DMSO was used as negative control. The contents of 50 μL in each well were transferred to 384 plates according to the determined experimental design and incubated at 37 °C for 48 h. At the end of the incubation period, bacterial metabolism in the plates changes the pH and changes the purple color to yellow. The number of yellow wells formed at the end of the experiment was determined as positive wells and the genotoxic properties of the substances were interpreted. Means and SD values of positive wells were calculated.^69^

## 4 Conclusions

This study involved the synthesis of a novel 1,3,4-thiadiazole series and the assessment of their inhibitory effects against the enzymes α-GLY and AR, the molecular docking and ADME/T studies, as well as cytotoxic activity evaluation. Against aldose reductase, all of the synthesized compounds showed remarkable inhibition profiles with *K*_I_ values of 15.39 ± 1.61 to 176.50 ± 10.69 nM and IC_50_ values of 20.16 ± 1.07 to 175.40 ± 6.97 nM while reference inhibitor epalrestat having a *K*_I_ value of 837.70 ± 53.87 nM and IC_50_ value of 265.00 ± 2.26 nM. In addition, some of the compounds (**6a**, **6g**, **6h**, **6j**, **6o**, **6p**, and **6q**) showed significantly higher α-glucosidase inhibitory activity (*K*_I_: 4.48 ± 0.25 μM–15.86 ± 0.92 μM and IC_50_: 4.68 ± 0.23 μM–34.65 ± 1.78 μM) compared to the reference acarbose (*K*_I_: 21.52 ± 2.72 μM, IC_50_: 132.51 ± 9.86 μM). Compound **6h** with *p*-methylphenyl group was the most potent compound toward α-GLY with the *K*_*I*_ value of 4.48 ± 0.25 μM, while compounds **6o** with cyclohexyl group and **6p** with phenyl group were found to be most effective compounds against AR, with the *K*_I_ values of 15.39 ± 1.61 and 23.86 ± 2.41 nM, respectively. Molecular docking studies confirmed that compounds **6h**, **6o**, and **6p** interact with their targets through hydrogen bonds as in standard compounds. In addition, the ADME/T study and cytotoxicity assay of compounds support the potential of the these compounds as antidiabetic agents with favorable pharmacokinetic profiles. An AMES test has been added to show the low mutagenic potential of the active compounds.

## Supporting Information Available

^1^H NMR, ^13^C NMR, and HRMS spectra of compounds **6a–6q** (PDF)
