# Supplementary material for: Design, Synthesis, Biological Evaluation, and Molecular Docking Studies of Novel 1,3,4-Thiadiazole Derivatives Targeting Both Aldose Reductase and α-Glucosidase for Diabetes Mellitus
Source: ACS Omega. 2025 May 5;10(18):18812–28. doi: 10.1021/acsomega.5c00566 (PMC12079242; doi:10.1021/acsomega.5c00566)
Supplement: Supplementary file 1 — ao5c00566_si_001.pdf [file ao5c00566_si_001.pdf]

Supplementary Material

Design, Synthesis, Biological Evaluation, and Molecular Docking Studies of Novel 1,3,4-Thiadiazole Derivatives Targeting both Aldose Reductase and α-Glucosidase for Diabetes Mellitus

Betül Kaya[1], Ulviye Acar Çevik[2*], Adem Necip[3], Hatice Esra Duran[4], Bilge Çiftçi[5], Mesut Işık[6], Pervin Soyer[7], Hayrani Eren Bostancı[8], Zafer Asım Kaplancıklı[2,10], Şükrü Beydemir[9]

[1]Department of Pharmaceutical Chemistry, Faculty of Pharmacy, Zonguldak Bulent Ecevit University, 67600 Zonguldak, Turkey

[2]Department of Pharmaceutical Chemistry, Faculty of Pharmacy, Anadolu University, 26470 Eskişehir, Turkey

[3]Department of Pharmacy Services, Vocational School of Health Services, Harran University, 63300 Şanlıurfa, Turkey

[4]Department of Medical Biochemistry, Faculty of Medicine, Kafkas University, 36100 Kars, Turkey

[5]Vocational School of Health Services, Bilecik Şeyh Edebali University, 11230 Bilecik, Turkey

[6]Department of Bioengineering, Faculty of Engineering, Bilecik Şeyh Edebali University, 11230 Bilecik, Turkey

[7]Department of Pharmaceutical Microbiology, Faculty of Pharmacy, Anadolu University, Eskişehir, 26470 Turkey

[8]Department of Biochemistry, Faculty of Pharmacy, Cumhuriyet University, 58140 Sivas, Turkey

[9]Department of Biochemistry, Faculty of Pharmacy, Anadolu University, 26470 Eskişehir, Turkey

*Correspondence:

E-mail: uacar@anadolu.edu.tr; Tel. +90-222-335-0580/3775

Address: Anadolu University, Faculty of Pharmacy, Department of Pharmaceutical Chemistry, 26470 Eskişehir, Turkey

[10]The Rectorate of Bilecik Şeyh Edebali University, 11230 Bilecik, Turkey

Author Information

Corresponding Authors

Ulviye Acar Çevik-Department of Pharmaceutical Chemistry, Faculty of Pharmacy, Anadolu University, 26470 Eskişehir, Turkey. E-mail; uacar@anadolu.edu.tr

Authors

Betül Kaya- Department of Pharmaceutical Chemistry, Faculty of Pharmacy, Zonguldak Bulent Ecevit University, 67600 Zonguldak, Turkey. E-mail; betulkayaa91@gmail.com

Adem Necip- Department of Pharmacy Services, Vocational School of Health Services, Harran University, 63300 Şanlıurfa, Turkey. E-mail; ademnecip@harran.edu.tr

Hatice Esra Duran - Department of Medical Biochemistry, Faculty of Medicine, Kafkas University, 36100 Kars, Turkey. E-mail; hatice.duran@kafkas.edu.tr

Bilge Çiftçi - Vocational School of Health Services, Bilecik Şeyh Edebali University, 11230 Bilecik, Turkey. E-mail: bilge.ciftci@bilecik.edu.tr

Mesut Işık - Department of Bioengineering, Faculty of Engineering, Bilecik Şeyh Edebali University, 11230 Bilecik, Turkey. E-mail: mesut.isik@bilecik.edu.tr

Pervin Soyer - Department of Pharmaceutical Microbiology, Faculty of Pharmacy, Anadolu University, 26470 Eskişehir, Turkey. E-mail: pervinsoyer@anadolu.edu.tr

Hayrani Eren Bostancı - Department of Biochemistry, Faculty of Pharmacy, Cumhuriyet University, 58140 Sivas, Turkey. E-mail; erenbostanci@cumhuriyet.edu.tr

Zafer Asım Kaplancıklı- Department of Pharmaceutical Chemistry, Faculty of Pharmacy, Anadolu University, 26470 Eskişehir, Turkey; The Rectorate of Bilecik Şeyh Edebali University, Bilecik 11230, Turkey. E-mail; zakaplan@anadolu.edu.tr

Şükrü Beydemir - Department of Biochemistry, Faculty of Pharmacy, Anadolu University, 26470 Eskişehir, Turkey. E-mail; sukrubeydemir@anadolu.edu.tr

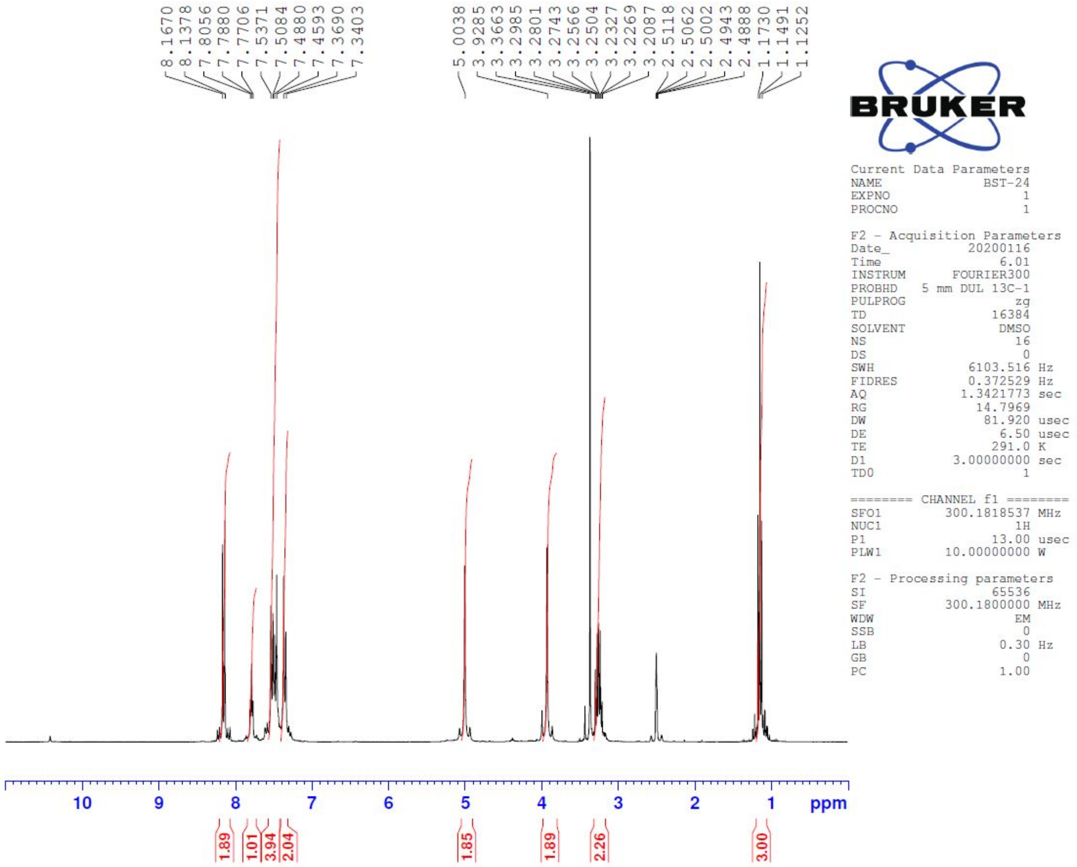

Figure S1. ^{1}H-NMR spectrum of compound **6a**

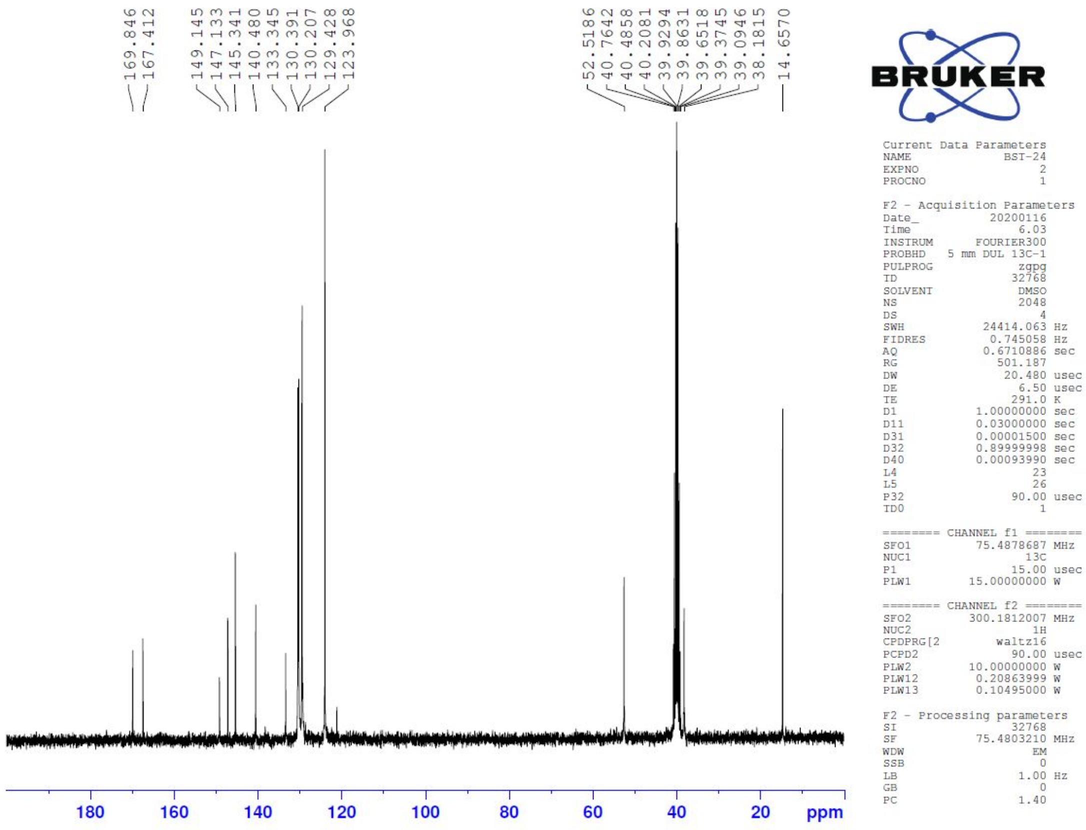

Figure S2. ^{13}C-NMR spectrum of compound **6a**

Formula Predictor Report - BST-24_15.lcd   Page 1 of 1

Data File: C:\LabSolutions\Data\Analiz\uac\BST-24_15.lcd

Elmt	Val.	Min	Max	Elmt	Val.	Min	Max	Elmt	Val.	Min	Max	Elmt	Val.	Min	Max	Use Adduct
H	1	6	30	O	2	3	4	S	2	2	3	Ru	2	0	0	H
C	4	8	30	F	1	0	1	Cl	1	0	1	Pd	2	0	0	
N	3	4	5	P	3	0	0	Br	1	0	0	I	3	0	0	

Error Margin (ppm):	5	DBE Range:	7.0 - 20.0	Electron Ions:	both
HC Ratio:	unlimited	Apply N Rule:	yes	Use MSn Info:	yes
Max Isotopes:	3	Isotope RI (%):	1.00	Isotope Res:	9000
MSn Iso RI (%):	10.00	MSn Logic Mode:	AND	Max Results:	100

Event#: 1 MS(E+) Ret. Time : 2.840 -> 3.627 Scan# : 427 -> 545

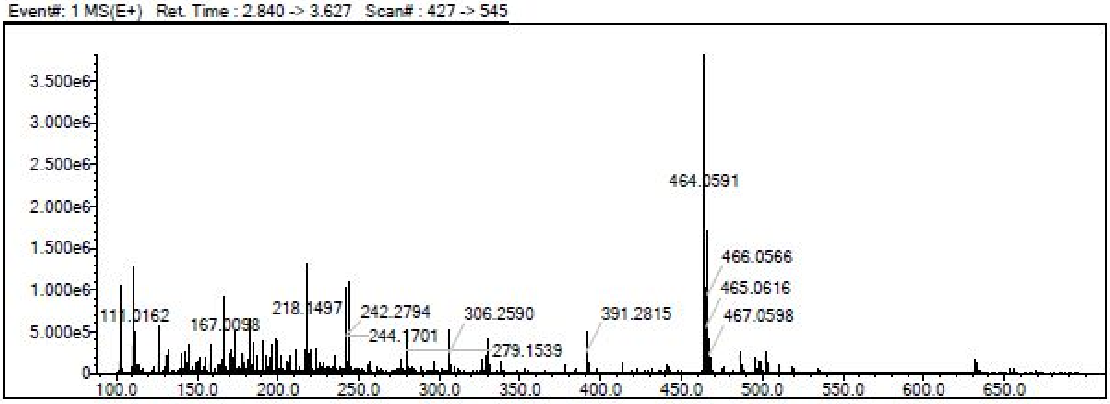

Measured region for 464.0591 m/z

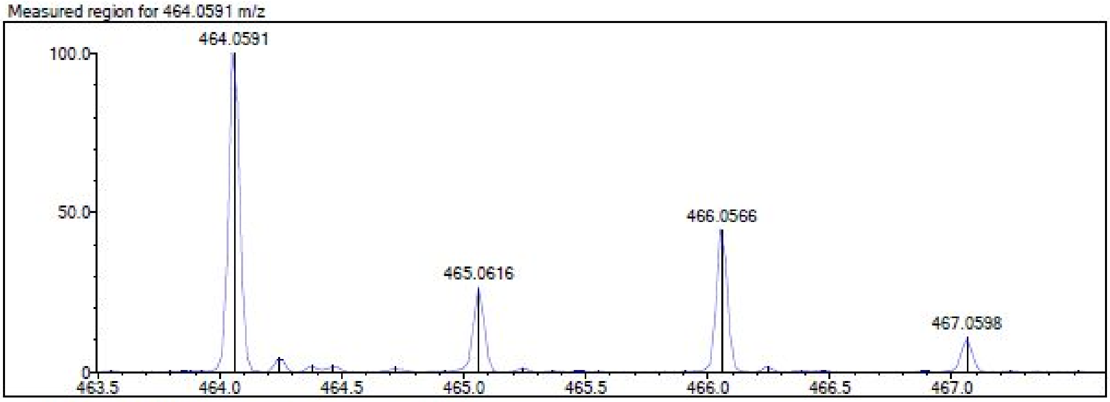

C19 H18 N5 O3 S2 Cl [M+H]+ : Predicted region for 464.0612 m/z

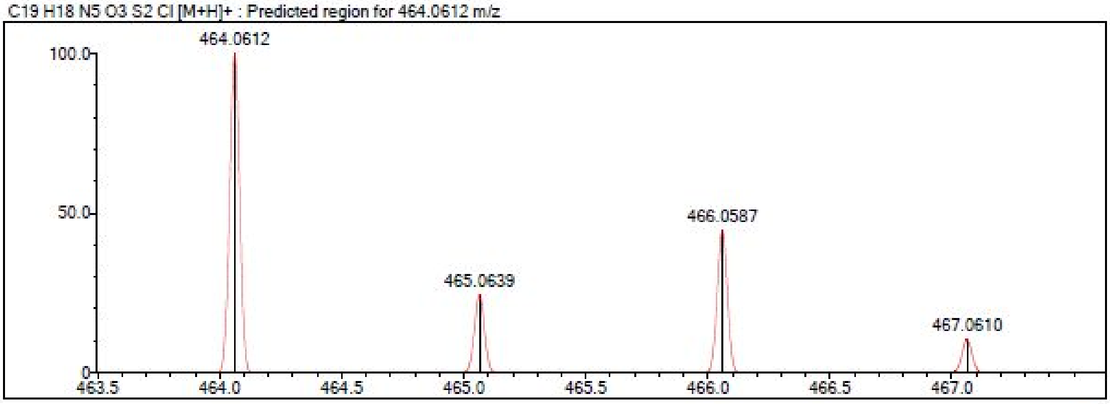

Rank	Score	Formula (M)	Ion	Meas. m/z	Pred. m/z	Df. (mDa)	Df. (ppm)	Iso	DBE
1	78.42	C19 H18 N5 O3 S2 Cl	[M+H]+	464.0591	464.0612	-2.1	-4.53	86.01	13.0

Figure S3. Mass spectrum of compound **6a**

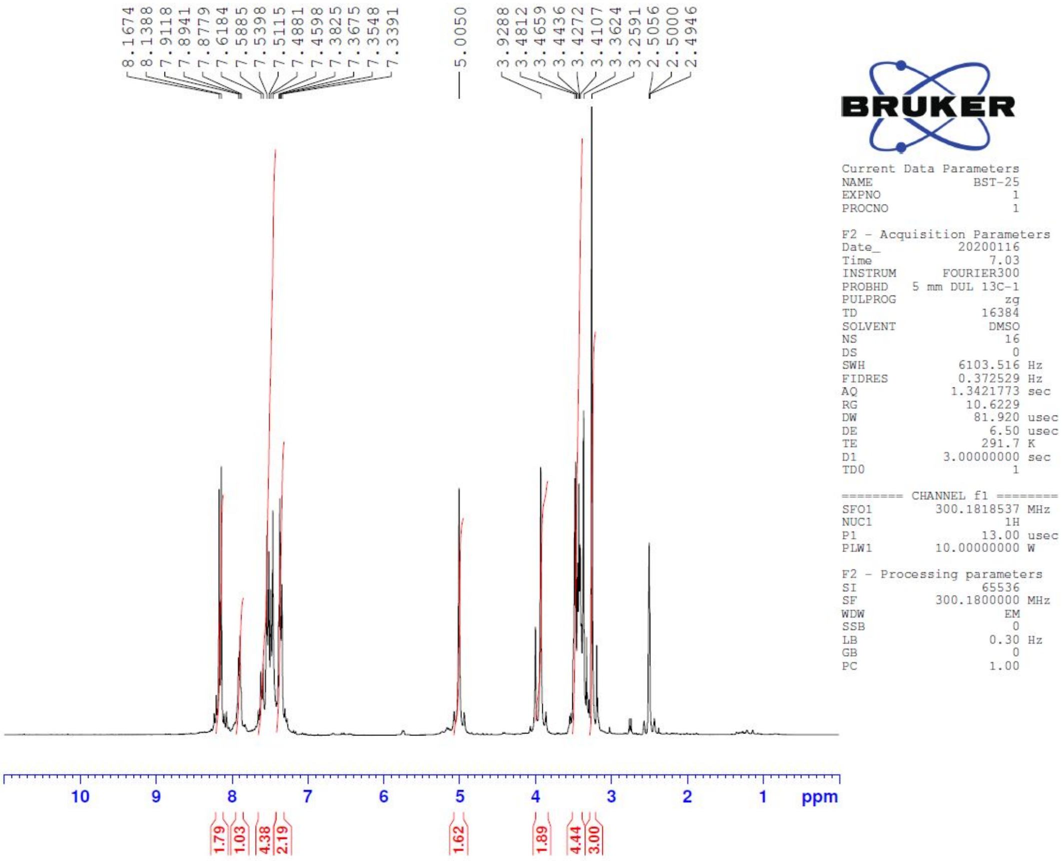

Figure S4. ^{1}H-NMR spectrum of compound **6b**

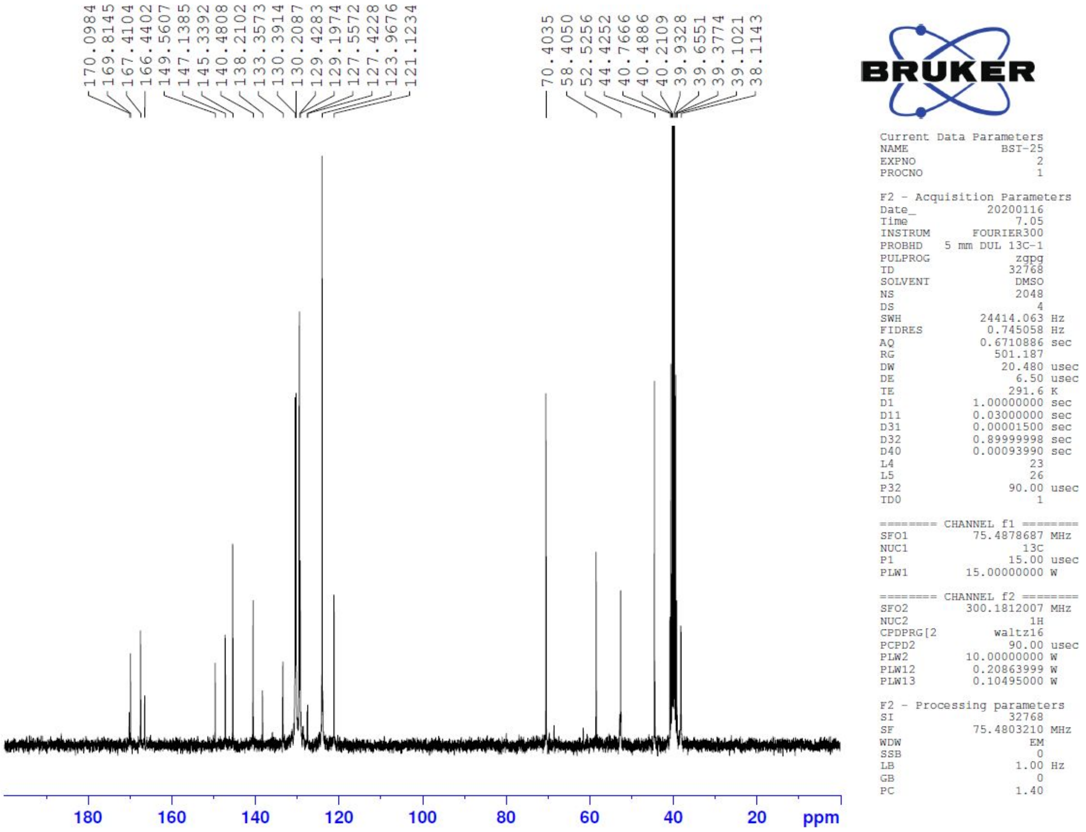

Figure S5. ^{13}C-NMR spectrum of compound **6b**

Formula Predictor Report - BST-25_16.lcd

Data File: C:\LabSolutions\Data\Analiz\uac\BST-25_16.lcd

Elmt	Val.	Min	Max	Elmt	Val.	Min	Max	Elmt	Val.	Min	Max	Elmt	Val.	Min	Max	Use Adduct
H	1	6	30	O	2	3	4	S	2	2	3	Ru	2	0	0	H
C	4	8	30	F	1	0	1	Cl	1	0	1	Pd	2	0	0	
N	3	4	5	P	3	0	0	Br	1	0	0	I	3	0	0	

Error Margin (ppm): 5
HC Ratio: unlimited
Max Isotopes: 3
MSn Iso RI (%): 10.00

DBE Range: 7.0 - 20.0
Apply N Rule: yes
Isotope RI (%): 1.00
MSn Logic Mode: AND

Electron Ions: both
Use MSn Info: yes
Isotope Res: 9000
Max Results: 100

Event#: 1 MS(E+) Ret. Time : 2.627 -> 3.027 Scan# : 395 -> 455

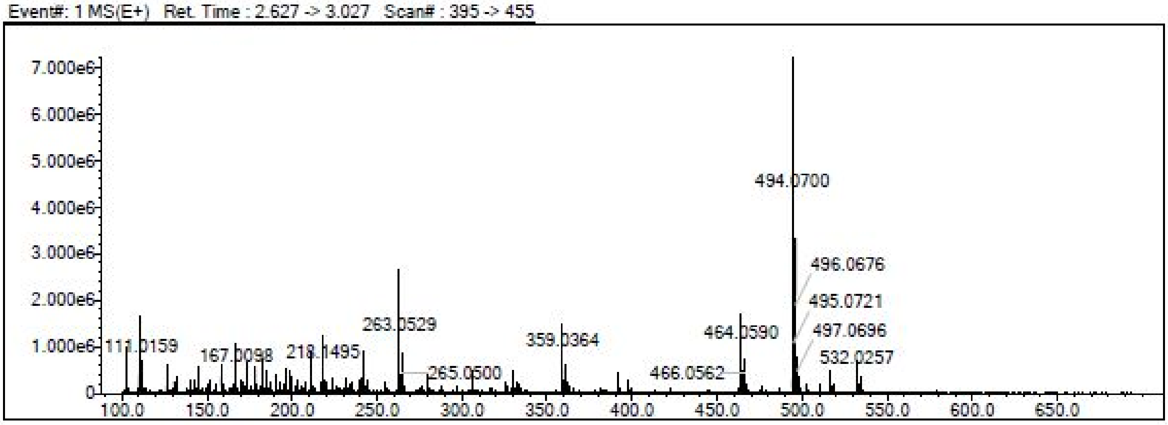

Measured region for 494.0700 m/z

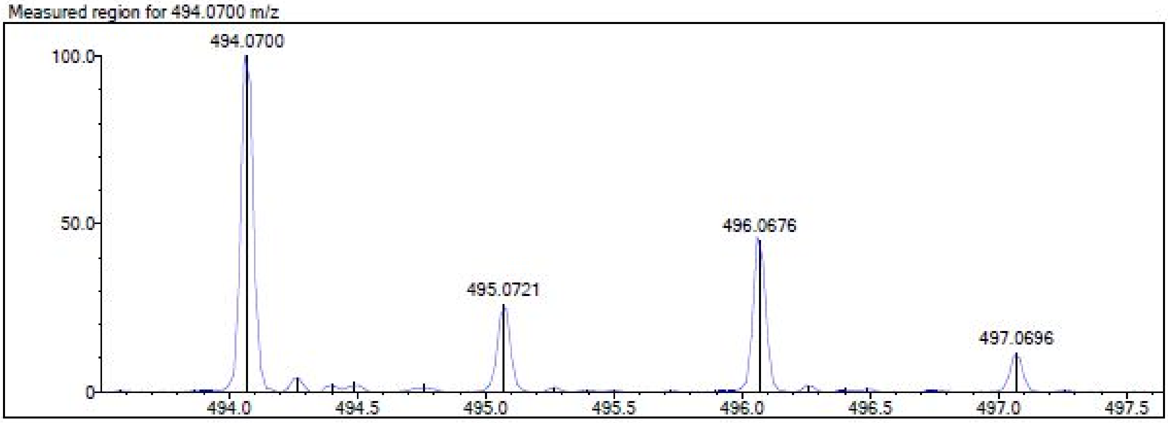

C20 H20 N5 O4 S2 Cl [M+H]+ : Predicted region for 494.0718 m/z

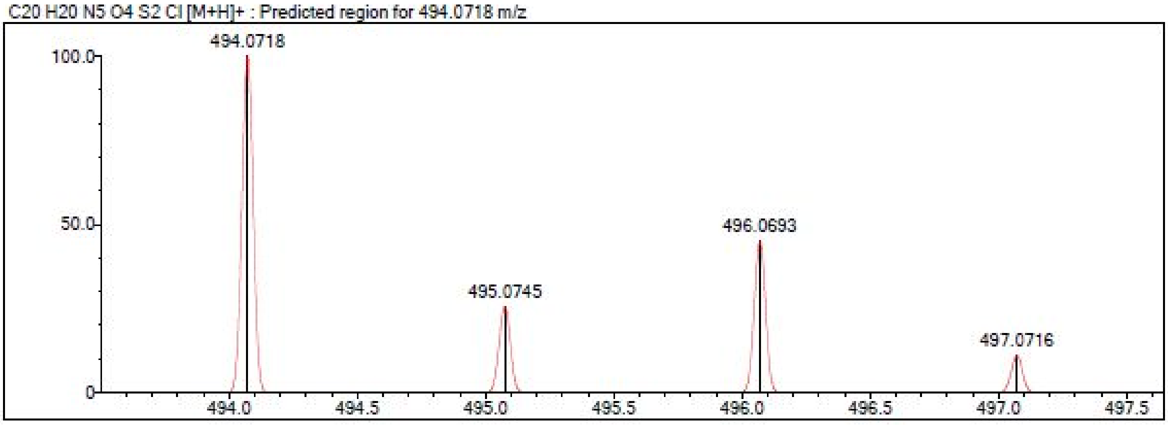

Rank	Score	Formula (M)	Ion	Meas. m/z	Pred. m/z	Df. (mDa)	Df. (ppm)	Iso	DBE
1	90.43	C20 H20 N5 O4 S2 Cl	[M+H]+	494.0700	494.0718	-1.8	-3.64	96.82	13.0

Figure S6. Mass spectrum of compound **6b**

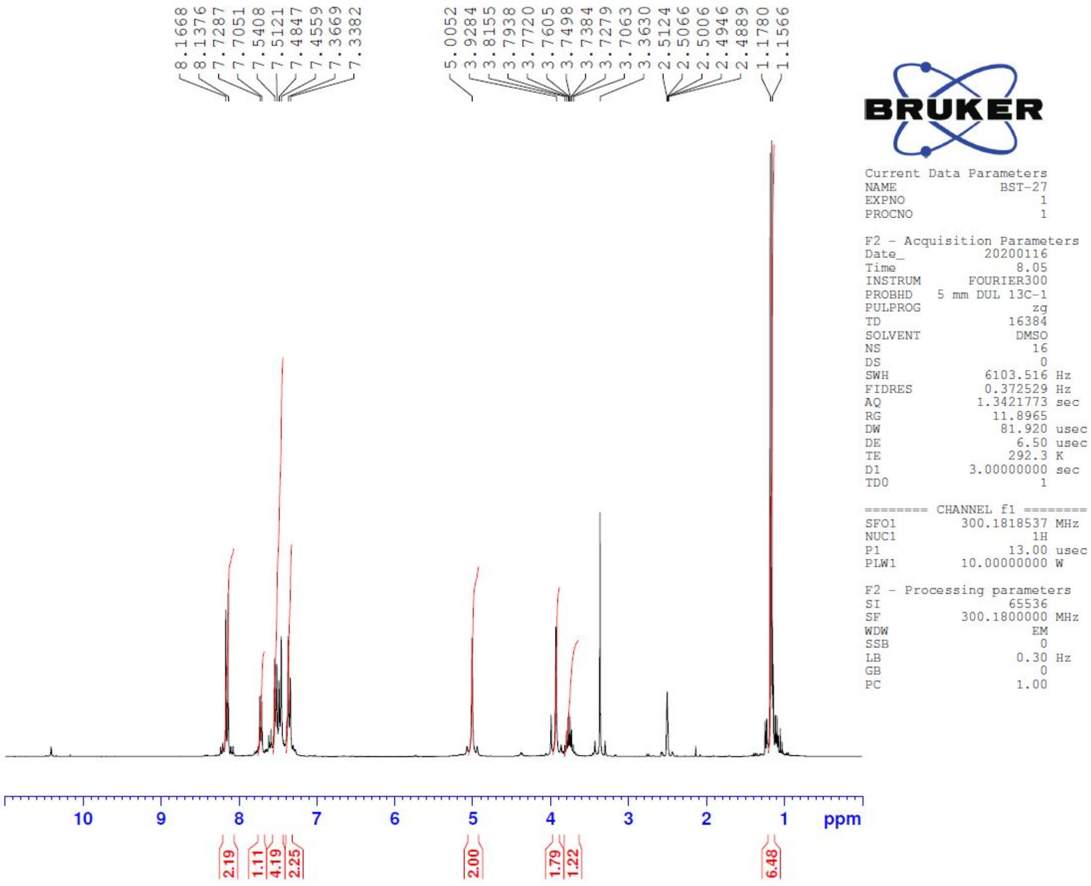

Figure S7. ^{1}H-NMR spectrum of compound **6c**

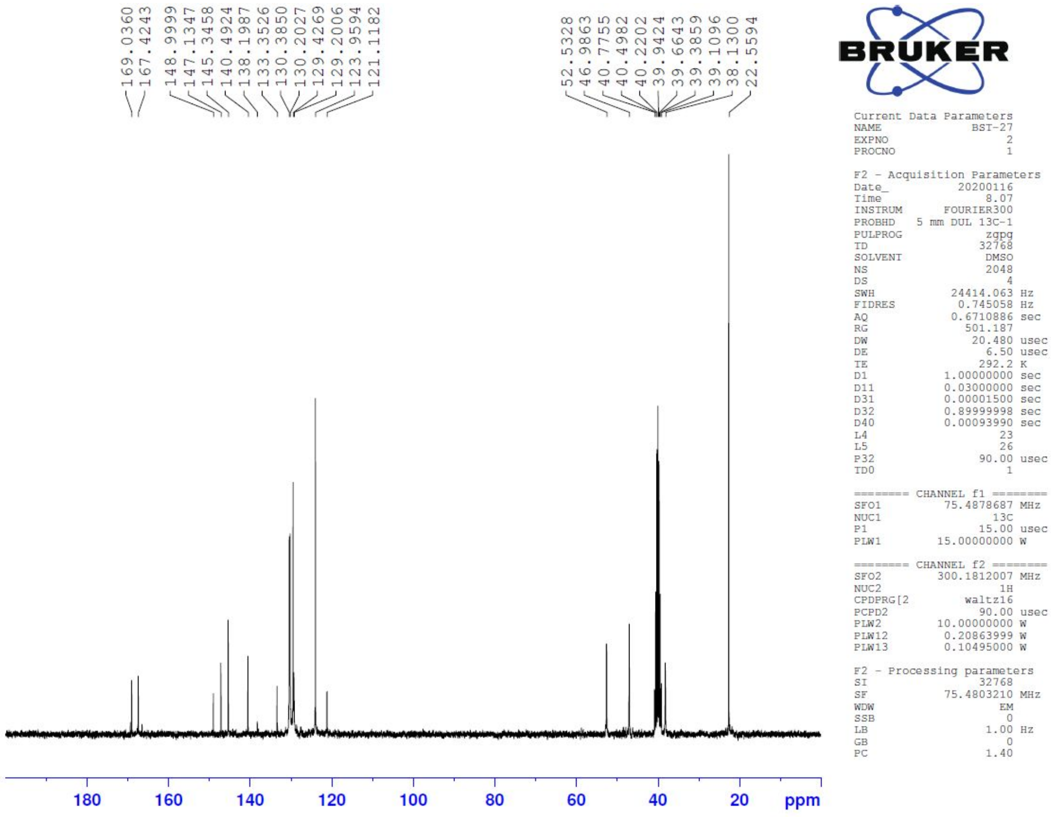

Figure S8. ^{13}C-NMR spectrum of compound **6c**

Data File: C:\LabSolutions\Data\Analiz\uac\BST-27_17.lcd

Elmt	Val.	Min	Max	Elmt	Val.	Min	Max	Elmt	Val.	Min	Max	Elmt	Val.	Min	Max	Use Adduct
H	1	6	30	O	2	3	4	S	2	2	3	Ru	2	0	0	H
C	4	8	30	F	1	0	1	Cl	1	0	1	Pd	2	0	0	
N	3	4	5	P	3	0	0	Br	1	0	0	I	3	0	0	

Error Margin (ppm):	5	DBE Range:	7.0 - 20.0	Electron Ions:	both
HC Ratio:	unlimited	Apply N Rule:	yes	Use MSn Info:	yes
Max Isotopes:	3	Isotope RI (%):	1.00	Isotope Res:	9000
MSn Iso RI (%):	10.00	MSn Logic Mode:	AND	Max Results:	100

Event#: 1 MS(E+) Ret. Time : 3.293 Scan# : 495

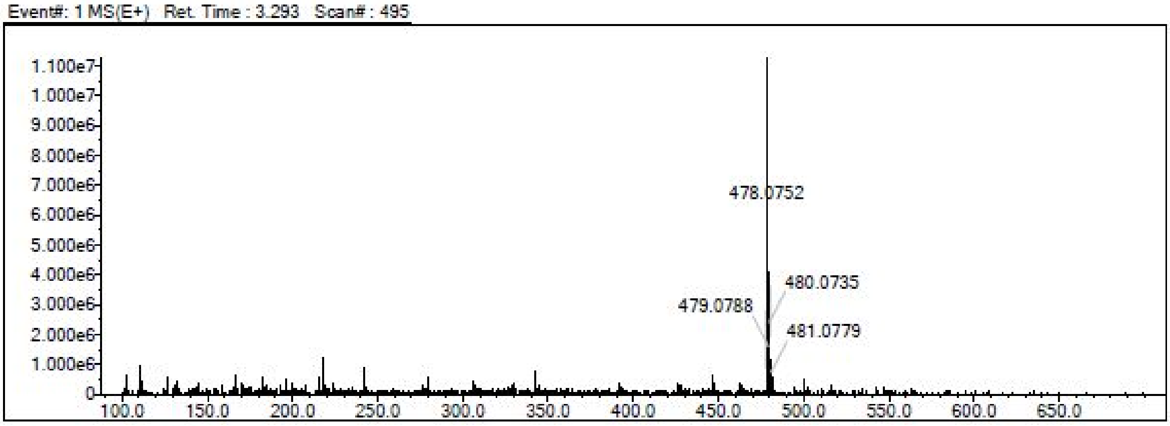

Measured region for 478.0752 m/z

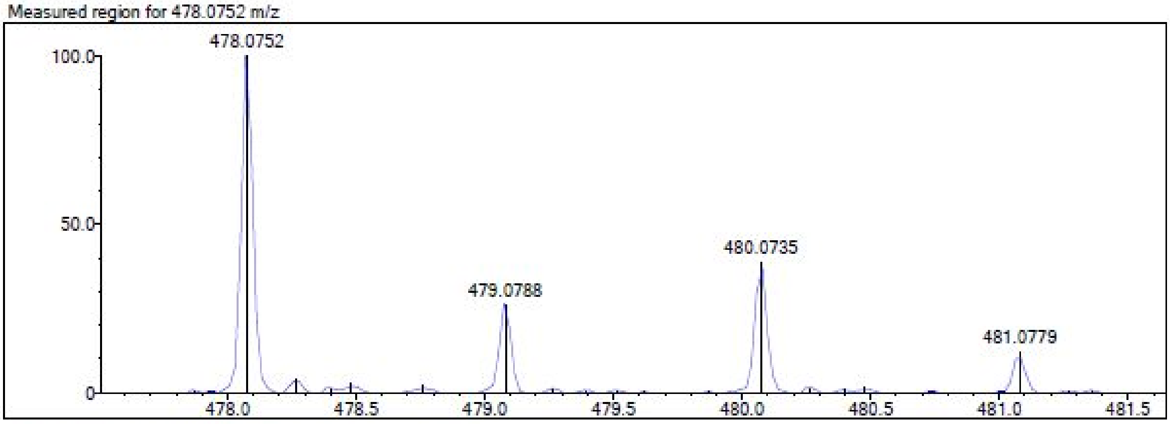

C20 H20 N5 O3 S2 Cl [M+H]+ : Predicted region for 478.0769 m/z

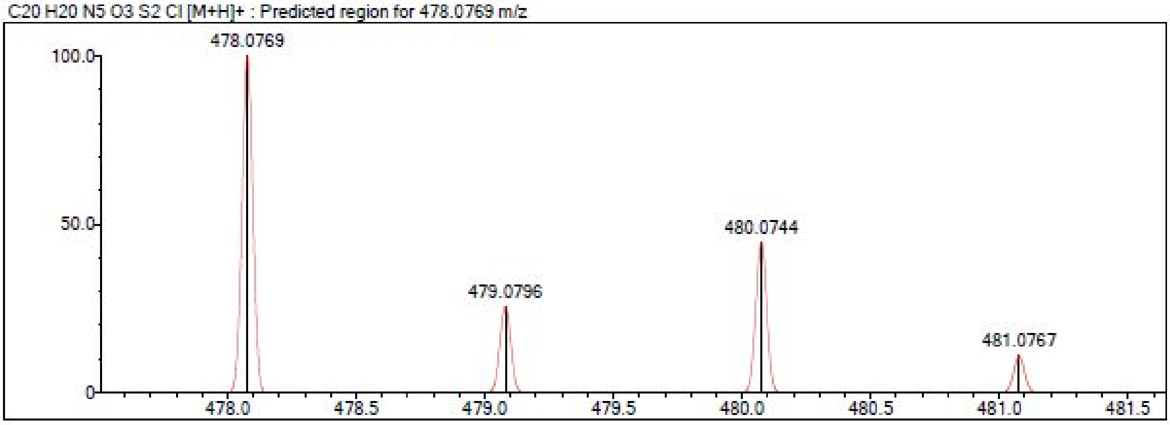

Rank	Score	Formula (M)	Ion	Meas. m/z	Pred. m/z	Df. (mDa)	Df. (ppm)	Iso	DBE
1	71.69	C20 H20 N5 O3 S2 Cl	[M+H]+	478.0752	478.0769	-1.7	-3.56	76.59	13.0

Figure S9. Mass spectrum of compound **6c**

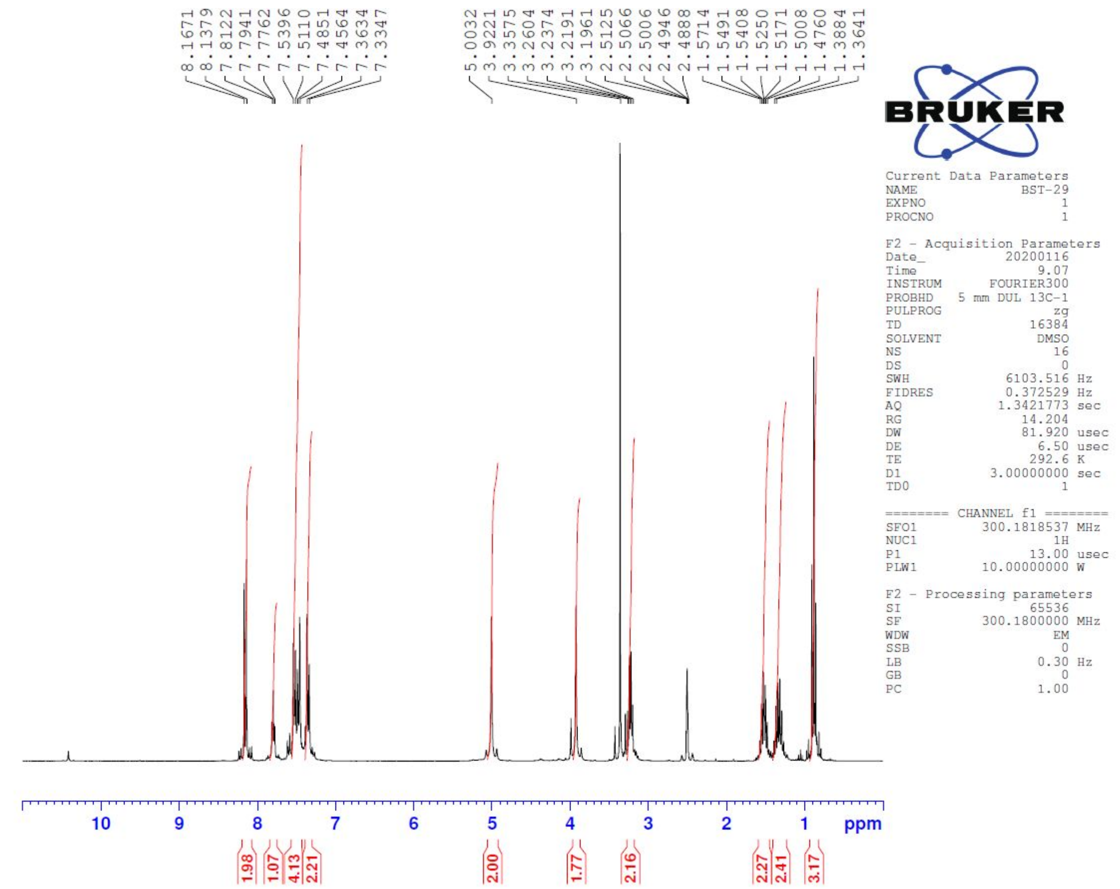

Figure S10. ^{1}H-NMR spectrum of compound **6d**

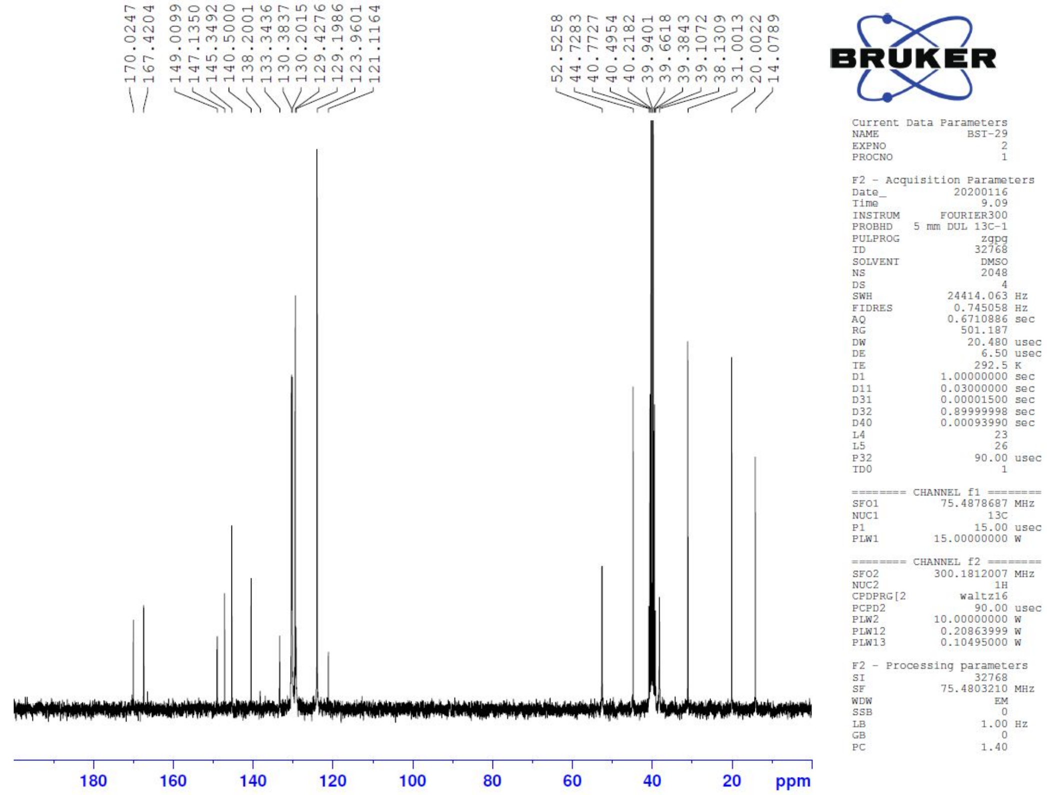

Figure S11. ^{13}C-NMR spectrum of compound **6d**

Data File: C:\LabSolutions\Data\Analiz\uac\BST-29_18.lcd

Elmt	Val.	Min	Max	Elmt	Val.	Min	Max	Elmt	Val.	Min	Max	Elmt	Val.	Min	Max	Use Adduct
H	1	6	30	O	2	3	4	S	2	2	3	Ru	2	0	0	H
C	4	8	30	F	1	0	1	Cl	1	0	1	Pd	2	0	0	
N	3	4	5	P	3	0	0	Br	1	0	0	I	3	0	0	

Error Margin (ppm):	5	DBE Range:	7.0 - 20.0	Electron Ions:	both
HC Ratio:	unlimited	Apply N Rule:	yes	Use MSn Info:	yes
Max Isotopes:	3	Isotope RI (%):	1.00	Isotope Res:	9000
MSn Iso RI (%):	10.00	MSn Logic Mode:	AND	Max Results:	100

Event#: 1 MS(E+) Ret. Time : 3.413 Scan# : 513

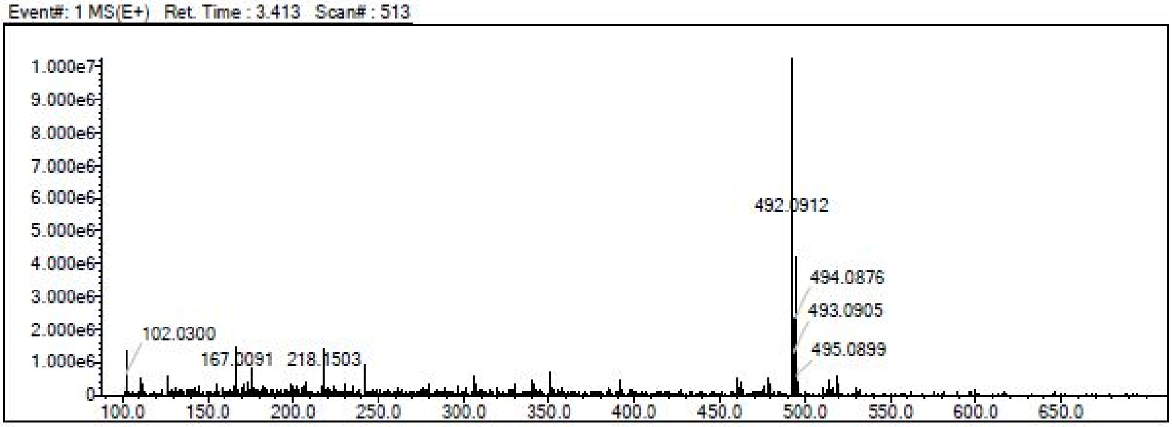

Measured region for 492.0912 m/z

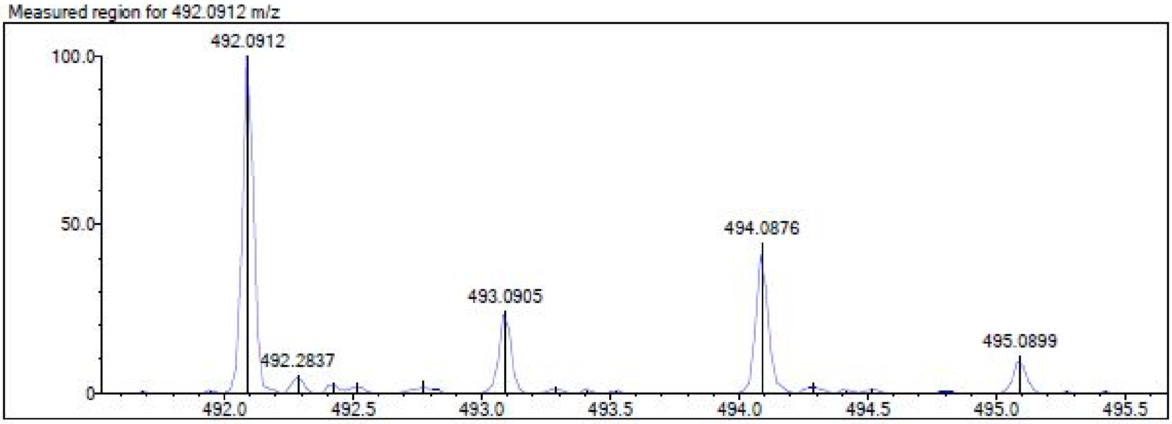

C21 H22 N5 O3 S2 Cl [M+H]+ : Predicted region for 492.0925 m/z

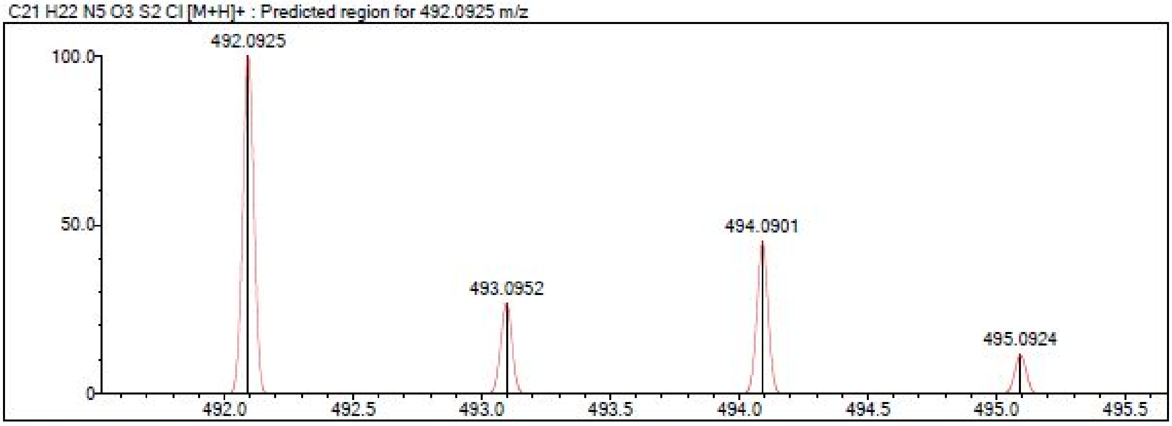

Rank	Score	Formula (M)	Ion	Meas. m/z	Pred. m/z	Df. (mDa)	Df. (ppm)	Iso	DBE
1	66.54	C21 H22 N5 O3 S2 Cl	[M+H]+	492.0912	492.0925	-1.3	-2.64	69.38	13.0

Figure S12. Mass spectrum of compound **6d**

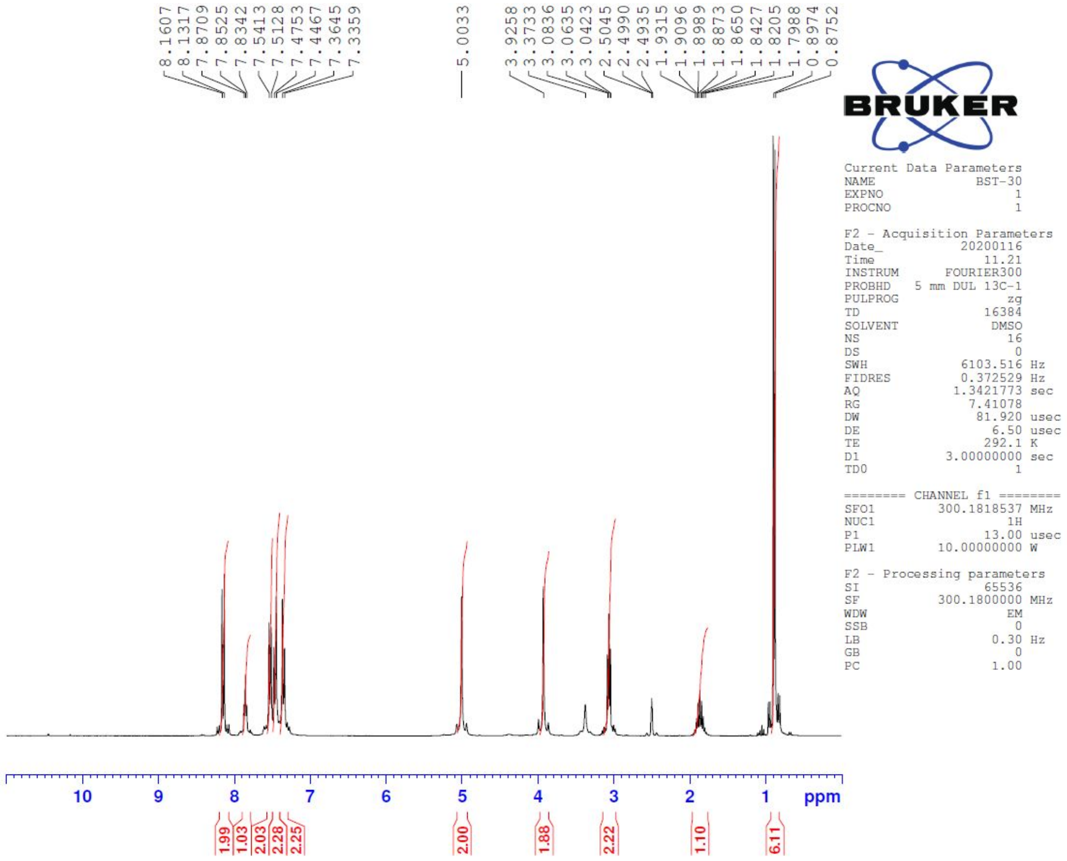

Figure S13. ^{1}H-NMR spectrum of compound **6e**

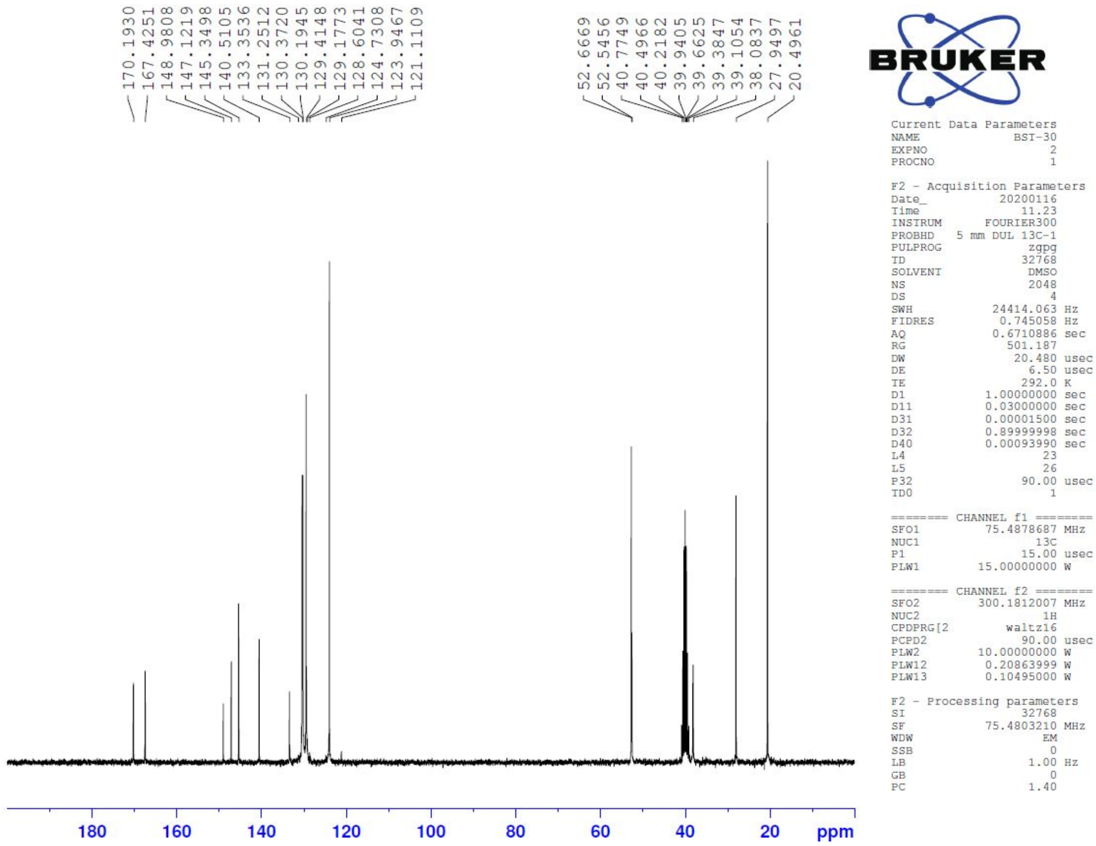

Figure S14. ^{13}C-NMR spectrum of compound **6e**

Formula Predictor Report - BST-30_19.lcd

Page 1 of 1

Data File: C:\LabSolutions\Data\Analiz\uac\BST-30_19.lcd

Elmt	Val.	Min	Max	Elmt	Val.	Min	Max	Elmt	Val.	Min	Max	Elmt	Val.	Min	Max	Use Adduct
H	1	6	30	O	2	3	4	S	2	2	3	Ru	2	0	0	H
C	4	8	30	F	1	0	1	Cl	1	0	1	Pd	2	0	0	
N	3	4	5	P	3	0	0	Br	1	0	0	I	3	0	0	

Error Margin (ppm): 5
HC Ratio: unlimited
Max Isotopes: 3
MSn Iso RI (%): 10.00

DBE Range: 7.0 - 20.0
Apply N Rule: yes
Isotope RI (%): 1.00
MSn Logic Mode: AND

Electron Ions: both
Use MSn Info: yes
Isotope Res: 9000
Max Results: 100

Event#: 1 MS(E+) Ret. Time : 3.613 -> 4.120 Scan# : 543 -> 619

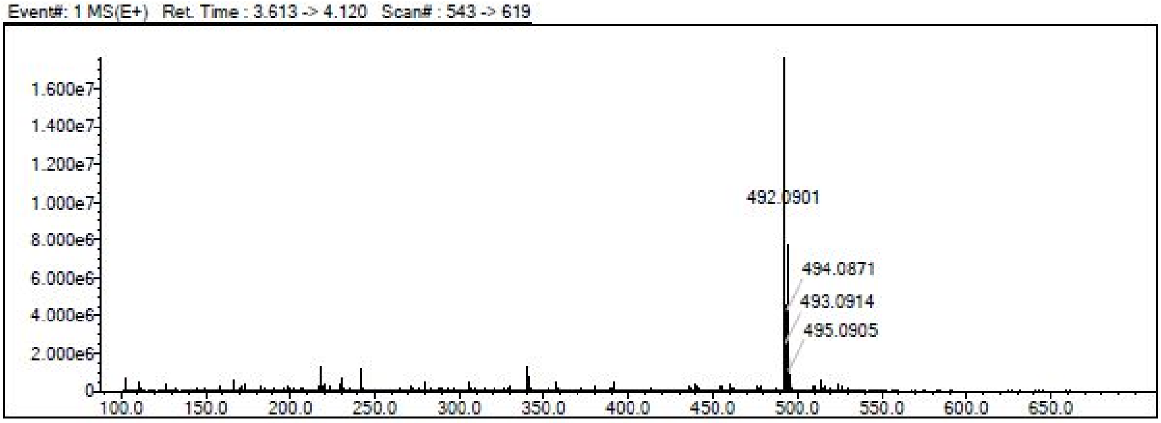

Measured region for 492.0901 m/z

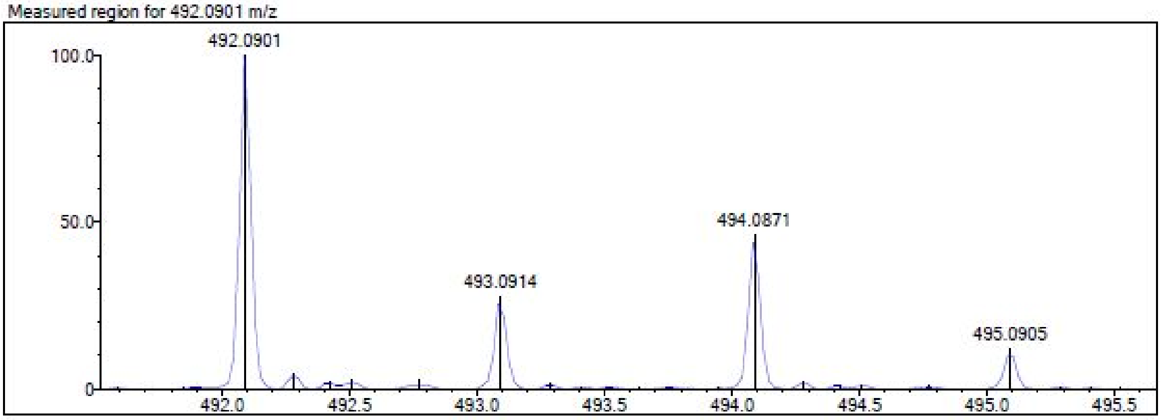

C21 H22 N5 O3 S2 Cl [M+H]+ : Predicted region for 492.0925 m/z

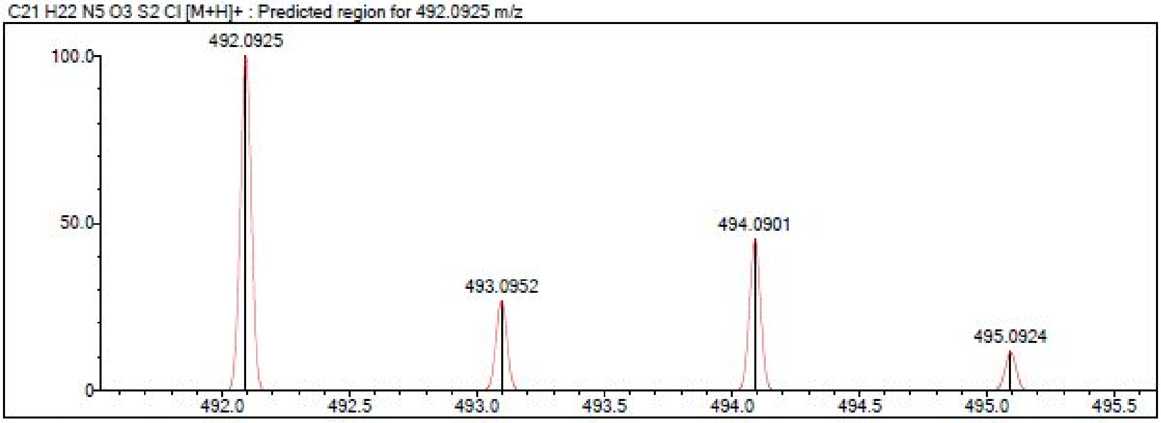

Rank	Score	Formula (M)	Ion	Meas. m/z	Pred. m/z	Df. (mDa)	Df. (ppm)	Iso	DBE
1	71.01	C21 H22 N5 O3 S2 Cl	[M+H]+	492.0901	492.0925	-2.4	-4.88	78.64	13.0

Figure S15. Mass spectrum of compound **6e**

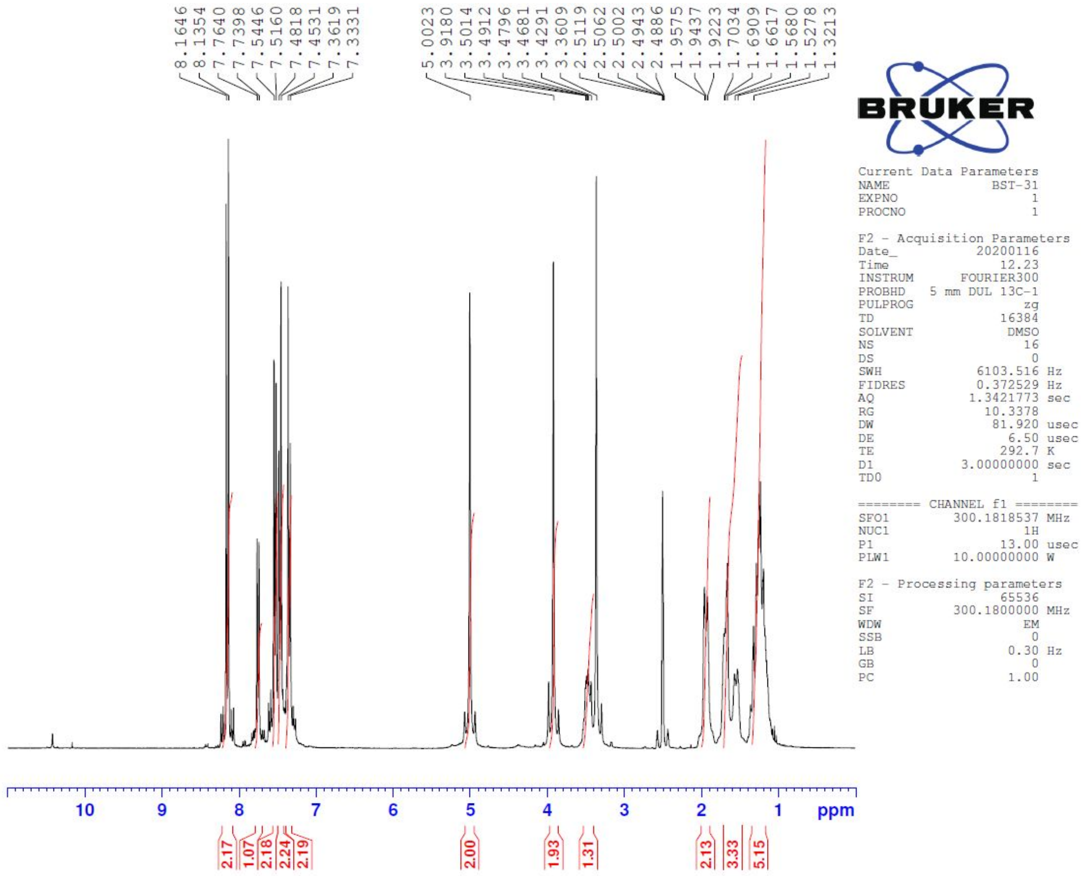

Figure S16. ^{1}H-NMR spectrum of compound **6f**

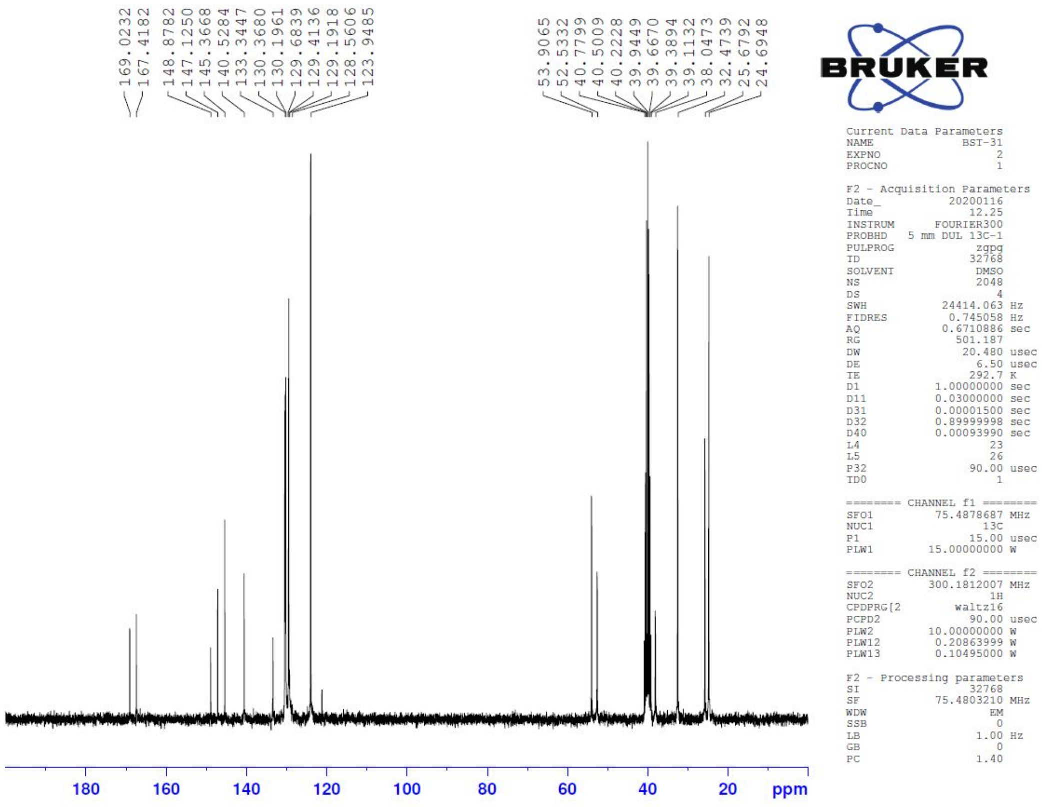

Figure S17. ^{13}C-NMR spectrum of compound **6f**

Data File: C:\LabSolutions\Data\Analiz\uac\BST-31_20.lcd

Elmt	Val.	Min	Max	Elmt	Val.	Min	Max	Elmt	Val.	Min	Max	Elmt	Val.	Min	Max	Use Adduct
H	1	6	30	O	2	3	4	S	2	2	3	Ru	2	0	0	H
C	4	8	30	F	1	0	1	Cl	1	0	1	Pd	2	0	0	
N	3	4	5	P	3	0	0	Br	1	0	0	I	3	0	0	

Error Margin (ppm): 5
HC Ratio: unlimited
Max Isotopes: 3
MSn Iso RI (%): 10.00

DBE Range: 7.0 - 20.0
Apply N Rule: yes
Isotope RI (%): 1.00
MSn Logic Mode: AND

Electron Ions: both
Use MSn Info: yes
Isotope Res: 9000
Max Results: 100

Event#: 1 MS(E+) Ret. Time : 4.027 -> 4.533 Scan# : 605 -> 681

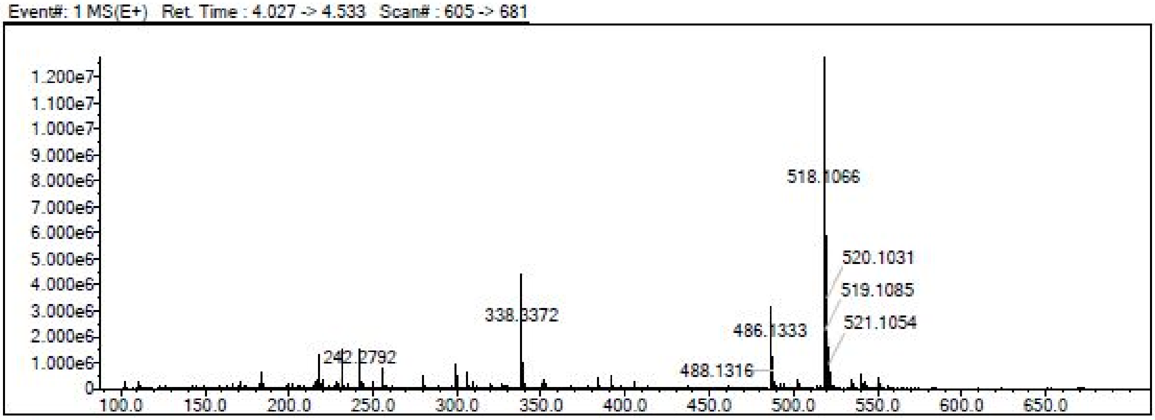

Measured region for 518.1066 m/z

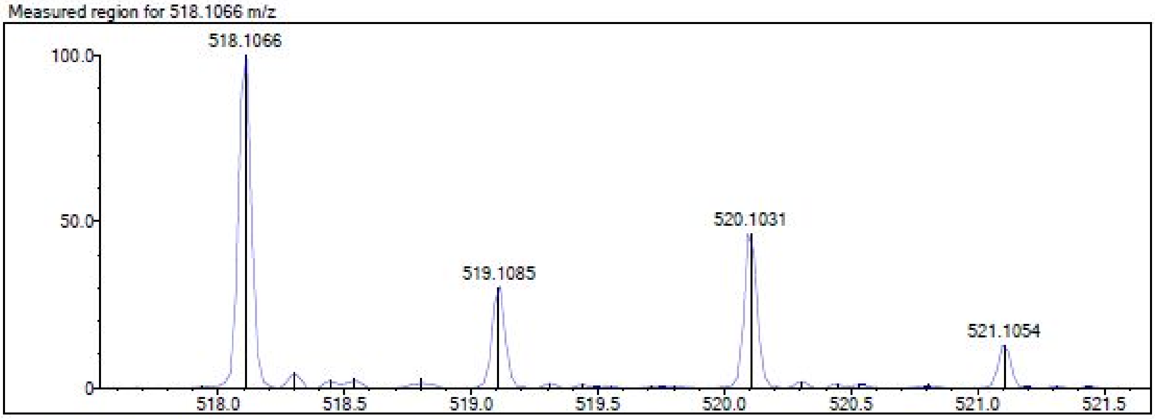

C23 H24 N5 O3 S2 Cl [M+H]+ : Predicted region for 518.1082 m/z

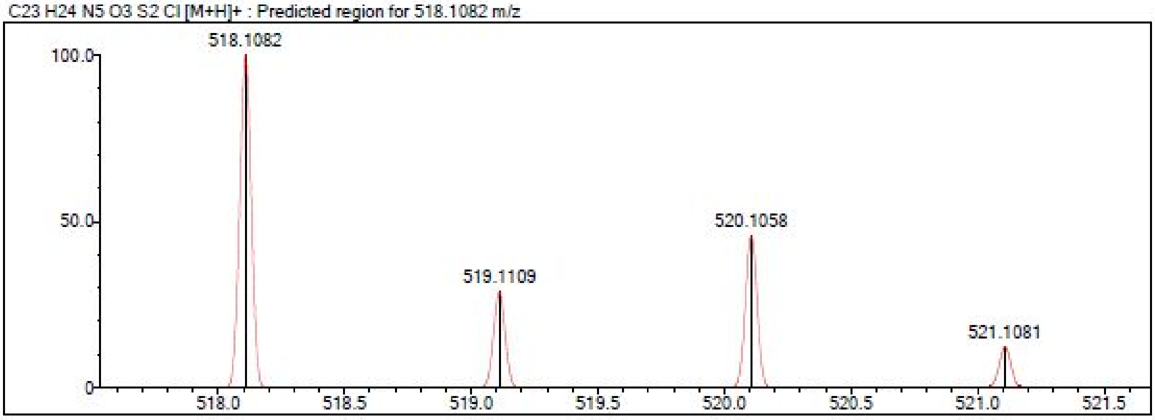

Rank	Score	Formula (M)	Ion	Meas. m/z	Pred. m/z	Df. (mDa)	Df. (ppm)	Iso	DBE
1	94.78	C23 H24 N5 O3 S2 Cl	[M+H]+	518.1066	518.1082	-1.6	-3.09	100.00	14.0

Figure S18. Mass spectrum of compound **6f**

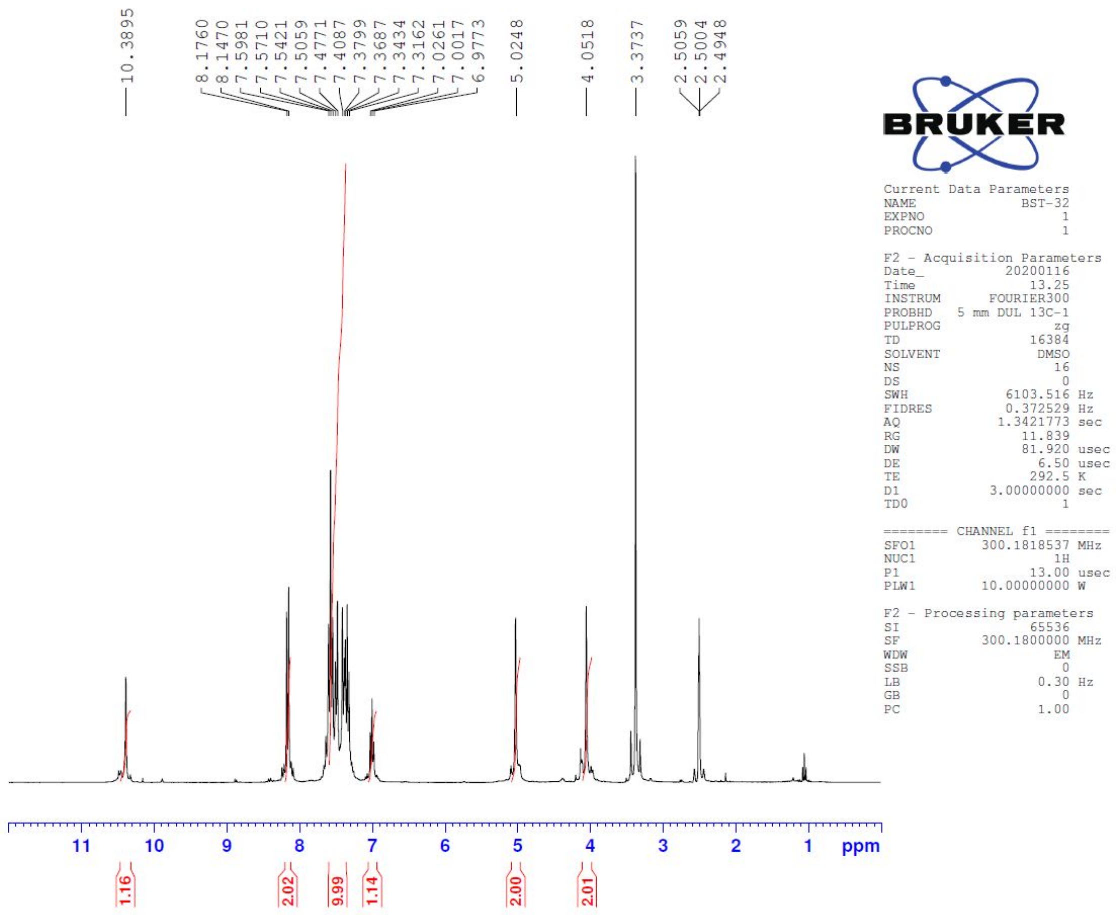

Figure S19. ^{1}H-NMR spectrum of compound **6g**

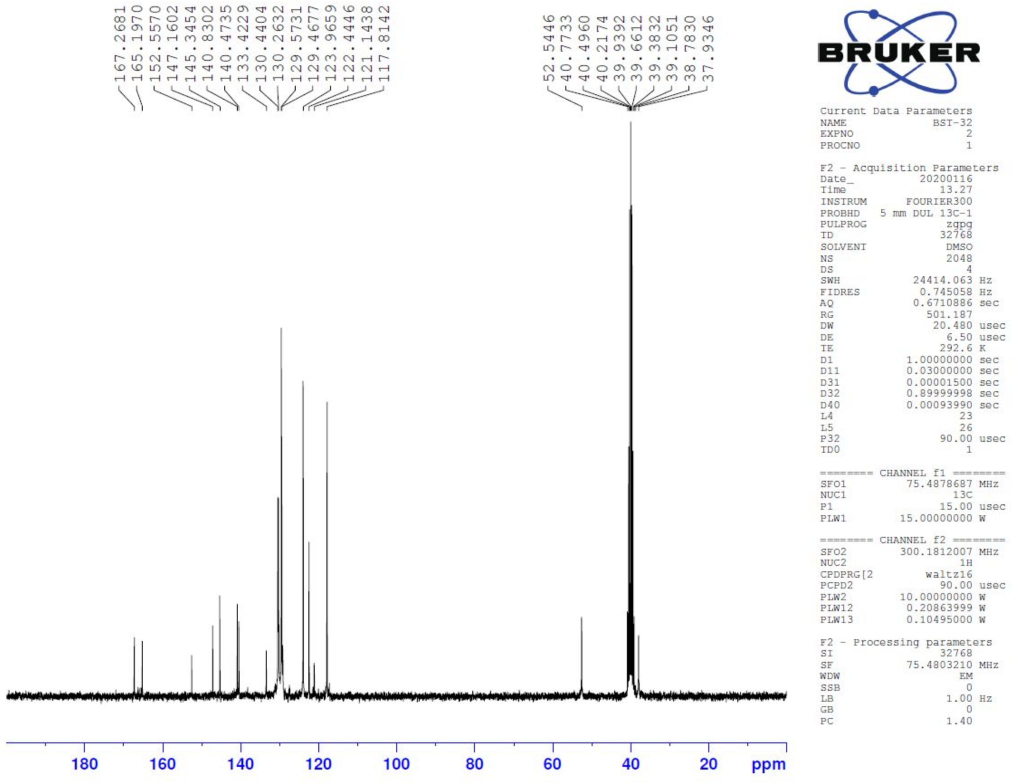

Figure S20. ^{13}C-NMR spectrum of compound **6g**

Data File: C:\LabSolutions\Data\Analiz\uac\BST-32_21.lcd

Elmt	Val.	Min	Max	Elmt	Val.	Min	Max	Elmt	Val.	Min	Max	Elmt	Val.	Min	Max	Use Adduct
H	1	6	30	O	2	3	4	S	2	2	3	Ru	2	0	0	H
C	4	8	30	F	1	0	1	Cl	1	0	1	Pd	2	0	0	
N	3	4	5	P	3	0	0	Br	1	0	0	I	3	0	0	

Error Margin (ppm):	5	DBE Range:	7.0 - 20.0	Electron Ions:	both
HC Ratio:	unlimited	Apply N Rule:	yes	Use MSn Info:	yes
Max Isotopes:	3	Isotope RI (%):	1.00	Isotope Res:	9000
MSn Iso RI (%):	10.00	MSn Logic Mode:	AND	Max Results:	100

Event#: 1 MS(E+) Ret. Time : 2.653 -> 3.187 Scan# : 399 -> 479

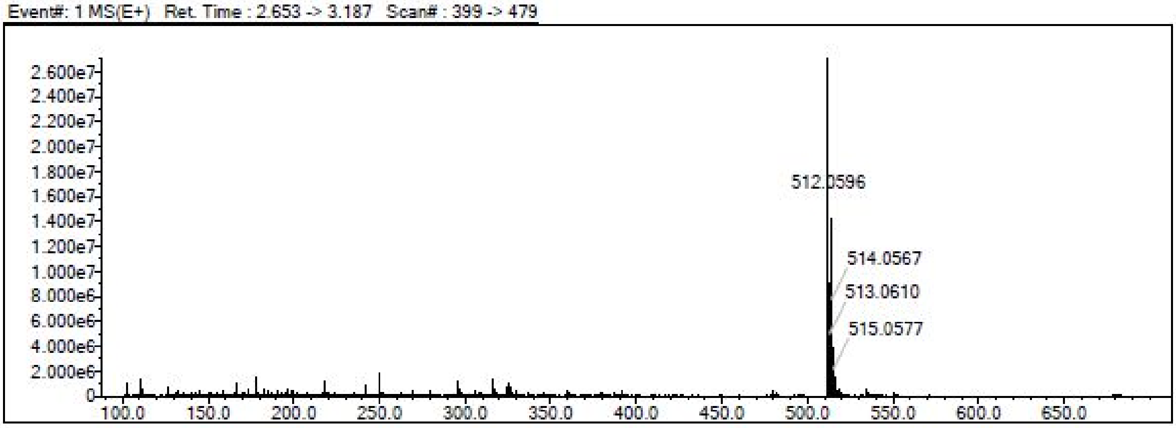

Measured region for 512.0596 m/z

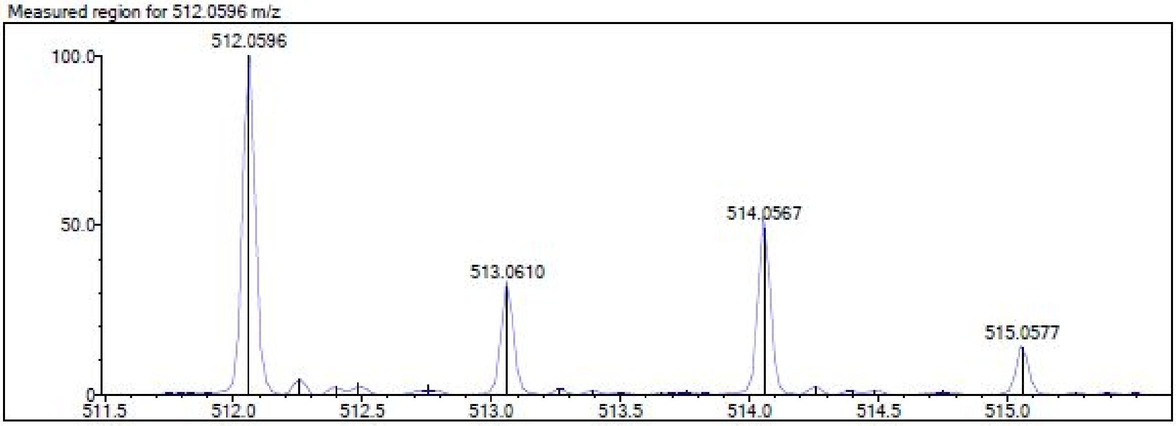

C23 H18 N5 O3 S2 Cl [M+H]+ : Predicted region for 512.0612 m/z

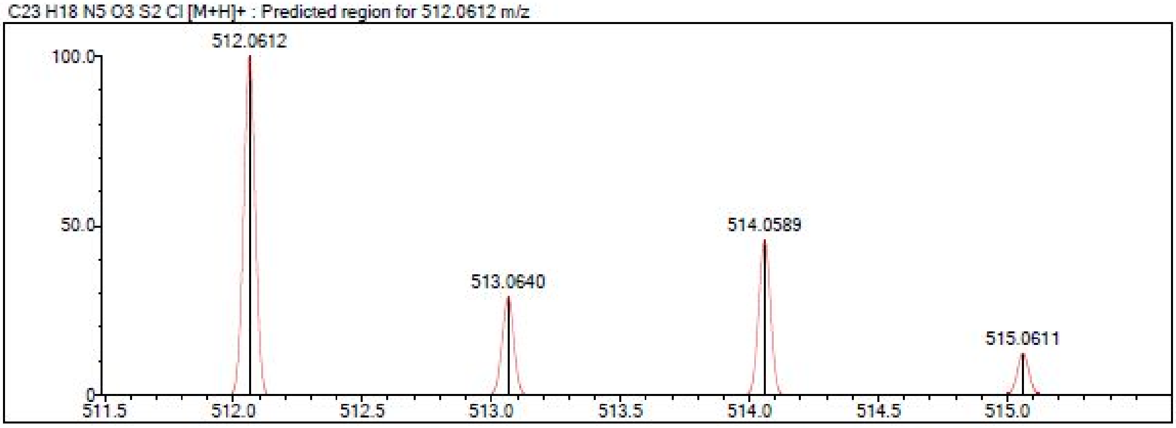

Rank	Score	Formula (M)	Ion	Meas. m/z	Pred. m/z	Df. (mDa)	Df. (ppm)	Iso	DBE
1	94.70	C23 H18 N5 O3 S2 Cl	[M+H]+	512.0596	512.0612	-1.6	-3.12	100.00	17.0

Figure S21. Mass spectrum of compound **6g**

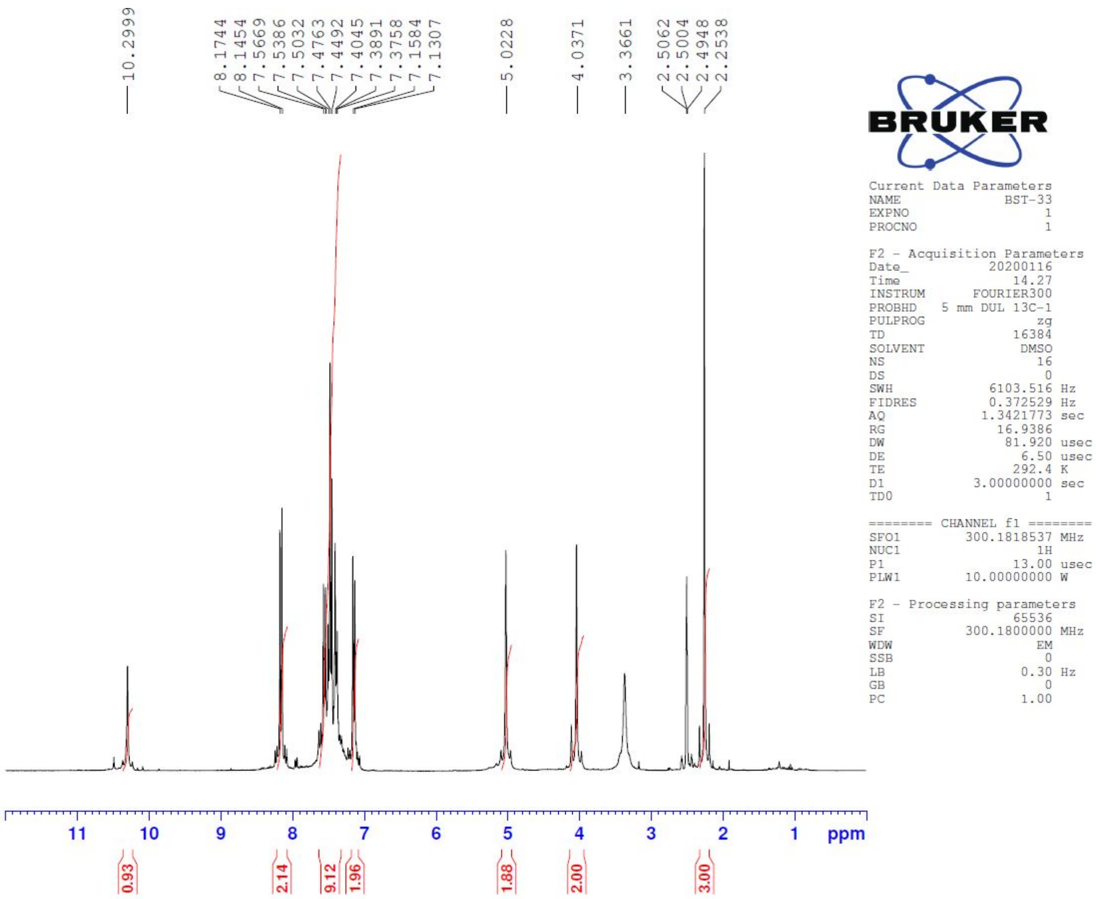

Figure S22. ^{1}H-NMR spectrum of compound **6h**

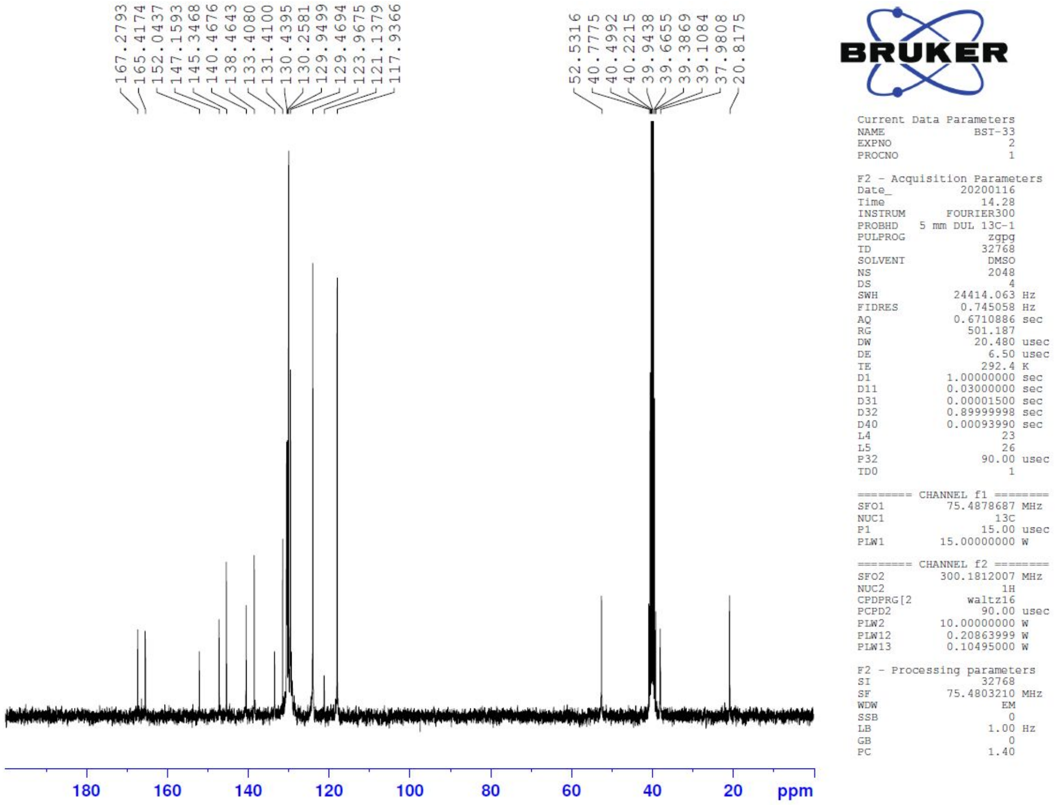

Figure S23. ^{13}C-NMR spectrum of compound **6h**

Data File: C:\LabSolutions\Data\Analiz\uac\BST-33_22.lcd

Elmt	Val.	Min	Max	Elmt	Val.	Min	Max	Elmt	Val.	Min	Max	Elmt	Val.	Min	Max	Use Adduct
H	1	6	30	O	2	3	4	S	2	2	3	Ru	2	0	0	H
C	4	8	30	F	1	0	1	Cl	1	0	1	Pd	2	0	0	
N	3	4	5	P	3	0	0	Br	1	0	0	I	3	0	0	

Error Margin (ppm): 5
HC Ratio: unlimited
Max Isotopes: 3
MSn Iso RI (%): 10.00

DBE Range: 14.0 - 20.0
Apply N Rule: yes
Isotope RI (%): 1.00
MSn Logic Mode: AND

Electron Ions: both
Use MSn Info: yes
Isotope Res: 9000
Max Results: 100

Event#: 1 MS(E+) Ret. Time : 3.760 -> 4.027 Scan# : 565 -> 605

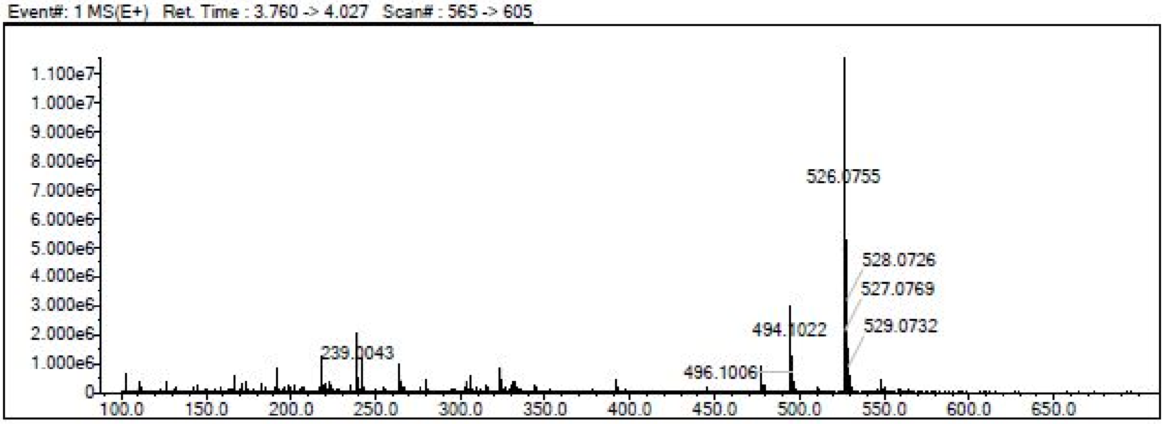

Measured region for 526.0755 m/z

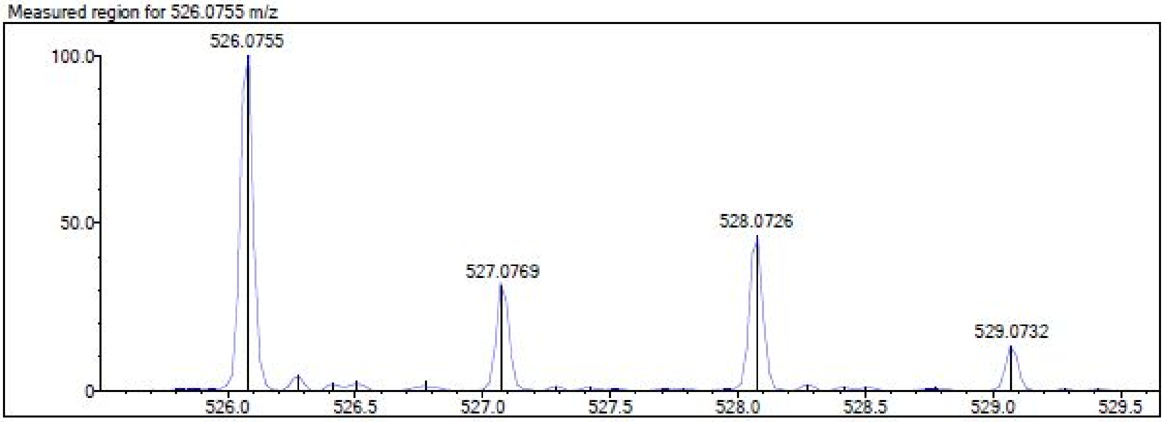

C24 H20 N5 O3 S2 Cl [M+H]+ : Predicted region for 526.0769 m/z

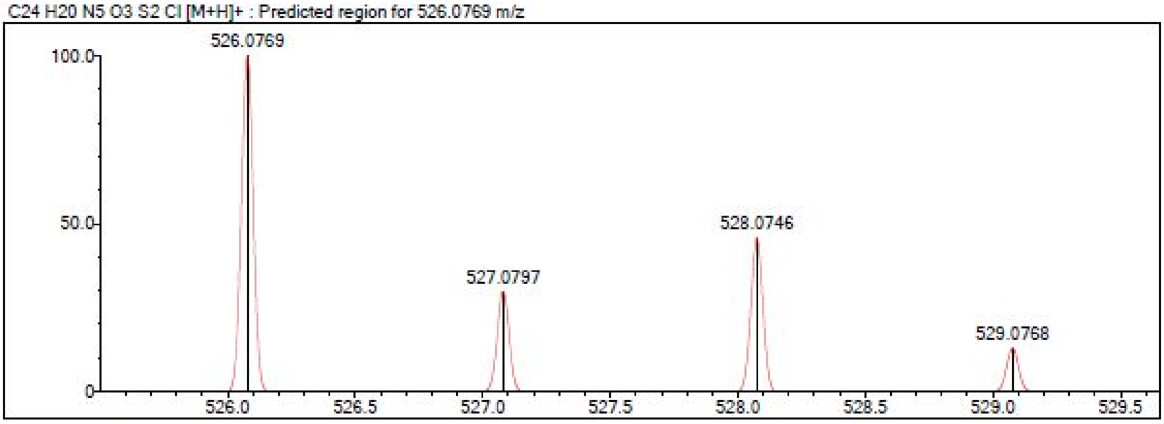

Rank	Score	Formula (M)	Ion	Meas. m/z	Pred. m/z	Df. (mDa)	Df. (ppm)	Iso	DBE
1	95.60	C24 H20 N5 O3 S2 Cl	[M+H]+	526.0755	526.0769	-1.4	-2.66	99.74	17.0

Figure S24. Mass spectrum of compound **6h**

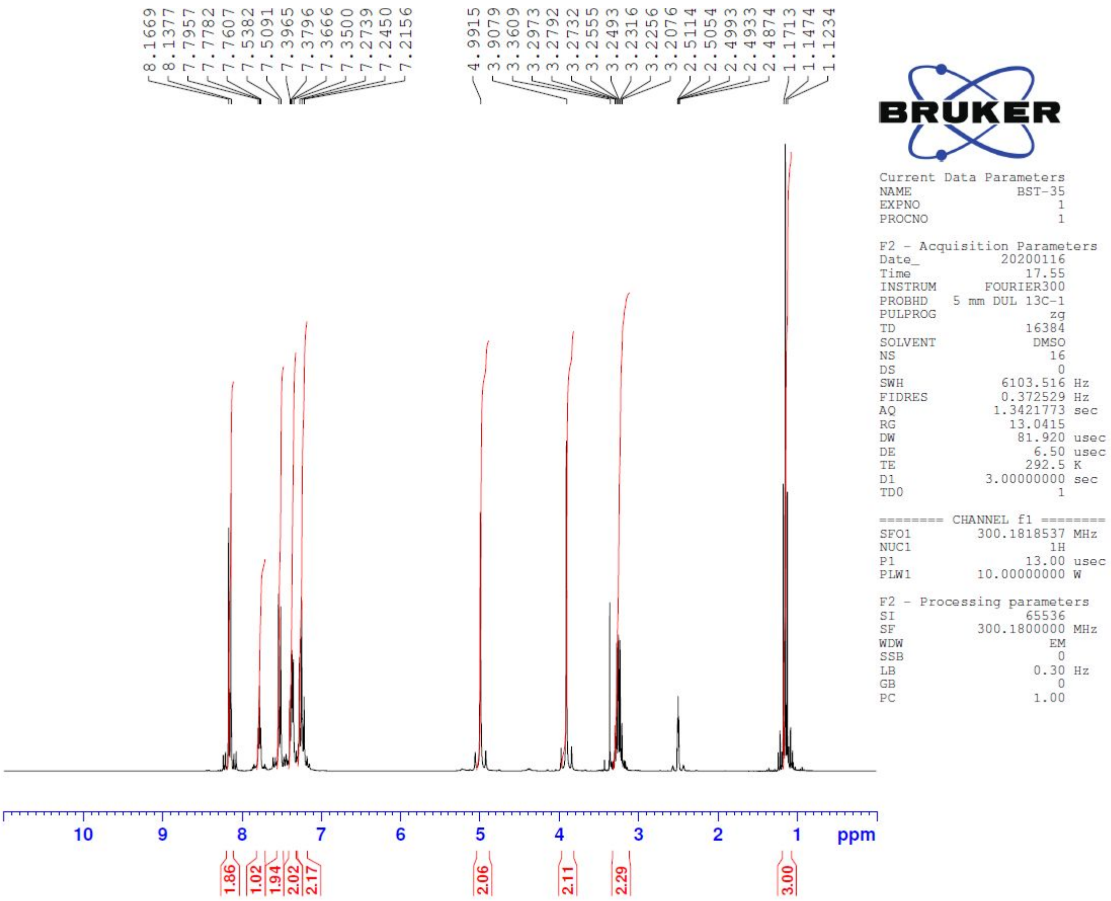

Figure S25. ^{1}H-NMR spectrum of compound **6i**

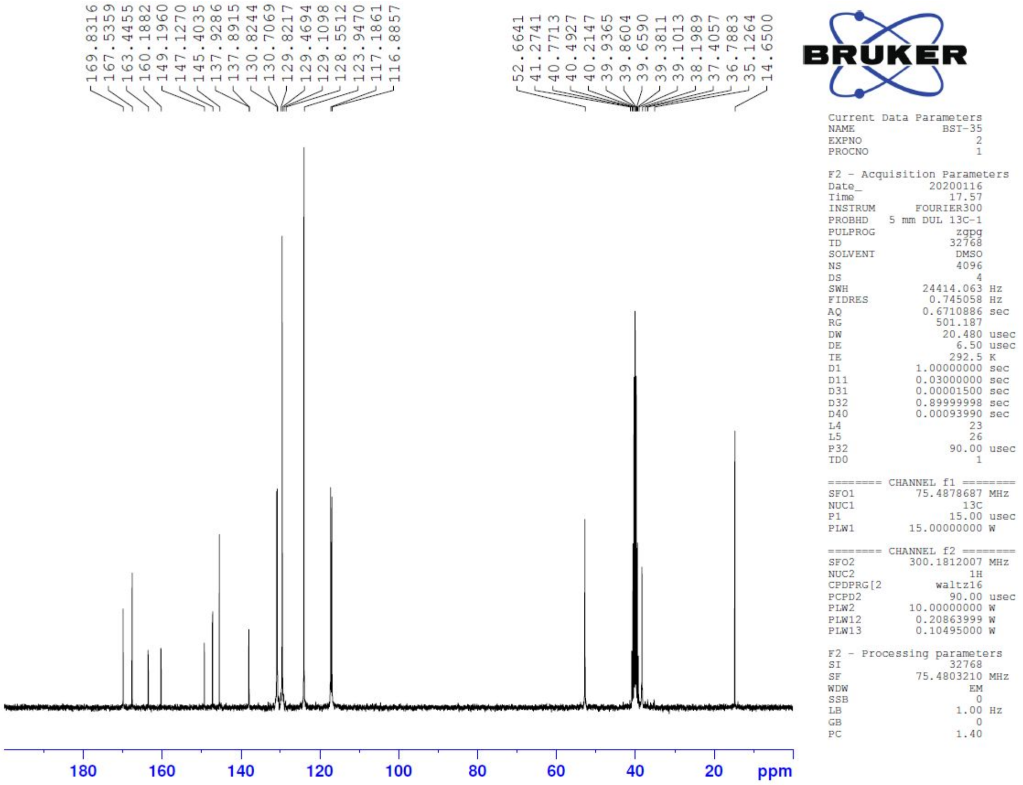

Figure S26. ^{13}C-NMR spectrum of compound **6i**

Data File: C:\LabSolutions\Data\Analiz\uac\BST-35_23.lcd

Elmt	Val.	Min	Max	Elmt	Val.	Min	Max	Elmt	Val.	Min	Max	Elmt	Val.	Min	Max	Use Adduct
H	1	6	30	O	2	3	4	S	2	2	3	Ru	2	0	0	H
C	4	8	30	F	1	0	1	Cl	1	0	1	Pd	2	0	0	
N	3	4	5	P	3	0	0	Br	1	0	0	I	3	0	0	

Error Margin (ppm): 15
HC Ratio: unlimited
Max Isotopes: 3
MSn Iso RI (%): 10.00

DBE Range: 10.0 - 20.0
Apply N Rule: yes
Isotope RI (%): 1.00
MSn Logic Mode: AND

Electron Ions: both
Use MSn Info: yes
Isotope Res: 9000
Max Results: 100

Event#: 1 MS(E+) Ret. Time : 2.973 Scan# : 447

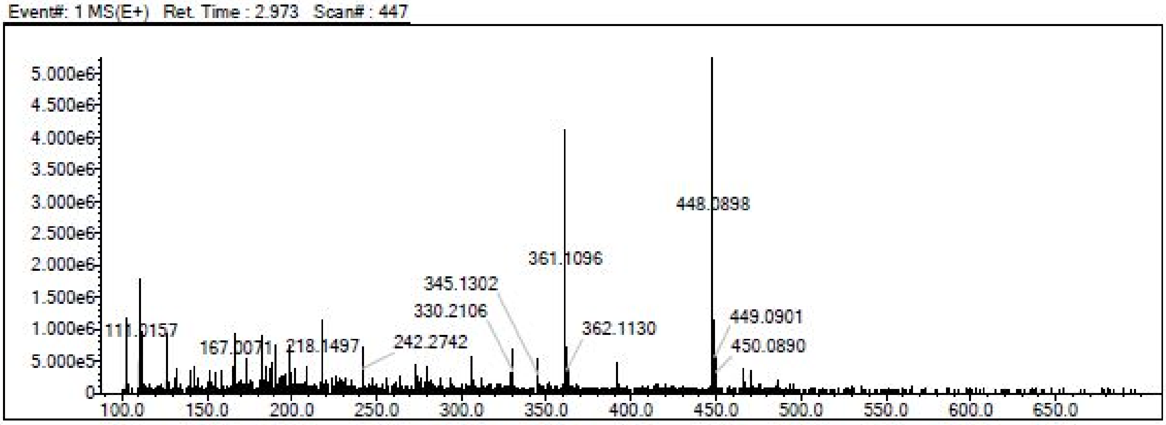

Measured region for 448.0898 m/z

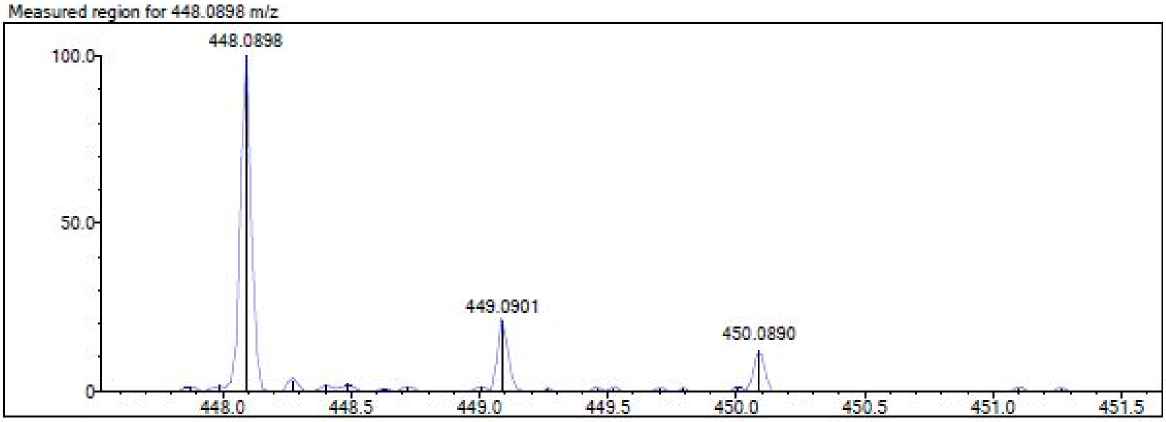

C19 H18 N5 O3 F S2 [M+H]+ : Predicted region for 448.0908 m/z

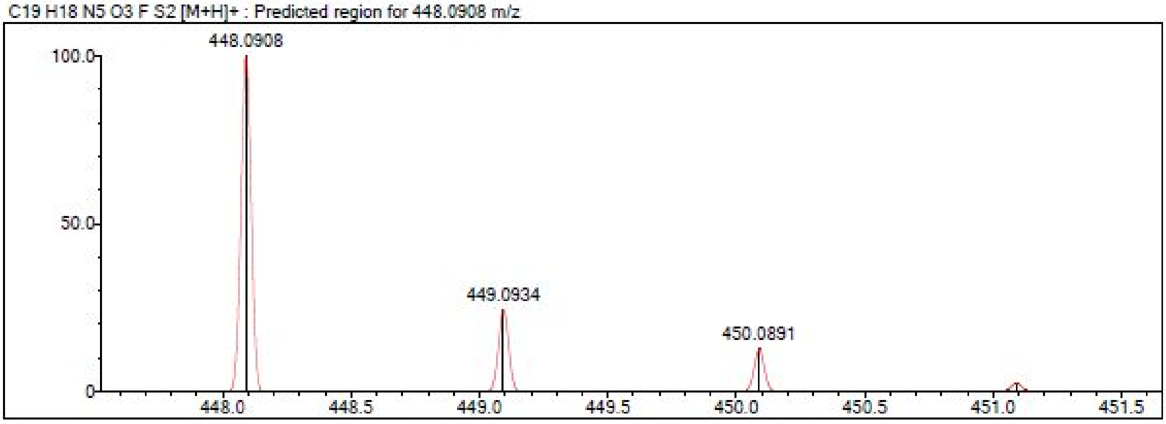

Rank	Score	Formula (M)	Ion	Meas. m/z	Pred. m/z	Df. (mDa)	Df. (ppm)	Iso	DBE
1	66.32	C19 H18 N5 O3 F S2	[M+H]+	448.0898	448.0908	-1.0	-2.23	68.42	13.0

Figure S27. Mass spectrum of compound **6i**

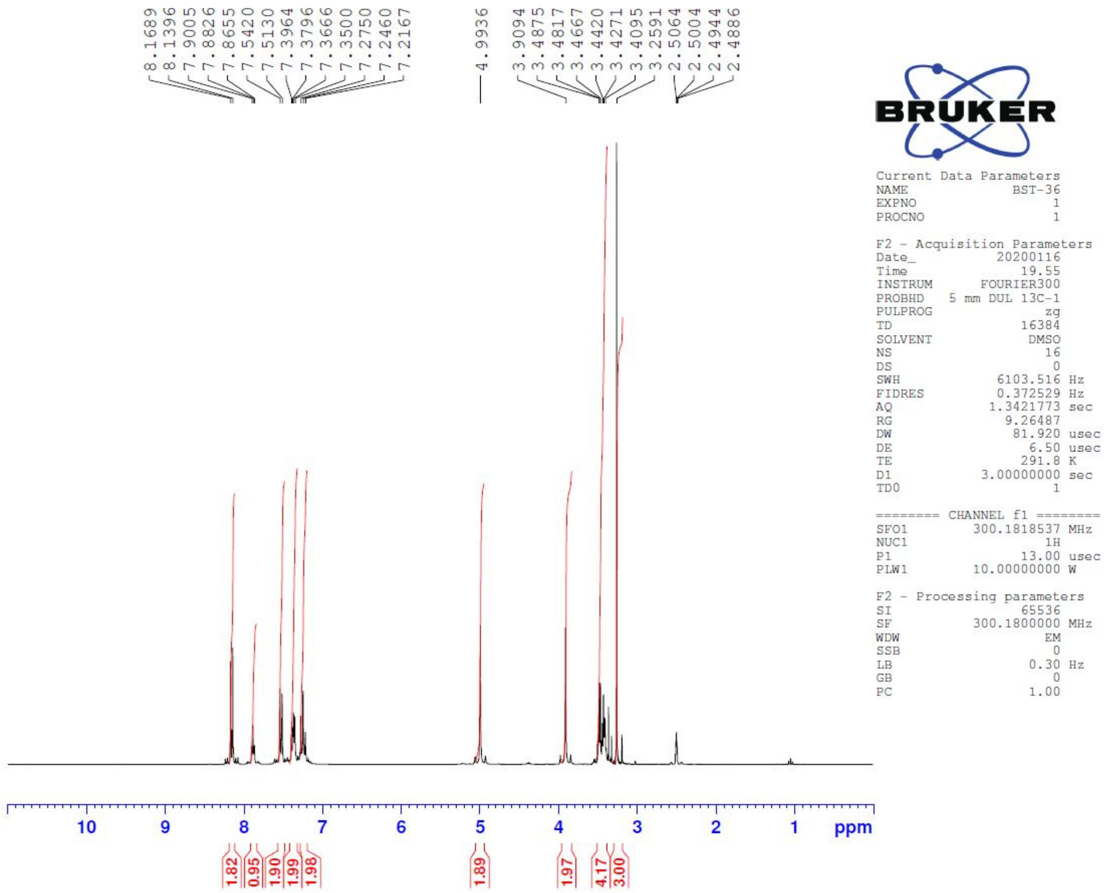

Figure S28. ^{1}H-NMR spectrum of compound **6j**

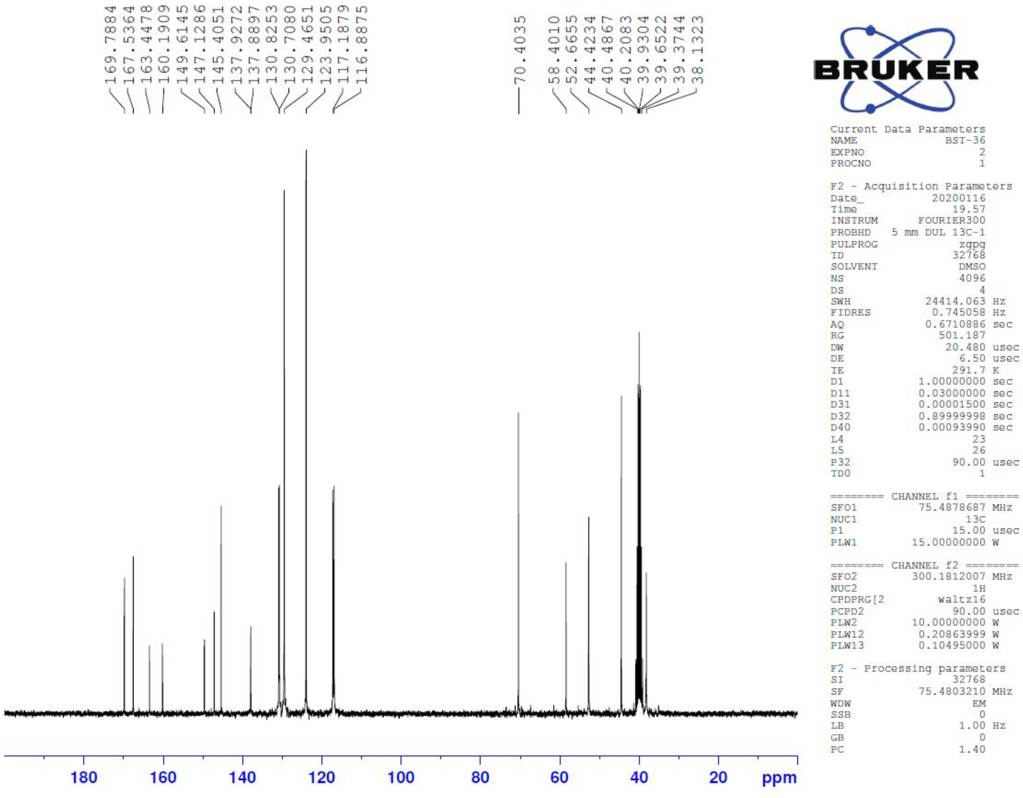

Figure S29. ^{13}C-NMR spectrum of compound **6j**

Data File: C:\LabSolutions\Data\Analiz\uac\BST-X_34.lcd

Elmt	Val.	Min	Max	Elmt	Val.	Min	Max	Elmt	Val.	Min	Max	Elmt	Val.	Min	Max	Use Adduct
H	1	6	30	O	2	3	4	S	2	2	3	Ru	2	0	0	H
C	4	8	30	F	1	0	1	Cl	1	0	1	Pd	2	0	0	
N	3	4	5	P	3	0	0	Br	1	0	0	I	3	0	0	

Error Margin (ppm):	5	DBE Range:	13.0 - 16.0	Electron Ions:	both
HC Ratio:	unlimited	Apply N Rule:	yes	Use MSn Info:	yes
Max Isotopes:	3	Isotope RI (%):	1.00	Isotope Res:	9000
MSn Iso RI (%):	10.00	MSn Logic Mode:	AND	Max Results:	100

Event#: 1 MS(E+) Ret. Time : 2.720 Scan# : 409

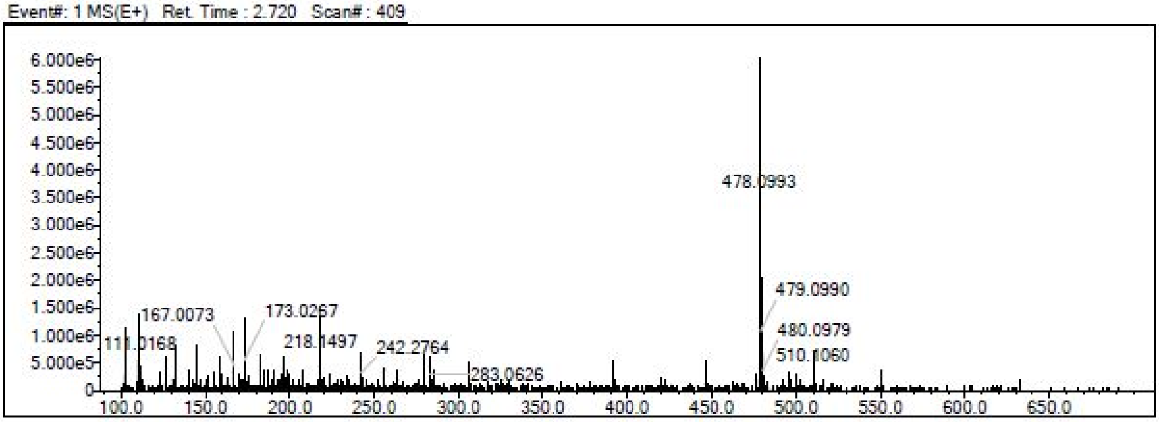

Measured region for 478.0993 m/z

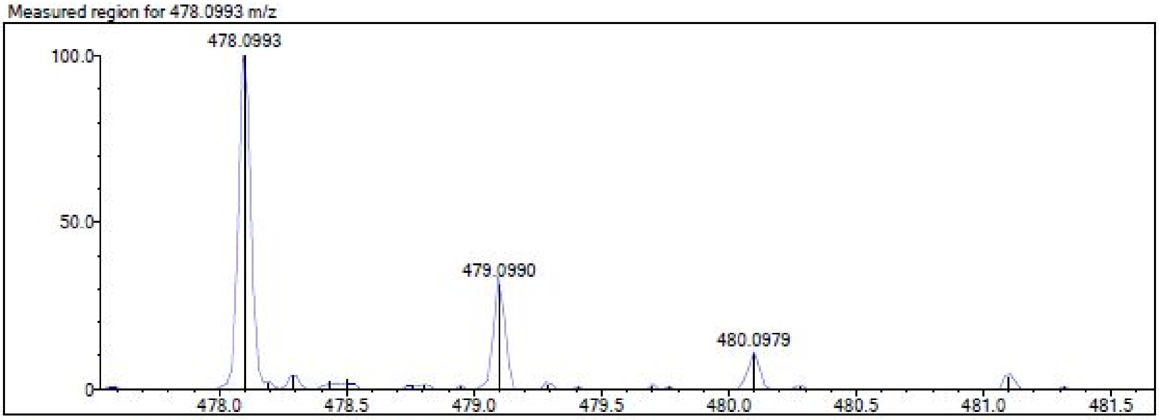

C20 H20 N5 O4 F S2 [M+H]+ : Predicted region for 478.1014 m/z

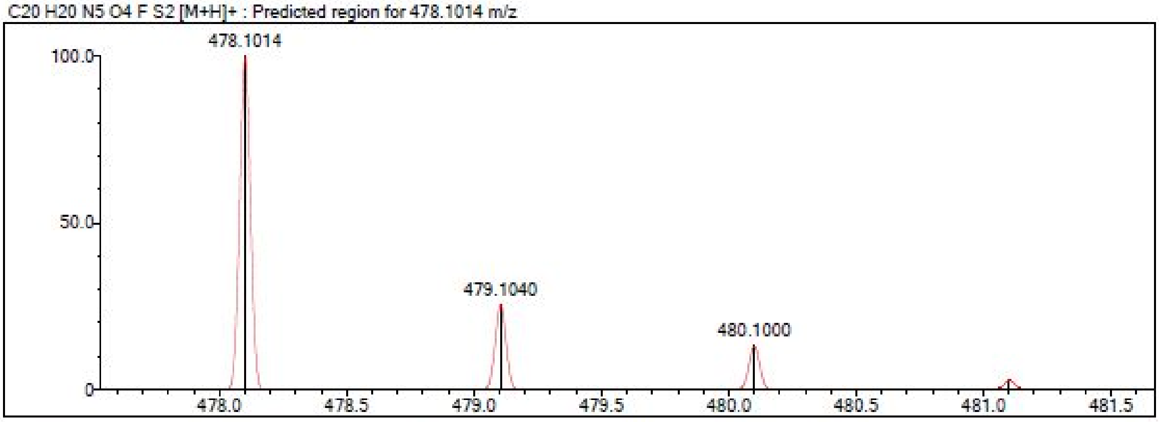

Rank	Score	Formula (M)	Ion	Meas. m/z	Pred. m/z	Df. (mDa)	Df. (ppm)	Iso	DBE
1	73.96	C20 H20 N5 O4 F S2	[M+H]+	478.0993	478.1014	-2.1	-4.39	80.81	13.0

Figure S30. Mass spectrum of compound **6j**

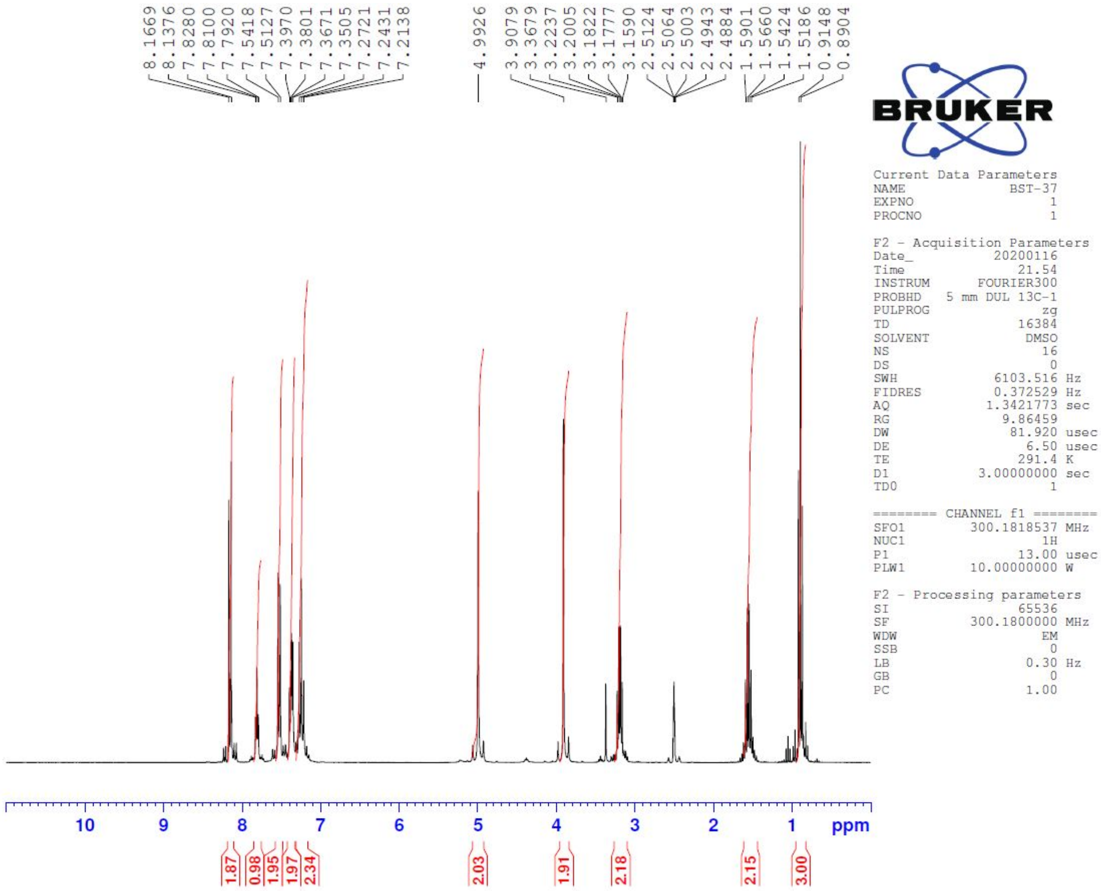

Figure S31. ^{1}H-NMR spectrum of compound **6k**

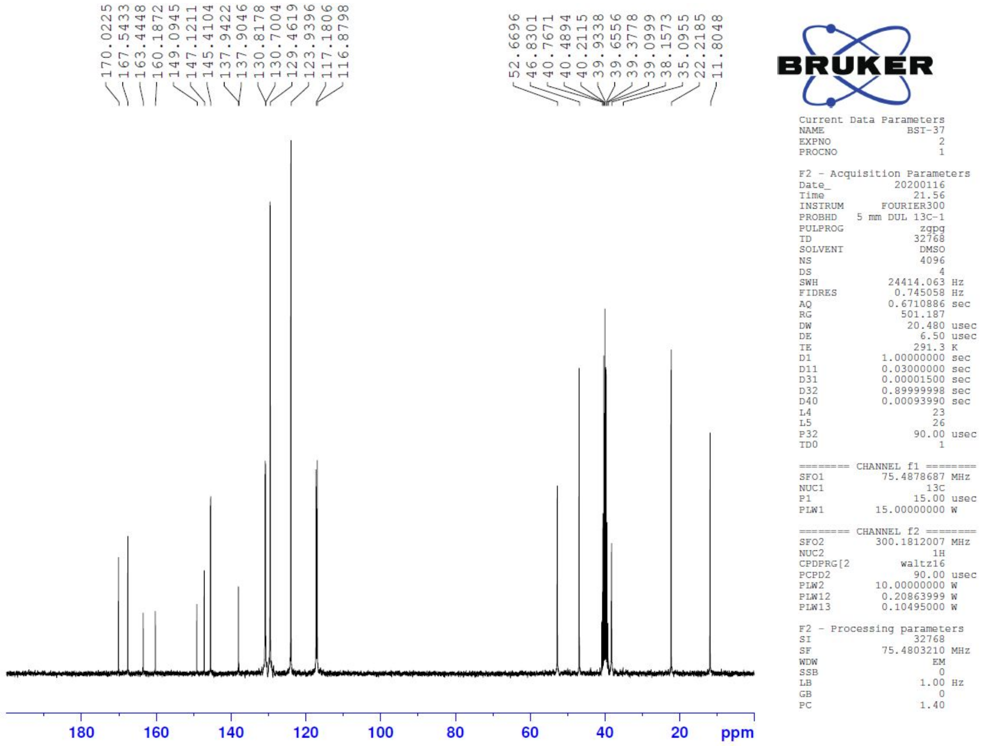

Figure S32. ^{13}C-NMR spectrum of compound **6k**

Data File: C:\LabSolutions\Data\Analiz\uac\BST-37_25.lcd

Elmt	Val.	Min	Max	Elmt	Val.	Min	Max	Elmt	Val.	Min	Max	Elmt	Val.	Min	Max	Use Adduct
H	1	6	30	O	2	3	4	S	2	2	3	Ru	2	0	0	H
C	4	8	30	F	1	0	1	Cl	1	0	1	Pd	2	0	0	
N	3	4	5	P	3	0	0	Br	1	0	0	I	3	0	0	

Error Margin (ppm): 15
HC Ratio: unlimited
Max Isotopes: 3
MSn Iso RI (%): 10.00

DBE Range: 10.0 - 20.0
Apply N Rule: yes
Isotope RI (%): 1.00
MSn Logic Mode: AND

Electron Ions: both
Use MSn Info: yes
Isotope Res: 9000
Max Results: 100

Event#: 1 MS(E+) Ret. Time : 2.960 Scan# : 445

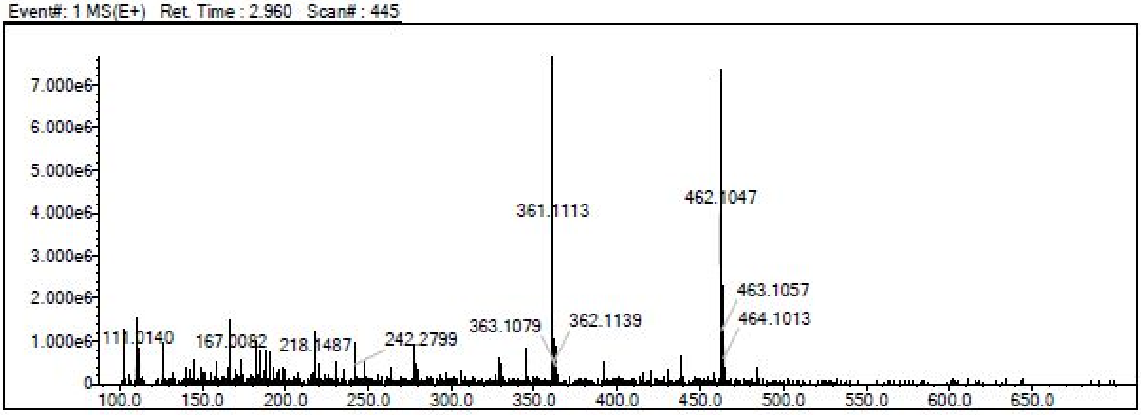

Measured region for 462.1047 m/z

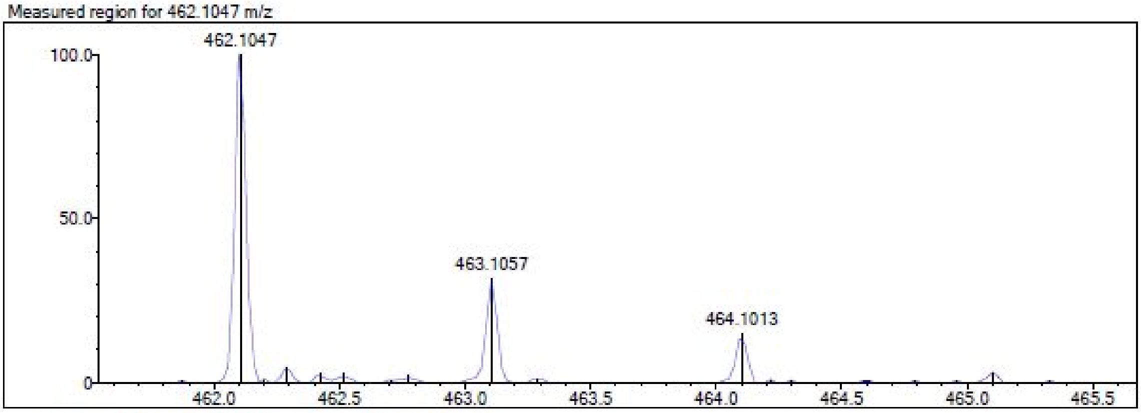

C20 H20 N5 O3 F S2 [M+H]+ : Predicted region for 462.1064 m/z

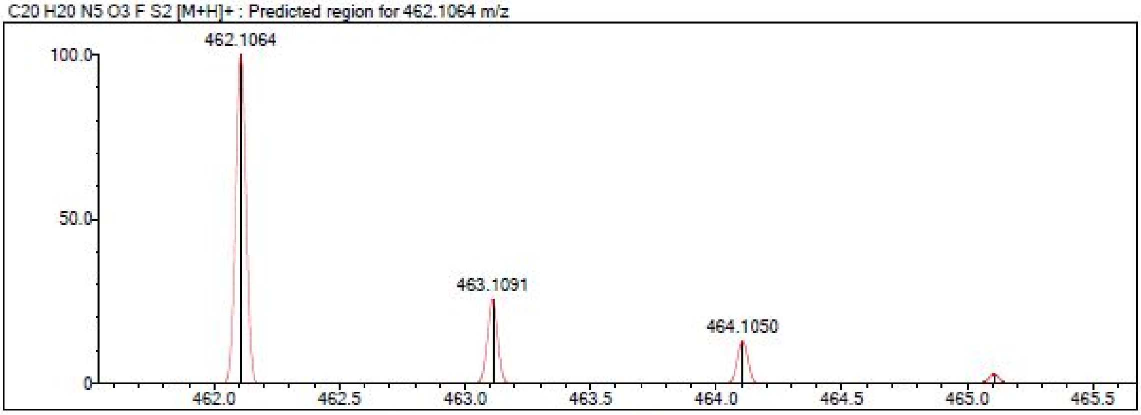

Rank	Score	Formula (M)	Ion	Meas. m/z	Pred. m/z	Df. (mDa)	Df. (ppm)	Iso	DBE
1	63.87	C20 H20 N5 O3 F S2	[M+H]+	462.1047	462.1064	-1.7	-3.68	68.46	13.0

Figure S33. Mass spectrum of compound **6k**

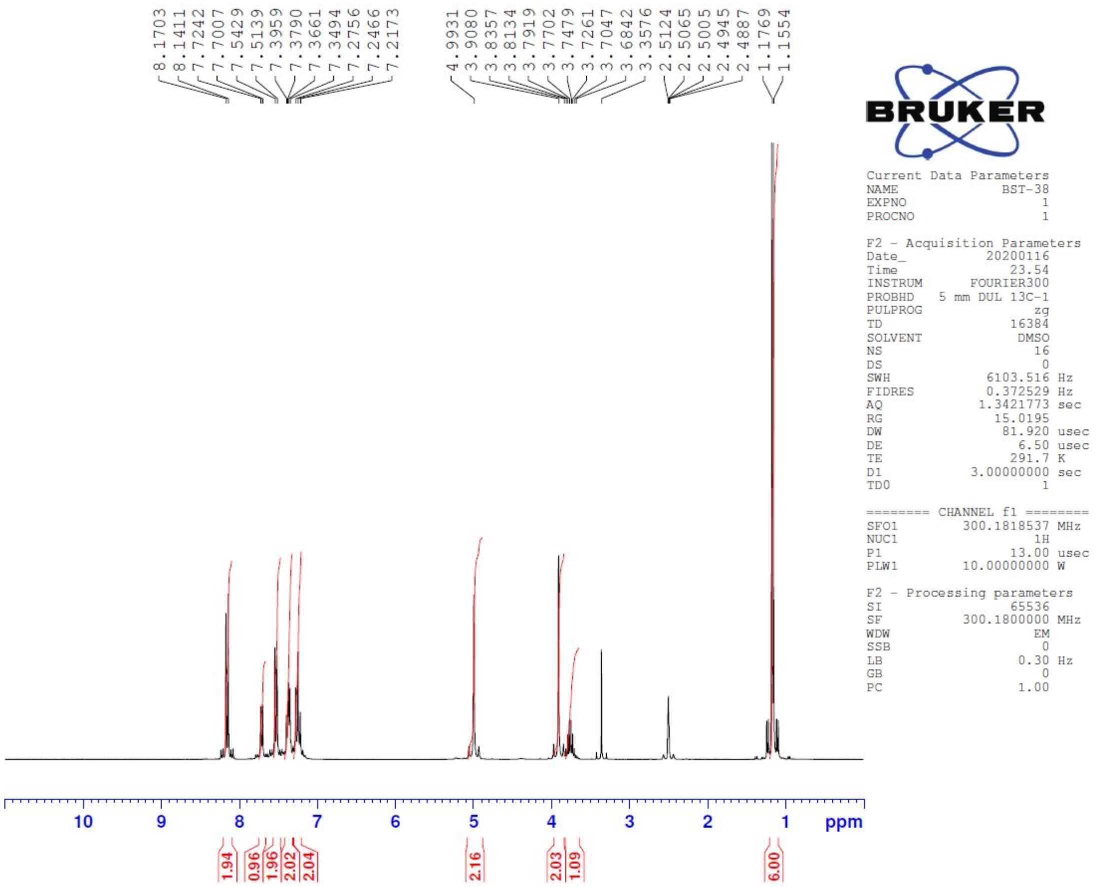

Figure S34. ^{1}H-NMR spectrum of compound **6l**

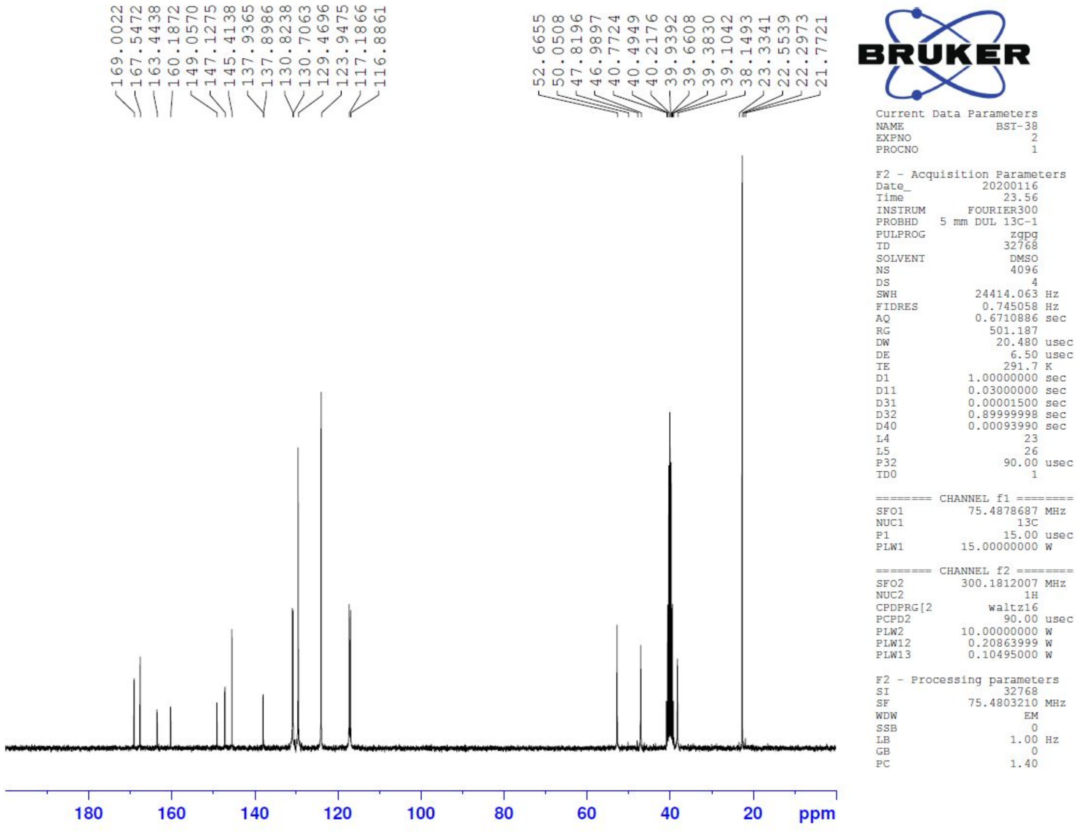

Figure S35. ^{13}C-NMR spectrum of compound **6l**

Data File: C:\LabSolutions\Data\Analiz\uac\BST-38A_26.lcd

Elmt	Val.	Min	Max	Elmt	Val.	Min	Max	Elmt	Val.	Min	Max	Elmt	Val.	Min	Max	Use Adduct
H	1	6	30	O	2	3	4	S	2	2	3	Ru	2	0	0	H
C	4	8	30	F	1	0	1	Cl	1	0	1	Pd	2	0	0	
N	3	4	5	P	3	0	0	Br	1	0	0	I	3	0	0	

Error Margin (ppm):	15	DBE Range:	10.0 - 20.0	Electron Ions:	both
HC Ratio:	unlimited	Apply N Rule:	yes	Use MSn Info:	yes
Max Isotopes:	3	Isotope RI (%):	1.00	Isotope Res:	9000
MSn Iso RI (%):	10.00	MSn Logic Mode:	AND	Max Results:	100

Event#: 1 MS(E+) Ret. Time : 2.827 Scan# : 425

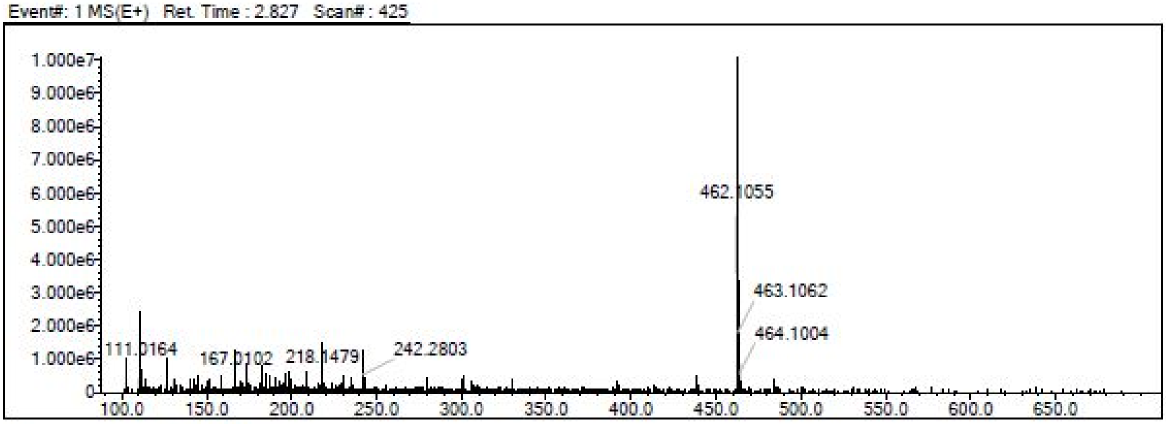

Measured region for 462.1055 m/z

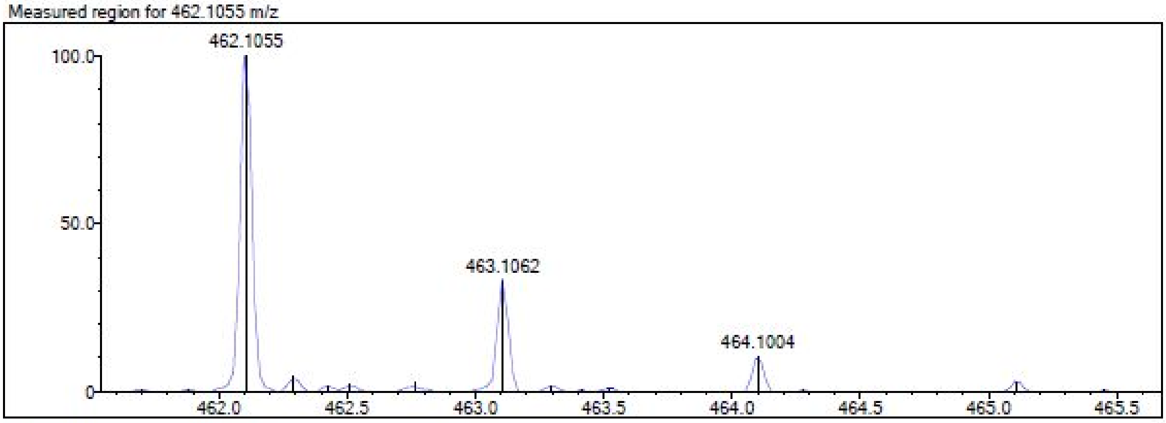

C20 H20 N5 O3 F S2 [M+H]+ : Predicted region for 462.1064 m/z

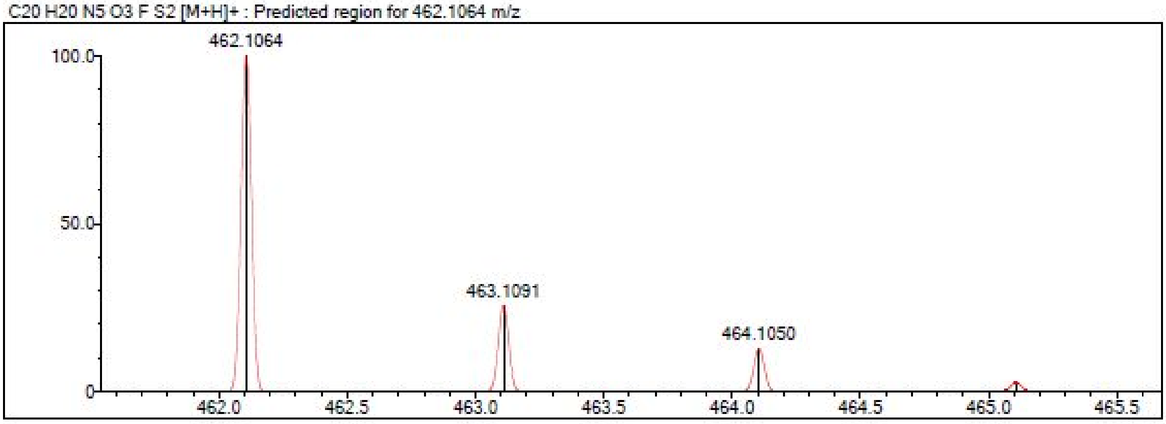

Rank	Score	Formula (M)	Ion	Meas. m/z	Pred. m/z	Df. (mDa)	Df. (ppm)	Iso	DBE
1	78.15	C20 H20 N5 O3 F S2	[M+H]+	462.1055	462.1064	-0.9	-1.95	80.06	13.0

Figure S36. Mass spectrum of compound **6l**

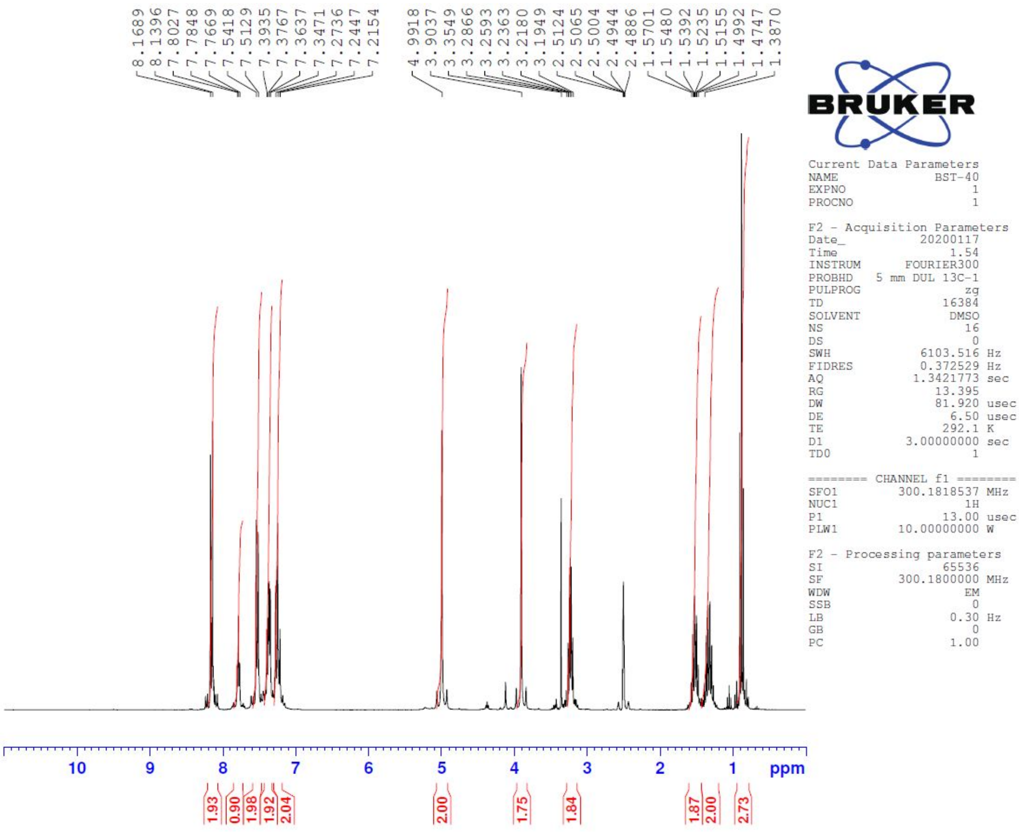

Figure S37. ^{1}H-NMR spectrum of compound **6m**

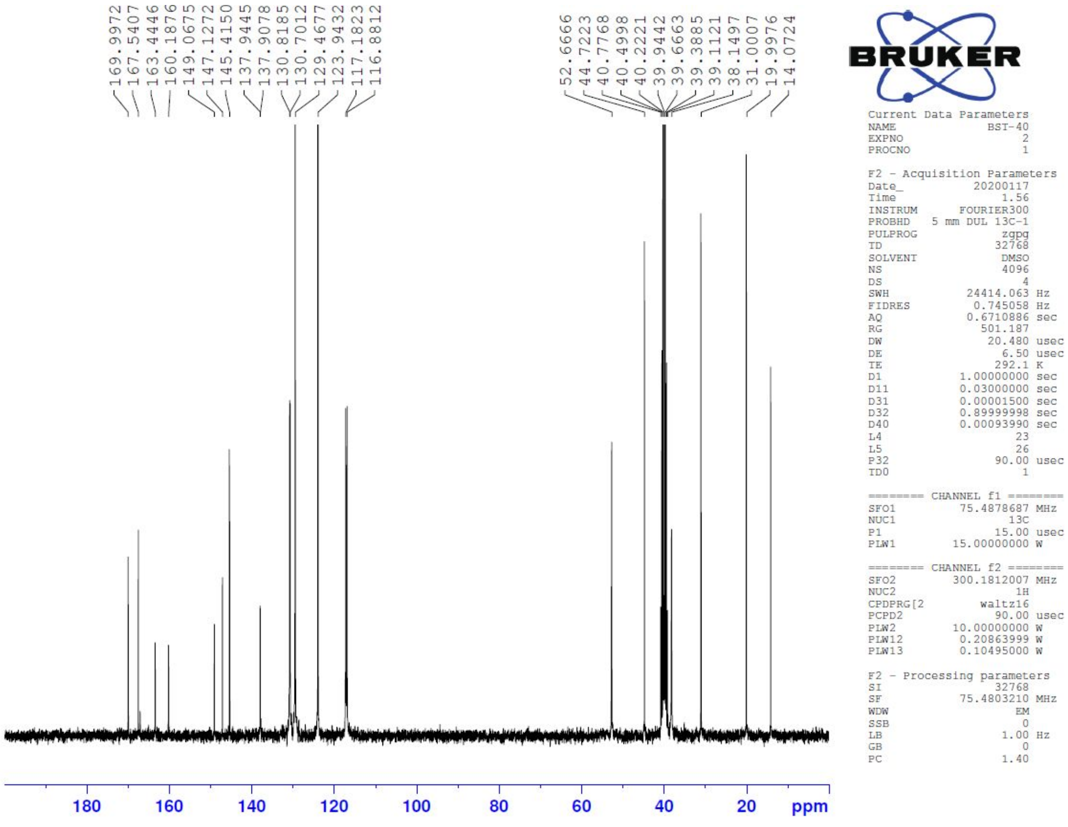

Figure S38. ^{13}C-NMR spectrum of compound **6m**

Data File: C:\LabSolutions\Data\Analiz\uac\BST-40_28.lcd

Elmt	Val.	Min	Max	Elmt	Val.	Min	Max	Elmt	Val.	Min	Max	Elmt	Val.	Min	Max	Use Adduct
H	1	6	30	O	2	3	4	S	2	2	3	Ru	2	0	0	H
C	4	8	30	F	1	0	1	Cl	1	0	1	Pd	2	0	0	
N	3	4	5	P	3	0	0	Br	1	0	0	I	3	0	0	

Error Margin (ppm): 15
HC Ratio: unlimited
Max Isotopes: 3
MSn Iso RI (%): 10.00

DBE Range: 10.0 - 20.0
Apply N Rule: yes
Isotope RI (%): 1.00
MSn Logic Mode: AND

Electron Ions: both
Use MSn Info: yes
Isotope Res: 9000
Max Results: 100

Event#: 1 MS(E+) Ret. Time : 3.027 -> 3.533 Scan# : 455 -> 531

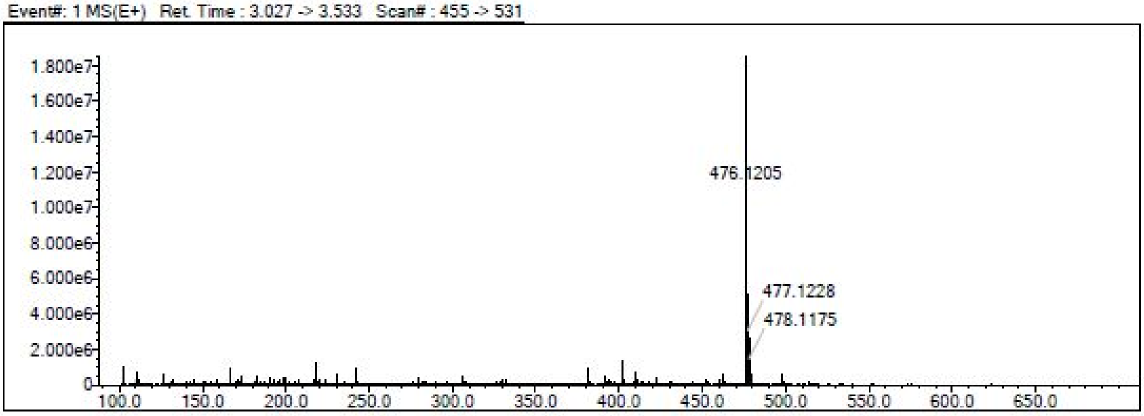

Measured region for 476.1205 m/z

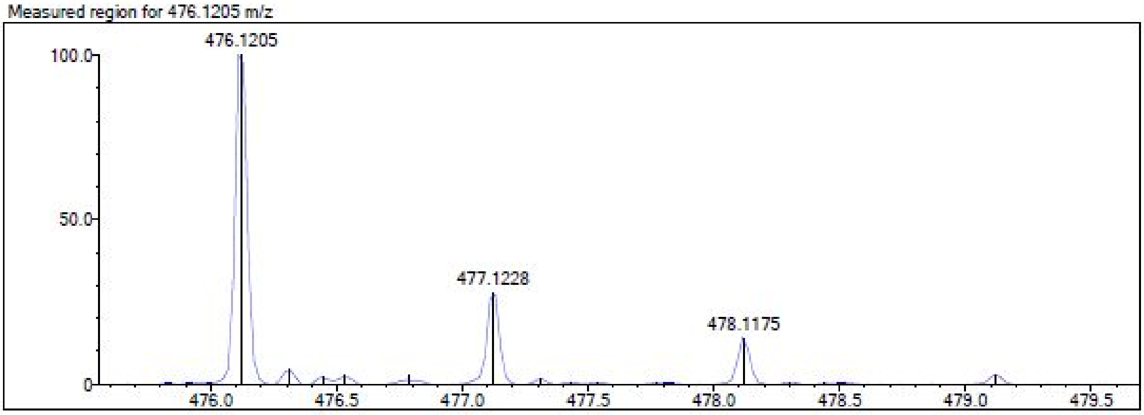

C21 H22 N5 O3 F S2 [M+H]+ : Predicted region for 476.1221 m/z

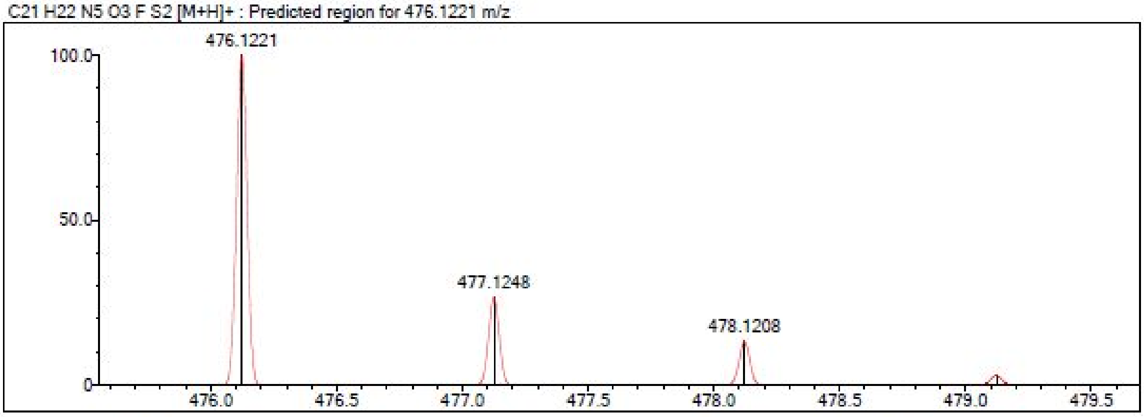

Rank	Score	Formula (M)	Ion	Meas. m/z	Pred. m/z	Df. (mDa)	Df. (ppm)	Iso	DBE
1	94.10	C21 H22 N5 O3 F S2	[M+H]+	476.1205	476.1221	-1.6	-3.36	100.00	13.0

Figure S39. Mass spectrum of compound **6m**

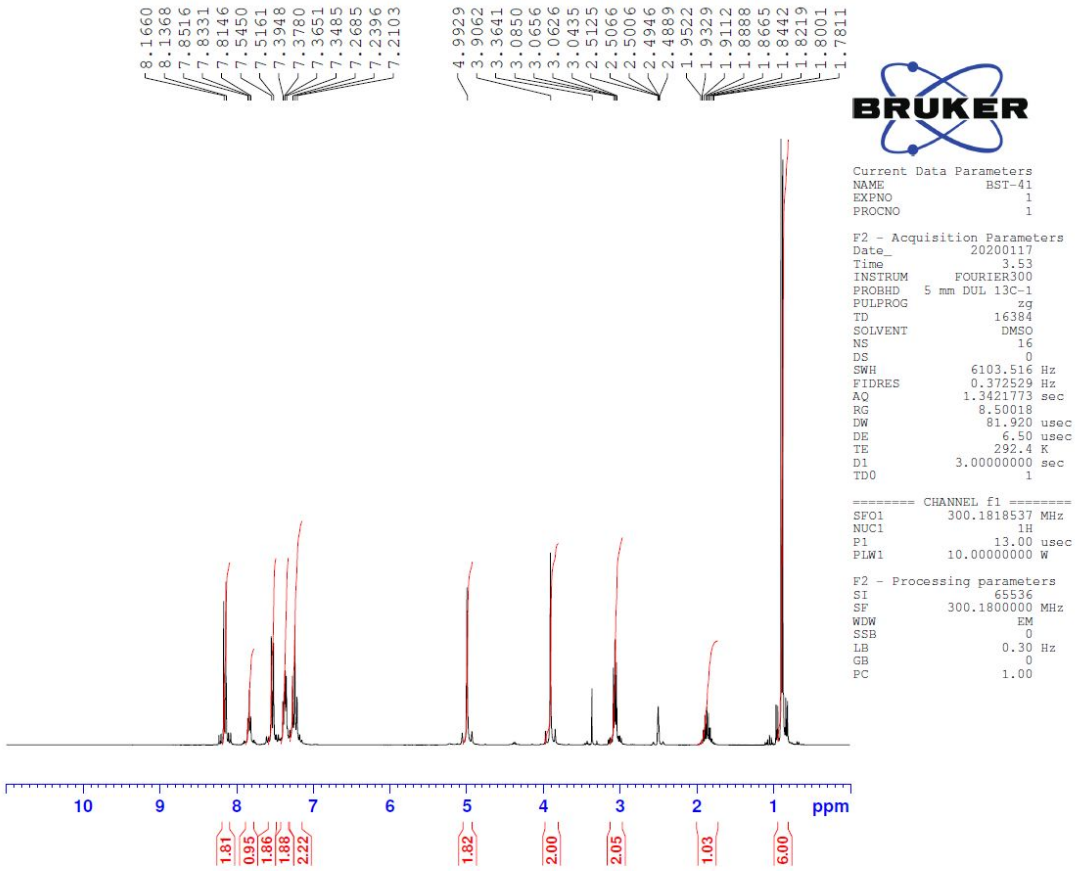

Figure S40. ^{1}H-NMR spectrum of compound **6n**

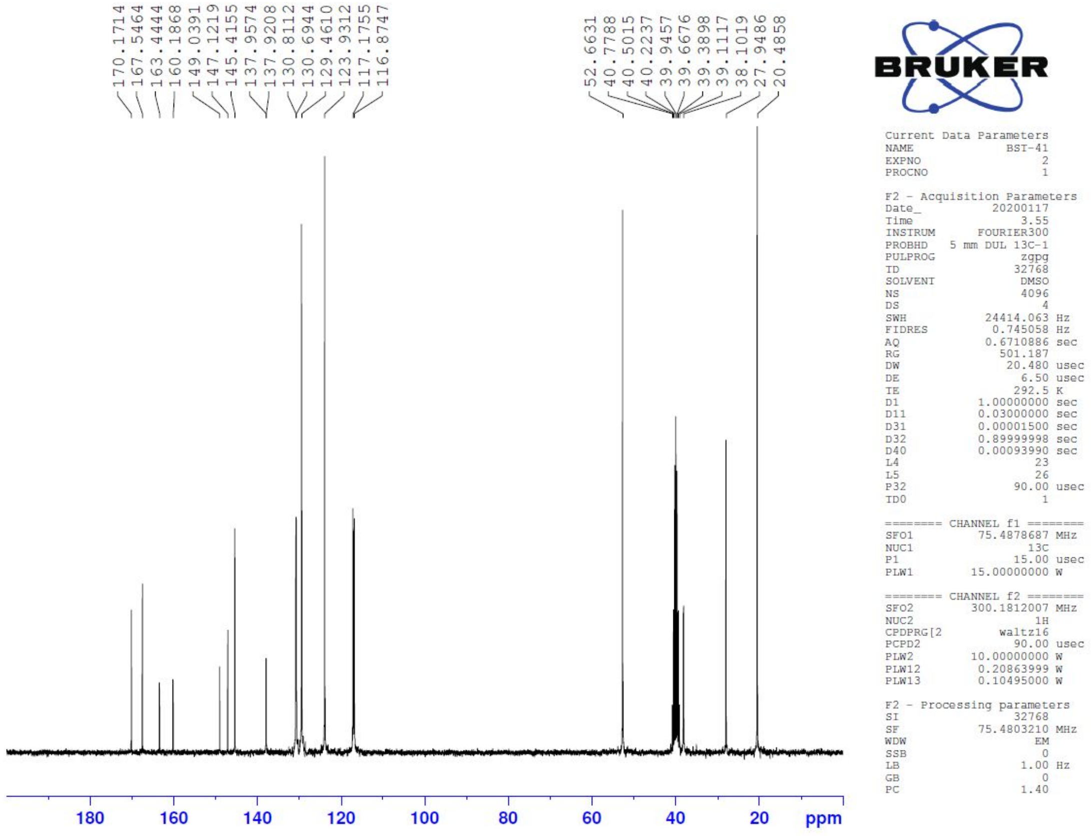

Figure S41. ^{13}C-NMR spectrum of compound **6n**

Data File: C:\LabSolutions\Data\Analiz\uac\BST-41_29.lcd

Elmt	Val.	Min	Max	Elmt	Val.	Min	Max	Elmt	Val.	Min	Max	Elmt	Val.	Min	Max	Use Adduct
H	1	6	30	O	2	3	4	S	2	2	3	Ru	2	0	0	H
C	4	8	30	F	1	0	1	Cl	1	0	1	Pd	2	0	0	
N	3	4	5	P	3	0	0	Br	1	0	0	I	3	0	0	

Error Margin (ppm):	15	DBE Range:	10.0 - 20.0	Electron Ions:	both
HC Ratio:	unlimited	Apply N Rule:	yes	Use MSn Info:	yes
Max Isotopes:	3	Isotope RI (%):	1.00	Isotope Res:	9000
MSn Iso RI (%):	10.00	MSn Logic Mode:	AND	Max Results:	100

Event#: 1 MS(E+) Ret. Time : 3.053 -> 3.587 Scan# : 459 -> 539

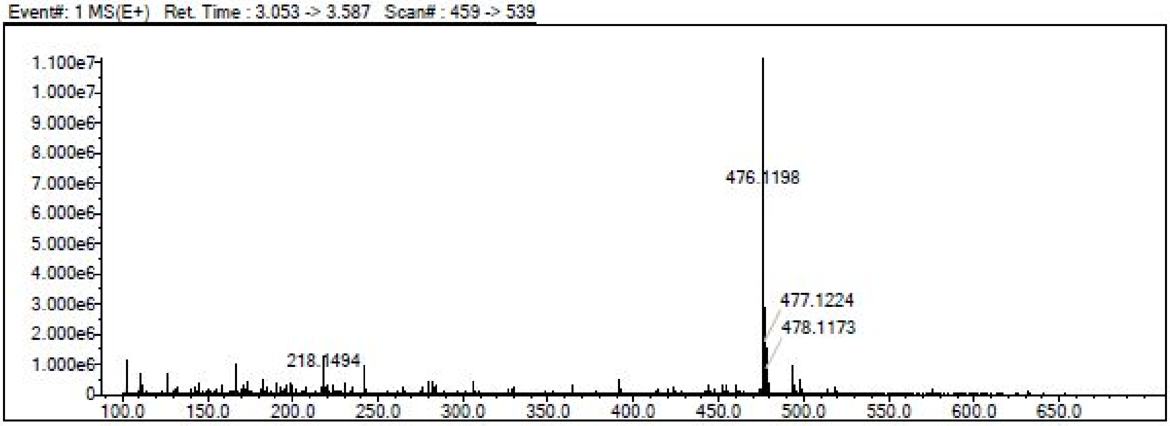

Measured region for 476.1198 m/z

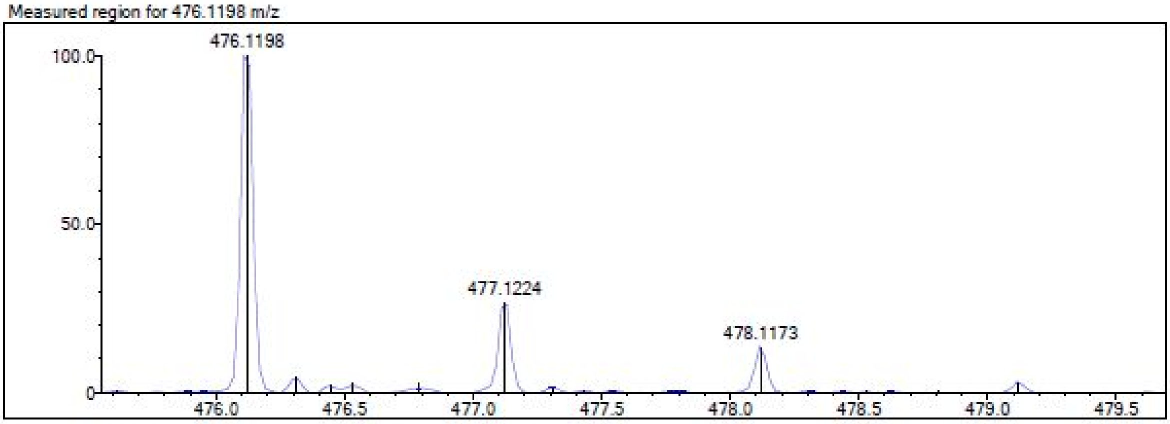

C21 H22 N5 O3 F S2 [M+H]+ : Predicted region for 476.1221 m/z

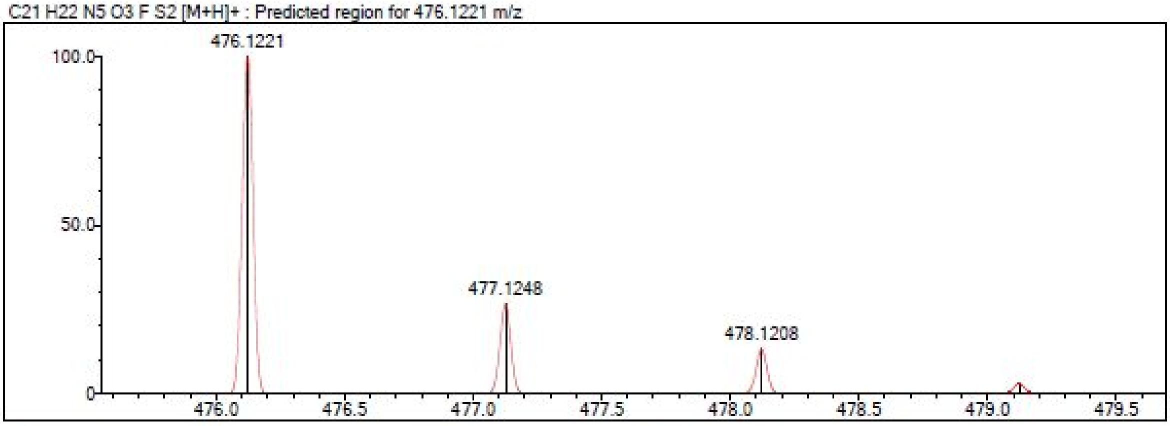

Rank	Score	Formula (M)	Ion	Meas. m/z	Pred. m/z	Df. (mDa)	Df. (ppm)	Iso	DBE
1	89.45	C21 H22 N5 O3 F S2	[M+H]+	476.1198	476.1221	-2.3	-4.83	98.92	13.0

Figure S42. Mass spectrum of compound **6n**

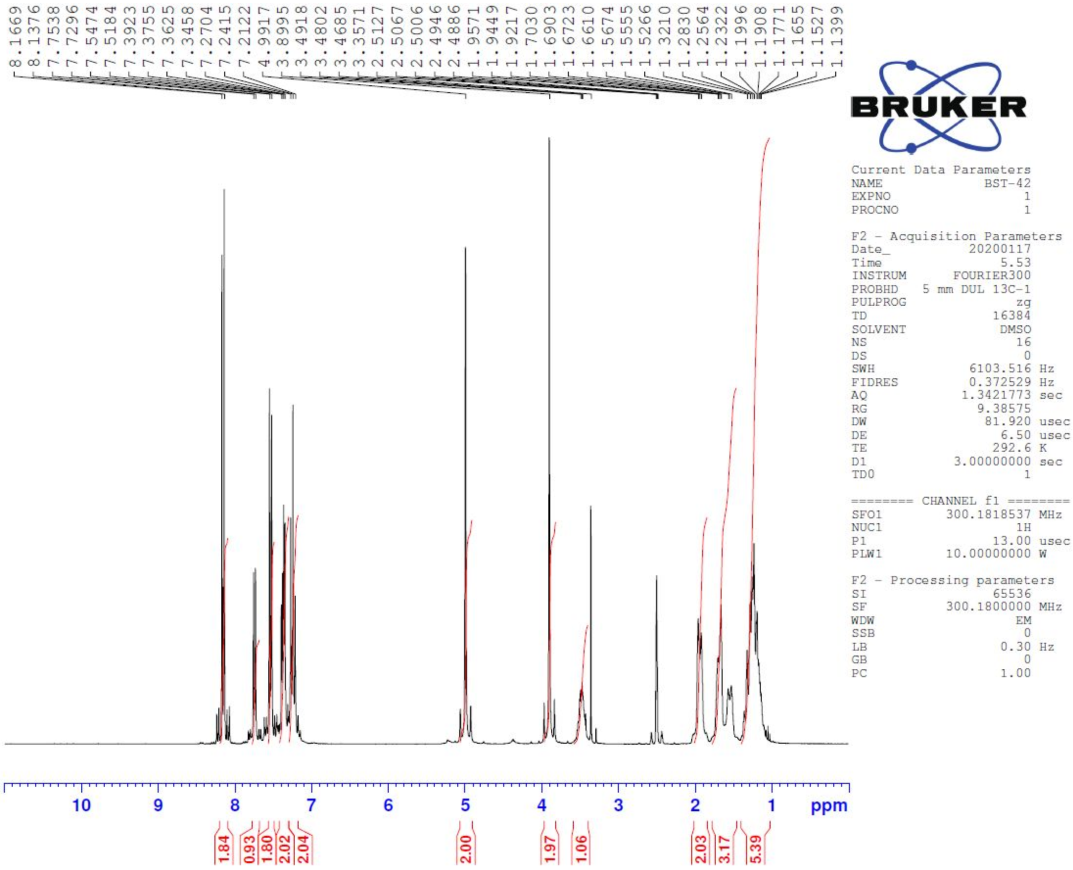

Figure S43. ^{1}H-NMR spectrum of compound **6o**

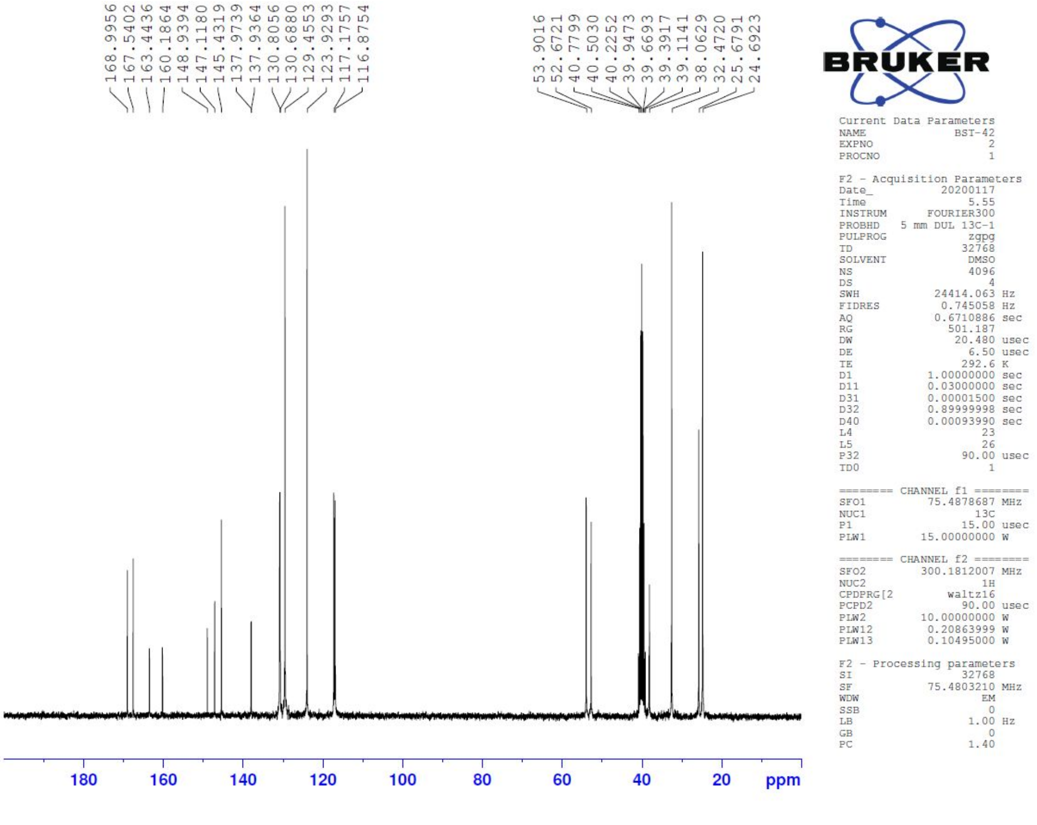

Figure S44. ^{13}C-NMR spectrum of compound **6o**

Formula Predictor Report - BST-42_30.lcd

Data File: C:\LabSolutions\Data\Analiz\uac\BST-42_30.lcd

Elmt	Val.	Min	Max	Elmt	Val.	Min	Max	Elmt	Val.	Min	Max	Elmt	Val.	Min	Max	Use Adduct
H	1	6	30	O	2	3	4	S	2	2	3	Ru	2	0	0	H
C	4	8	30	F	1	0	1	Cl	1	0	1	Pd	2	0	0	
N	3	4	5	P	3	0	0	Br	1	0	0	I	3	0	0	

Error Margin (ppm):	15	DBE Range:	10.0 - 20.0	Electron Ions:	both
HC Ratio:	unlimited	Apply N Rule:	yes	Use MSn Info:	yes
Max Isotopes:	3	Isotope RI (%):	1.00	Isotope Res:	9000
MSn Iso RI (%):	10.00	MSn Logic Mode:	AND	Max Results:	100

Event#: 1 MS(E+) Ret. Time : 3.640 -> 4.080 Scan# : 547 -> 613

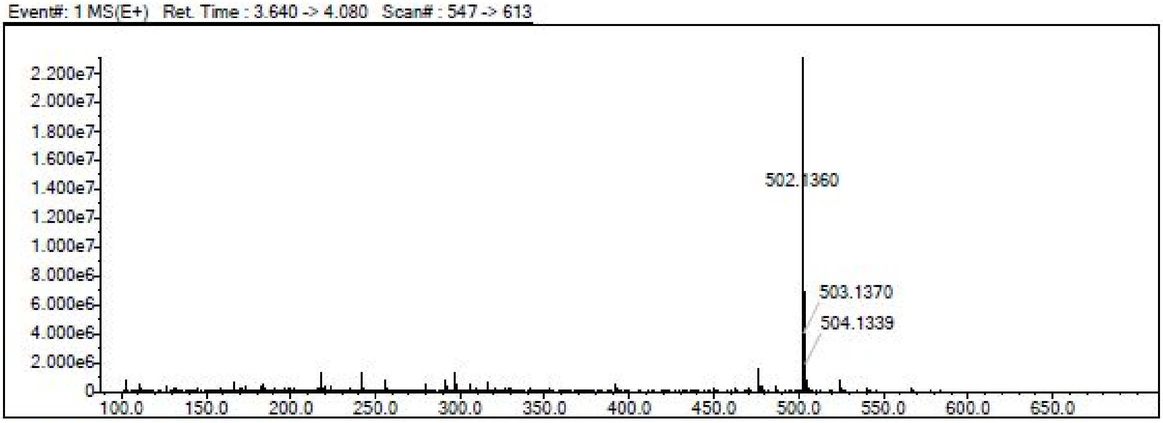

Measured region for 502.1360 m/z

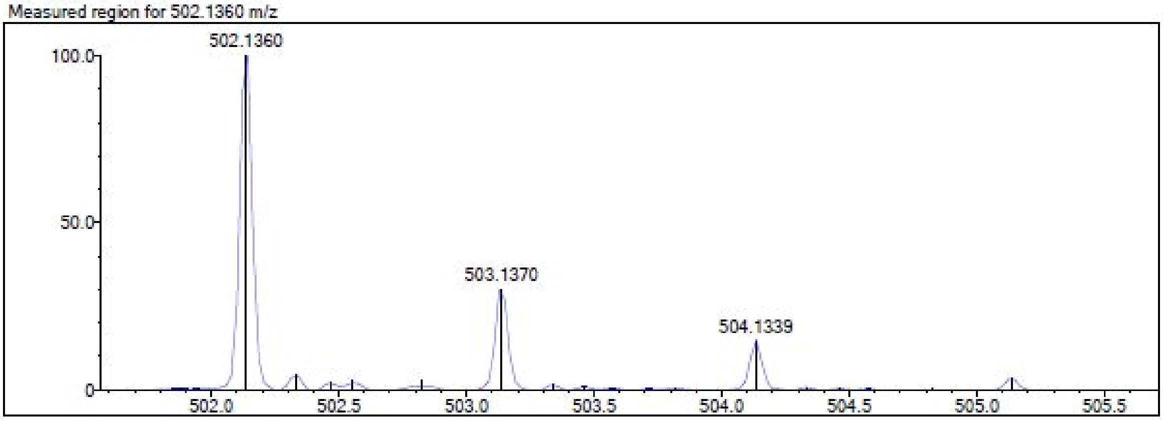

C23 H24 N5 O3 F S2 [M+H]+ : Predicted region for 502.1377 m/z

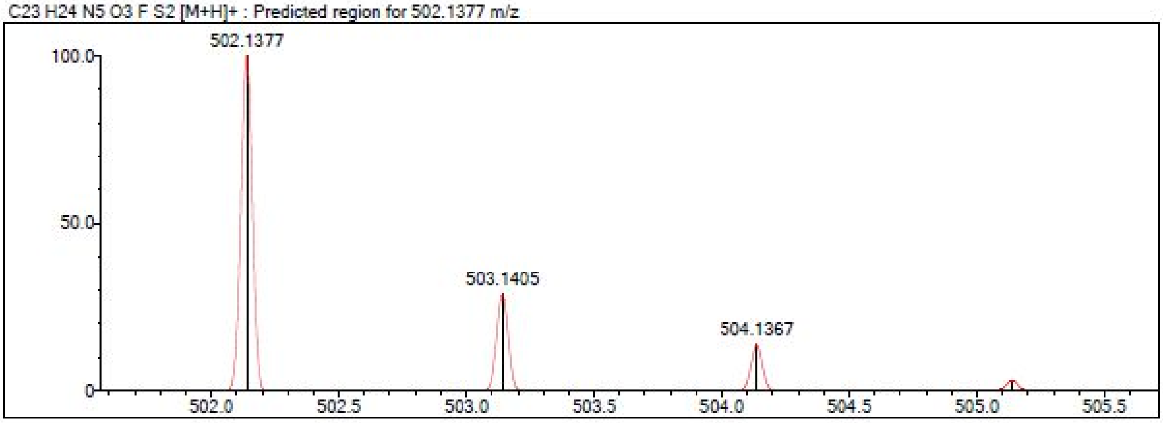

Rank	Score	Formula (M)	Ion	Meas. m/z	Pred. m/z	Df. (mDa)	Df. (ppm)	Iso	DBE
1	94.03	C23 H24 N5 O3 F S2	[M+H]+	502.1360	502.1377	-1.7	-3.39	100.00	14.0

Figure S45. Mass spectrum of compound **6o**

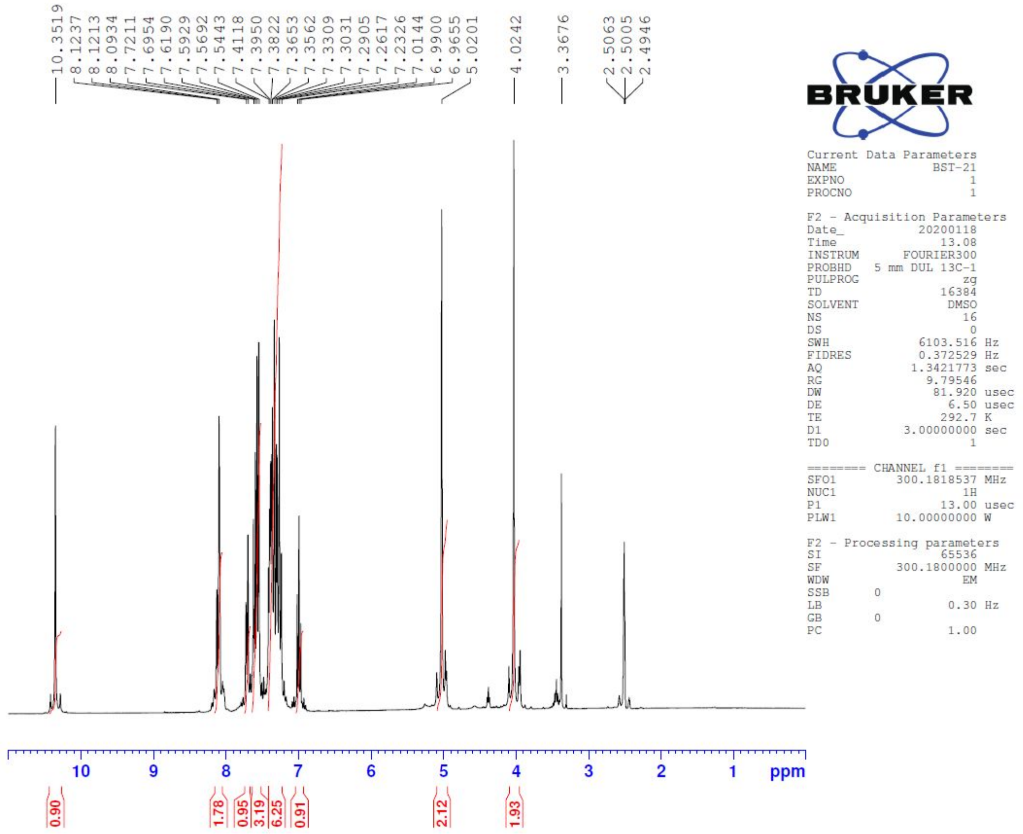

Figure S46. ^{1}H-NMR spectrum of compound **6p**

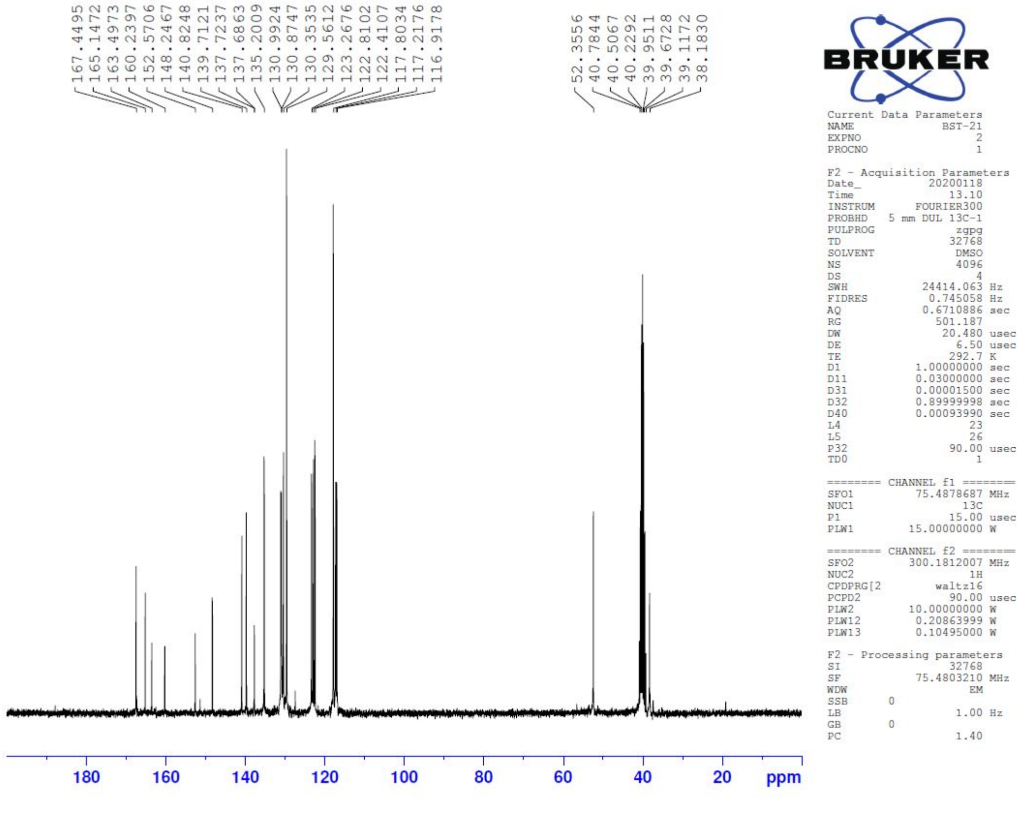

Figure S47. ^{13}C-NMR spectrum of compound **6p**

Data File: C:\LabSolutions\Data\Analiz\uac\BST-43A_31.lcd

Elmt	Val.	Min	Max	Elmt	Val.	Min	Max	Elmt	Val.	Min	Max	Elmt	Val.	Min	Max	Use Adduct
H	1	6	30	O	2	3	4	S	2	2	3	Ru	2	0	0	H
C	4	8	30	F	1	0	1	Cl	1	0	1	Pd	2	0	0	
N	3	4	5	P	3	0	0	Br	1	0	0	I	3	0	0	

Error Margin (ppm):	15	DBE Range:	13.0 - 20.0	Electron Ions:	both
HC Ratio:	unlimited	Apply N Rule:	yes	Use MSn Info:	yes
Max Isotopes:	3	Isotope RI (%):	1.00	Isotope Res:	9000
MSn Iso RI (%):	10.00	MSn Logic Mode:	AND	Max Results:	100

Event#: 1 MS(E+) Ret. Time : 2.987 Scan# : 449

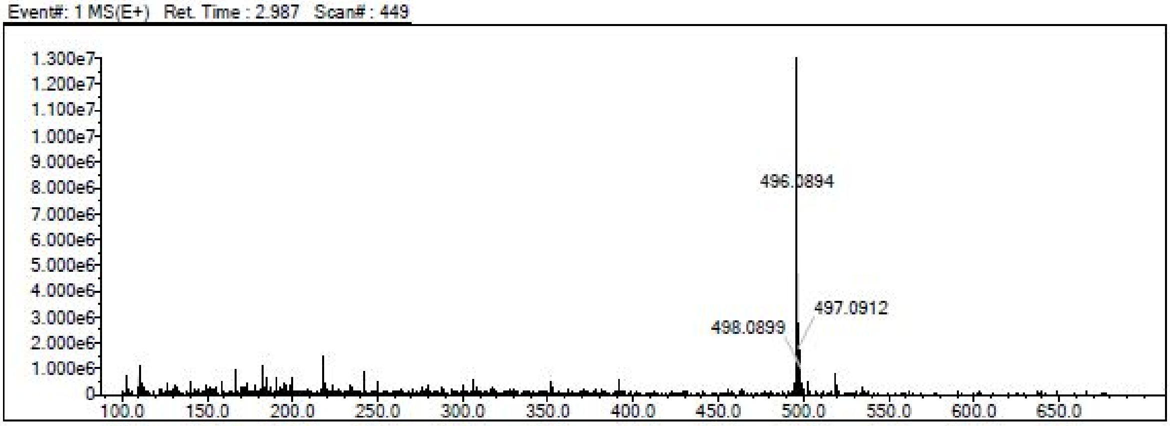

Measured region for 496.0894 m/z

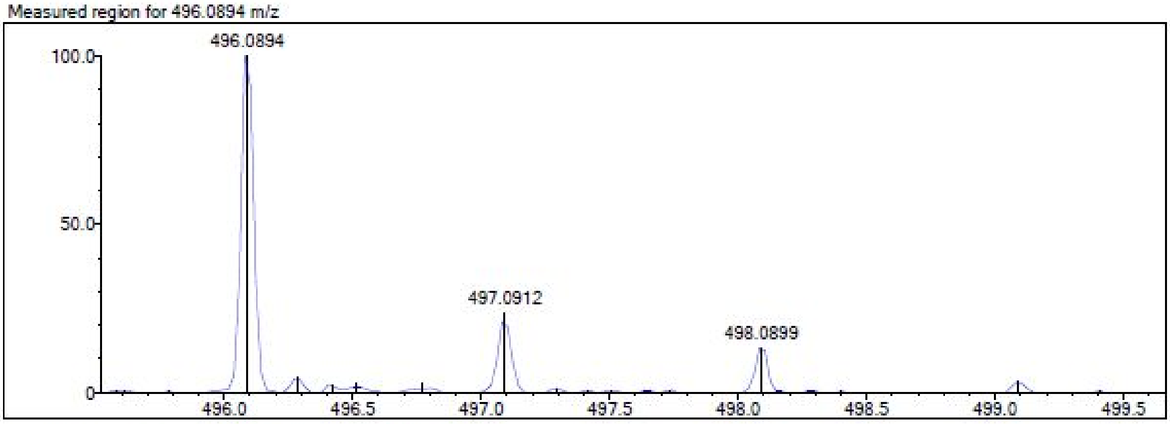

C23 H18 N5 O3 F S2 [M+H]+ : Predicted region for 496.0908 m/z

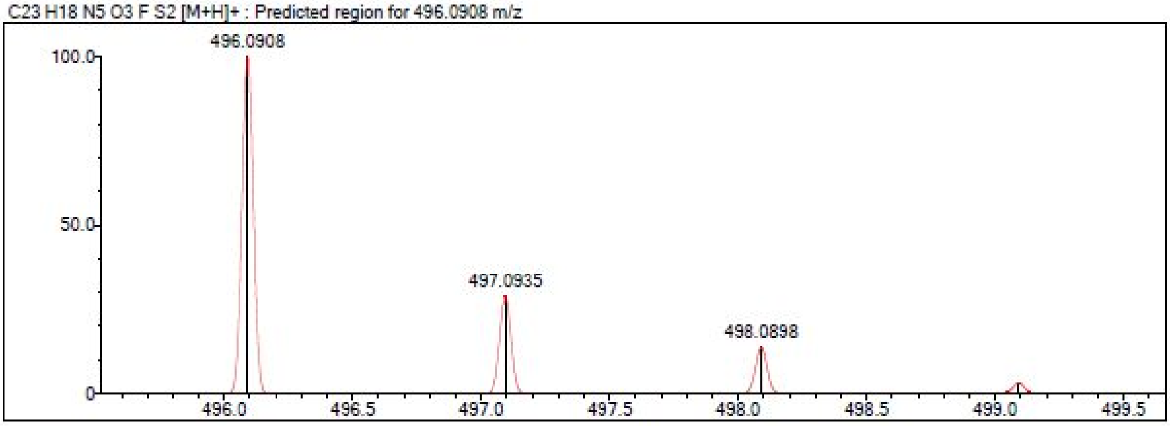

Rank	Score	Formula (M)	Ion	Meas. m/z	Pred. m/z	Df. (mDa)	Df. (ppm)	Iso	DBE
1	77.72	C23 H18 N5 O3 F S2	[M+H]+	496.0894	496.0908	-1.4	-2.82	81.43	17.0

Figure S48. Mass spectrum of compound **6p**

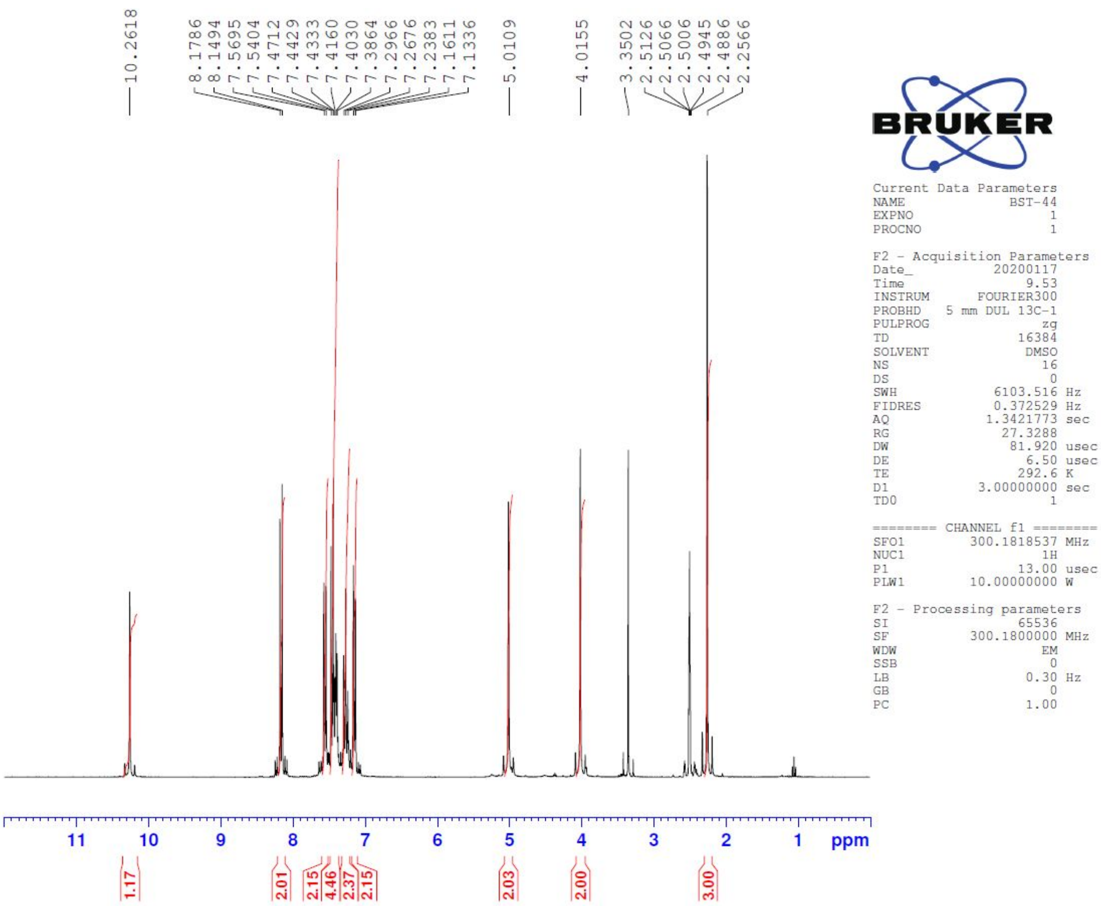

Figure S49. ^{1}H-NMR spectrum of compound **6q**

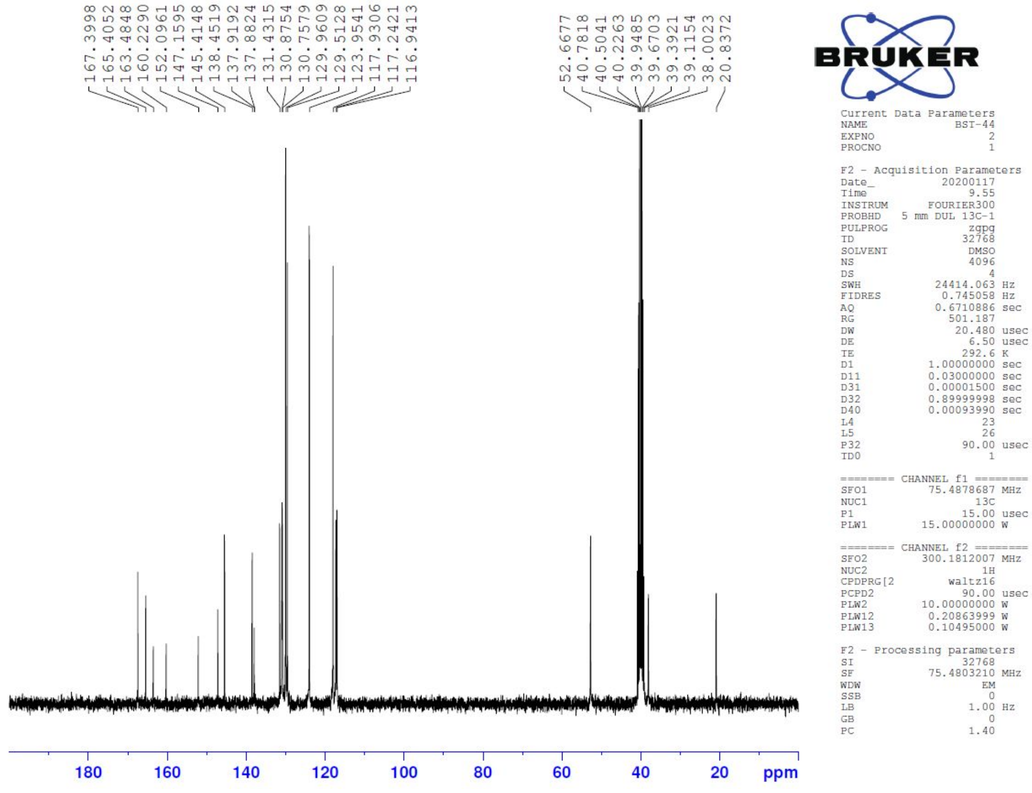

Figure S50. ^{13}C-NMR spectrum of compound **6q**

Data File: C:\LabSolutions\Data\Analiz\uac\BST-44_33.lcd

Elmt	Val.	Min	Max	Elmt	Val.	Min	Max	Elmt	Val.	Min	Max	Elmt	Val.	Min	Max	Use Adduct
H	1	6	30	O	2	3	4	S	2	2	3	Ru	2	0	0	H
C	4	8	30	F	1	0	1	Cl	1	0	1	Pd	2	0	0	
N	3	4	5	P	3	0	0	Br	1	0	0	I	3	0	0	

Error Margin (ppm):	15	DBE Range:	13.0 - 20.0	Electron Ions:	both
HC Ratio:	unlimited	Apply N Rule:	yes	Use MSn Info:	yes
Max Isotopes:	3	Isotope RI (%):	1.00	Isotope Res:	9000
MSn Iso RI (%):	10.00	MSn Logic Mode:	AND	Max Results:	100

Event#: 1 MS(E+) Ret. Time : 3.160 Scan# : 475

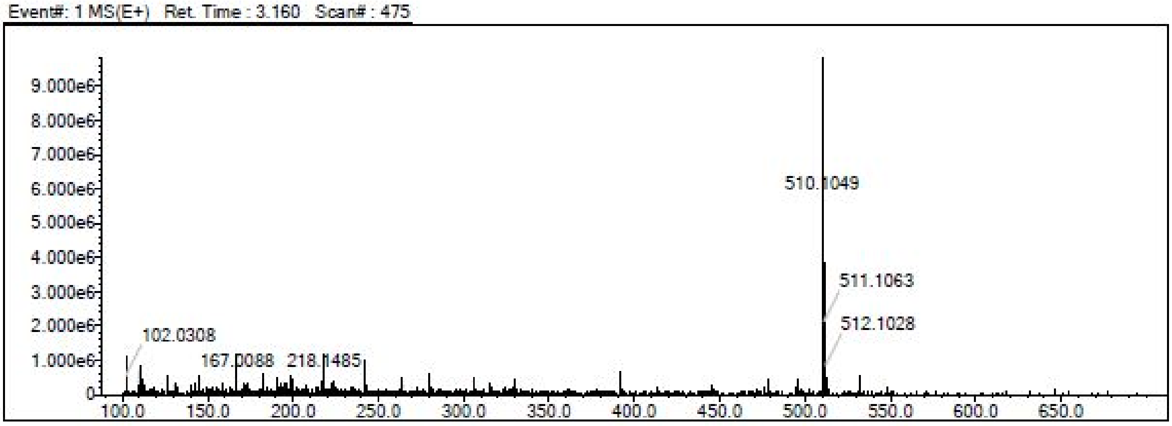

Measured region for 510.1049 m/z

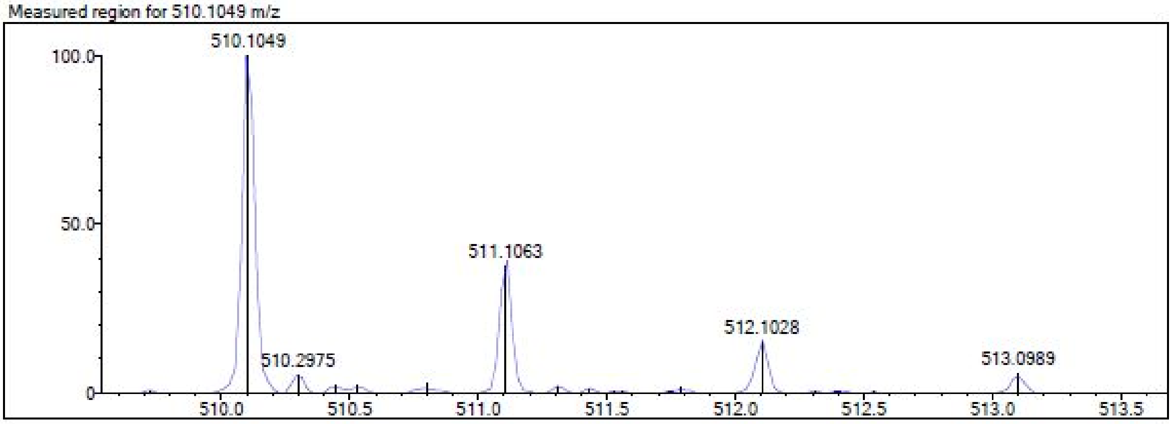

C24 H20 N5 O3 F S2 [M+H]+ : Predicted region for 510.1064 m/z

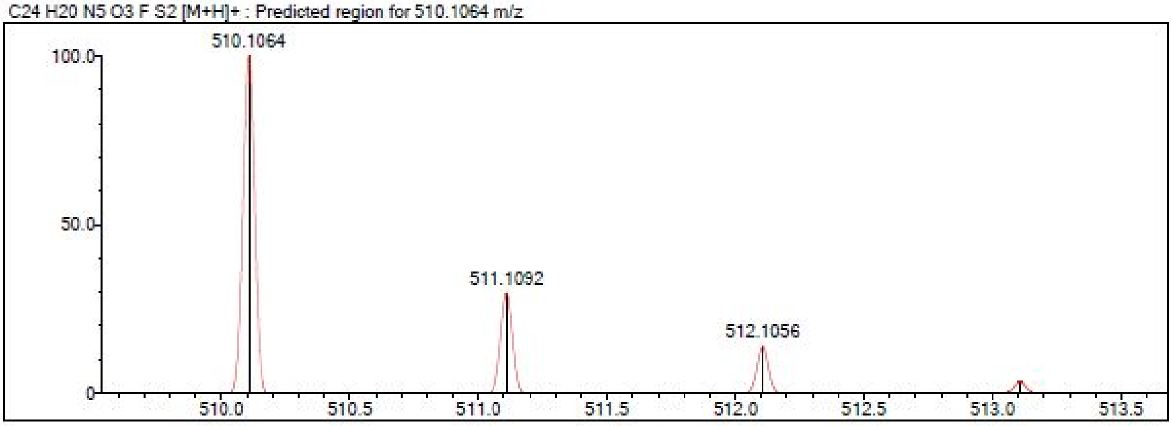

Rank	Score	Formula (M)	Ion	Meas. m/z	Pred. m/z	Df. (mDa)	Df. (ppm)	Iso	DBE
1	66.42	C24 H20 N5 O3 F S2	[M+H]+	510.1049	510.1064	-1.5	-2.94	69.81	17.0

Figure S51. Mass spectrum of compound **6q**
